# Fine scale mapping of the 17q22 breast cancer locus using dense SNPs, genotyped within the Collaborative Oncological Gene-Environment Study (COGs)

**DOI:** 10.1038/srep32512

**Published:** 2016-09-07

**Authors:** Hatef Darabi, Jonathan Beesley, Arnaud Droit, Siddhartha Kar, Silje Nord, Mahdi Moradi Marjaneh, Penny Soucy, Kyriaki Michailidou, Maya Ghoussaini, Hanna Fues Wahl, Manjeet K. Bolla, Qin Wang, Joe Dennis, M. Rosario Alonso, Irene L. Andrulis, Hoda Anton-Culver, Volker Arndt, Matthias W. Beckmann, Javier Benitez, Natalia V. Bogdanova, Stig E. Bojesen, Hiltrud Brauch, Hermann Brenner, Annegien Broeks, Thomas Brüning, Barbara Burwinkel, Jenny Chang-Claude, Ji-Yeob Choi, Don M. Conroy, Fergus J. Couch, Angela Cox, Simon S. Cross, Kamila Czene, Peter Devilee, Thilo Dörk, Douglas F. Easton, Peter A. Fasching, Jonine Figueroa, Olivia Fletcher, Henrik Flyger, Eva Galle, Montserrat García-Closas, Graham G. Giles, Mark S. Goldberg, Anna González-Neira, Pascal Guénel, Christopher A. Haiman, Emily Hallberg, Ute Hamann, Mikael Hartman, Antoinette Hollestelle, John L. Hopper, Hidemi Ito, Anna Jakubowska, Nichola Johnson, Daehee Kang, Sofia Khan, Veli-Matti Kosma, Mieke Kriege, Vessela Kristensen, Diether Lambrechts, Loic Le Marchand, Soo Chin Lee, Annika Lindblom, Artitaya Lophatananon, Jan Lubinski, Arto Mannermaa, Siranoush Manoukian, Sara Margolin, Keitaro Matsuo, Rebecca Mayes, James McKay, Alfons Meindl, Roger L. Milne, Kenneth Muir, Susan L. Neuhausen, Heli Nevanlinna, Curtis Olswold, Nick Orr, Paolo Peterlongo, Guillermo Pita, Katri Pylkäs, Anja Rudolph, Suleeporn Sangrajrang, Elinor J. Sawyer, Marjanka K. Schmidt, Rita K. Schmutzler, Caroline Seynaeve, Mitul Shah, Chen-Yang Shen, Xiao-Ou Shu, Melissa C. Southey, Daniel O. Stram, Harald Surowy, Anthony Swerdlow, Soo H. Teo, Daniel C. Tessier, Ian Tomlinson, Diana Torres, Thérèse Truong, Celine M. Vachon, Daniel Vincent, Robert Winqvist, Anna H. Wu, Pei-Ei Wu, Cheng Har Yip, Wei Zheng, Paul D. P. Pharoah, Per Hall, Stacey L. Edwards, Jacques Simard, Juliet D. French, Georgia Chenevix-Trench, Alison M. Dunning

**Affiliations:** 1 Department of Medical Epidemiology and Biostatistics, Karolinska Institutet, Stockholm, Sweden; 2 Department of Genetics, QIMR Berghofer Medical Research Institute, Brisbane, Australia; 3 Département de Médecine Moléculaire, Faculté de Médecine, Centre Hospitalier Universitaire de Québec Research Center, Laval University, Québec City, Canada; 4 Centre for Cancer Genetic Epidemiology, Department of Oncology, University of Cambridge, Cambridge, UK; 5 Department of Cancer Genetics, Institute for Cancer Research, Oslo University Hospital Radiumhospitalet, Oslo, Norway; 6 Genomics Center, Centre Hospitalier Universitaire de Québec Research Center, Laval University, Québec City, Canada; 7 Centre for Cancer Genetic Epidemiology, Department of Public Health and Primary Care, University of Cambridge, Cambridge, UK; 8 Department of Electron Microscopy/Molecular Pathology, The Cyprus Institute of Neurology and Genetics, Nicosia, Cyprus; 9 Human Genotyping-CEGEN Unit, Human Cancer Genetic Program, Spanish National Cancer Research Centre, Madrid, Spain; 10 Lunenfeld-Tanenbaum Research Institute of Mount Sinai Hospital, Toronto, Canada; 11 Department of Molecular Genetics, University of Toronto, Toronto, Canada; 12 Department of Epidemiology, University of California Irvine, Irvine, CA, USA; 13 Division of Clinical Epidemiology and Aging Research, German Cancer Research Center (DKFZ), Heidelberg, Germany; 14 Department of Gynaecology and Obstetrics, University Hospital Erlangen, Friedrich-Alexander University Erlangen-Nuremberg, Comprehensive Cancer Center Erlangen-EMN, Erlangen, Germany; 15 Human Cancer Genetics Program, Spanish National Cancer Research Centre, Madrid, Spain; 16 Centro de Investigación en Red de Enfermedades Raras, Valencia, Spain; 17 Department of Radiation Oncology, Hannover Medical School, Hannover, Germany; 18 Copenhagen General Population Study, Herlev and Gentofte Hospital, Copenhagen University Hospital, Herlev, Denmark; 19 Department of Clinical Biochemistry, Herlev and Gentofte Hospital, Copenhagen University Hospital, Herlev, Denmark; 20 Faculty of Health and Medical Sciences, University of Copenhagen, Copenhagen, Denmark; 21 Dr. Margarete Fischer-Bosch-Institute of Clinical Pharmacology, Stuttgart, Germany; 22 University of Tübingen, Tübingen, Germany; 23 German Cancer Consortium (DKTK), German Cancer Research Center (DKFZ), Heidelberg, Germany; 24 Division of Preventive Oncology, German Cancer Research Center (DKFZ) and National Center for Tumor Diseases (NCT), Heidelberg, Germany; 25 Netherlands Cancer Institute, Antoni van Leeuwenhoek hospital, Amsterdam, The Netherlands; 26 Institute for Prevention and Occupational Medicine of the German Social Accident Insurance, Institute of the Ruhr University Bochum, Bochum, Germany; 27 Department of Obstetrics and Gynecology, University of Heidelberg, Heidelberg, Germany; 28 Molecular Epidemiology Group, German Cancer Research Center (DKFZ), Heidelberg, Germany; 29 Division of Cancer Epidemiology, German Cancer Research Center (DKFZ), Heidelberg, Germany; 30 University Cancer Center Hamburg (UCCH), University Medical Center Hamburg-Eppendorf, Hamburg, Germany; 31 Department of Biomedical Sciences, Seoul National University College of Medicine, Seoul, Korea; 32 Cancer Research Institute, Seoul National University, Seoul, Korea; 33 Department of Laboratory Medicine and Pathology, Mayo Clinic, Rochester, MN, USA; 34 Sheffield Cancer Research, Department of Oncology and Metabolism, University of Sheffield, Sheffield, UK; 35 Academic Unit of Pathology, Department of Neuroscience, University of Sheffield, Sheffield, UK; 36 Department of Pathology, Leiden University Medical Center, Leiden, The Netherlands; 37 Department of Human Genetics, Leiden University Medical Center, Leiden, The Netherlands; 38 Gynaecology Research Unit, Hannover Medical School, Hannover, Germany; 39 David Geffen School of Medicine, Department of Medicine Division of Hematology and Oncology, University of California at Los Angeles, Los Angeles, CA, USA; 40 Usher Institute of Population Health Sciences and Informatics, The University of Edinburgh Medical School, Edinburgh, UK; 41 Division of Cancer Epidemiology and Genetics, National Cancer Institute, Rockville, MD, USA; 42 Breakthrough Breast Cancer Research Centre, The Institute of Cancer Research, London, UK; 43 Division of Breast Cancer Research, The Institute of Cancer Research, London, UK; 44 Department of Breast Surgery, Herlev and Gentofte Hospital, Copenhagen University Hospital, Herlev, Denmark; 45 Vesalius Research Center, Leuven, Belgium; 46 Laboratory for Translational Genetics, Department of Oncology, University of Leuven, Leuven, Belgium; 47 Cancer Epidemiology Centre, Cancer Council Victoria, Melbourne, Australia; 48 Centre for Epidemiology and Biostatistics, Melbourne School of Population and Global health, The University of Melbourne, Melbourne, Australia; 49 Department of Medicine, McGill University, Montreal, Canada; 50 Division of Clinical Epidemiology, Royal Victoria Hospital, McGill University, Montreal, Canada; 51 Cancer & Environment Group, Center for Research in Epidemiology and Population Health (CESP), INSERM, University Paris-Sud, University Paris-Saclay, Villejuif, France; 52 Department of Preventive Medicine, Keck School of Medicine, University of Southern California, Los Angeles, CA, USA; 53 Department of Health Sciences Research, Mayo Clinic, Rochester, MN, USA; 54 Molecular Genetics of Breast Cancer, German Cancer Research Center (DKFZ), Heidelberg, Germany; 55 Saw Swee Hock School of Public Health, National University of Singapore, Singapore, Singapore; 56 Department of Surgery, National University Health System, Singapore, Singapore; 57 Department of Medical Oncology, Family Cancer Clinic, Erasmus MC Cancer Institute, Rotterdam, The Netherlands; 58 Division of Epidemiology and Prevention, Aichi Cancer Center Research Institute, Nagoya, Japan; 59 Department of Genetics and Pathology, Pomeranian Medical University, Szczecin, Poland; 60 Department of Preventive Medicine, Seoul National University College of Medicine, Seoul, Korea; 61 Department of Obstetrics and Gynecology, Helsinki University Hospital, University of Helsinki, Helsinki, Finland; 62 Cancer Center of Eastern Finland, University of Eastern Finland, Kuopio, Finland; 63 Institute of Clinical Medicine, Pathology and Forensic Medicine, University of Eastern Finland, Kuopio, Finland; 64 Imaging Center, Department of Clinical Pathology, Kuopio University Hospital, Kuopio, Finland; 65 K.G. Jebsen Center for Breast Cancer Research, Institute of Clinical Medicine, Faculty of Medicine, University of Oslo, Oslo, Norway; 66 Department of Clinical Molecular Biology, Oslo University Hospital, University of Oslo, Oslo, Norway; 67 University of Hawaii Cancer Center, Honolulu, HI, USA; 68 Department of Hematology-Oncology, National University Health System, Singapore, Singapore.; 69 Cancer Science Institute of Singapore, National University of Singapore, Singapore, Singapore; 70 Department of Molecular Medicine and Surgery, Karolinska Institutet, Stockholm, Sweden.; 71 Division of Health Sciences, Warwick Medical School, Warwick University, Coventry, UK; 72 Unit of Molecular Bases of Genetic Risk and Genetic Testing, Department of Preventive and Predictive Medicine, Fondazione IRCCS (Istituto Di Ricovero e Cura a Carattere Scientifico) Istituto Nazionale dei Tumori (INT), Milan, Italy; 73 Department of Oncology - Pathology, Karolinska Institutet, Stockholm, Sweden; 74 Division of Molecular Medicine, Aichi Cancer Center Research Institute, Nagoya, Japan; 75 International Agency for Research on Cancer, Lyon, France; 76 Division of Gynaecology and Obstetrics, Technische Universität München, Munich, Germany; 77 Institute of Population Health, University of Manchester, Manchester, UK; 78 Department of Population Sciences, Beckman Research Institute of City of Hope, Duarte, CA, USA; 79 IFOM, The FIRC (Italian Foundation for Cancer Research) Institute of Molecular Oncology, Milan, Italy; 80 Laboratory of Cancer Genetics and Tumor Biology, Cancer and Translational Medicine Research Unit, Biocenter Oulu, University of Oulu, Oulu, Finland; 81 Laboratory of Cancer Genetics and Tumor Biology, Northern Finland Laboratory Centre Oulu, Oulu, Finland; 82 National Cancer Institute, Bangkok, Thailand; 83 Research Oncology, Guy's Hospital, King's College London, London, UK; 84 Center for Hereditary Breast and Ovarian Cancer, University Hospital of Cologne, Cologne, Germany; 85 Center for Integrated Oncology (CIO), University Hospital of Cologne, Cologne, Germany; 86 Center for Molecular Medicine Cologne (CMMC), University of Cologne, Cologne, Germany; 87 Institute of Biomedical Sciences, Academia Sinica, Taipei, Taiwan; 88 Taiwan Biobank, Institute of Biomedical Sciences, Academia Sinica, Taipei, Taiwan; 89 Division of Epidemiology, Department of Medicine, Vanderbilt-Ingram Cancer Center, Vanderbilt University School of Medicine, Nashville, TN, USA; 90 Department of Pathology, The University of Melbourne, Melbourne, Australia; 91 Division of Genetics and Epidemiology, The Institute of Cancer Research, London, UK; 92 Cancer Research Initiatives Foundation, Subang Jaya, Selangor, Malaysia; 93 Breast Cancer Research Unit, Cancer Research Institute, University Malaya Medical Centre, Kuala Lumpur, Malaysia; 94 McGill University and Génome Québec Innovation Centre, Montréal, Canada; 95 Wellcome Trust Centre for Human Genetics and Oxford NIHR Biomedical Research Centre, University of Oxford, Oxford, UK; 96 Institute of Human Genetics, Pontificia Universidad Javeriana, Bogota, Colombia

## Abstract

Genome-wide association studies have found SNPs at 17q22 to be associated with breast cancer risk. To identify potential causal variants related to breast cancer risk, we performed a high resolution fine-mapping analysis that involved genotyping 517 SNPs using a custom Illumina iSelect array (iCOGS) followed by imputation of genotypes for 3,134 SNPs in more than 89,000 participants of European ancestry from the Breast Cancer Association Consortium (BCAC). We identified 28 highly correlated common variants, in a 53 Kb region spanning two introns of the *STXBP4* gene, that are strong candidates for driving breast cancer risk (lead SNP rs2787486 (OR = 0.92; CI 0.90–0.94; *P* = 8.96 × 10^−15^)) and are correlated with two previously reported risk-associated variants at this locus, SNPs rs6504950 (OR = 0.94, *P* = 2.04 × 10^−09^, r^2^ = 0.73 with lead SNP) and rs1156287 (OR = 0.93, *P* = 3.41 × 10^−11^, r^2^ = 0.83 with lead SNP). Analyses indicate only one causal SNP in the region and several enhancer elements targeting *STXBP4* are located within the 53 kb association signal. Expression studies in breast tumor tissues found SNP rs2787486 to be associated with increased *STXBP4* expression, suggesting this may be a target gene of this locus.

Breast cancer is one of the most common epithelial malignancies in women^1^,^2^. Ahmed *et al*.^3^ carried out a multi-stage genome wide association study (GWAS) for breast cancer susceptibility involving studies from the Cancer Genetic Markers of Susceptibility (CGEMS) and Breast Cancer Association Consortium (BCAC) and reported strong evidence for a susceptibility locus at 17q22 with single nucleotide polymorphism (SNP) rs6504950, OR = 0.95, 95% confidence interval (CI) 0.92–0.97, *P* = 1.4 × 10^−8^. Turnbull *et al*.^4^ found confirmatory evidence for association with SNPs at the same locus; they reported a breast cancer risk association with SNP rs1156287 (OR = 0.91; 95% CI 0.85–0.97; *P* = 5.8 × 10^−3^), which lies 20 kb from originally reported SNP rs6504950 (r^2^ = 0.91). Using data from the National Cancer Institute's Breast and Prostate Cancer Cohort Consortium (BPC3), Campa *et al*.^5^ also confirmed the association with rs6504950 (OR = 0.92; 95% CI 0.88–0.97; *P* = 5.83 × 10^−4^). Broeks *et al*.^6^ further investigated this association with respect to tumor estrogen receptor (ER) status, and reported that rs6504950 had stronger association with ER positive (ER+; OR = 0.93; 95% CI 0.90–0.95; *P* = 7.2 × 10^−7^) than ER negative disease (ER−; OR = 1.00; 95% CI 0.95–1.05; *P* = 0.94). Tang *et al*.^7^ conducted a meta-analysis which further confirmed the association with SNP rs6504950 (OR = 0.93; 95% CI 0.87–0.99). SNP rs6504950 lies in an intron of *STXBP4* (Syntax binding protein 4) and two other genes are found within 200 kb including *COX11* (cytochrome C assembly protein 11) and *TOM1L1* (target of myb1-like1). As part of the Collaborative Oncological Gene-Environment Study (COGS), we conducted a comprehensive fine-scale mapping of this 17q22 breast cancer susceptibility locus using 517 SNPs chosen to give dense coverage across this locus. These were genotyped on a custom-designed Illumina iSelect genotyping array (iCOGS) in 50 studies participating in BCAC. We used these data to define the variants most strongly associated with risk, and combined these data with additional *in-silico* and functional data in an attempt to determine the most likely causal variants.

## Material and Methods

### Genetic Mapping

#### Tagging strategy for the fine-scale mapping

We defined the region for fine-mapping by identifying the flanking SNPs with minor allele frequency (MAF) > 2% and detectable correlation (r^2^ > 0.1) with rs6504950, based on the 1000 genomes project European population (March 2010 Pilot version 60 CEU project data). From this 468 kb interval we selected all SNPs correlated with rs6504950 at r^2^ > 0.1, plus a set of SNPs designed to tag all remaining SNPs with r^2^ > 0.9. We thus aimed to genotype 525 SNPs, between chromosome 17 positions 52,816,899 and 53,284,506 (NCBI build 37 assembly), that had an Illumina designability score (DS) > 0.9. Of these, 517 were successfully genotyped on the array and passed QC filters.

#### iCOGS genotyping and imputation

Case and control samples were drawn from studies participating in the BCAC, of which 41 (total: 46450 cases/42600 controls) were predominantly of European ancestry and nine (6269 cases/6624 controls) of Asian ancestry. We performed iCOGS genotyping in four centres, as part of the Collaborative Oncological Gene-Environment Study (COGS). All BCAC studies had local human ethical approvals as described previously^8^. We then used the genotype data from 517 SNPs that passed quality control to impute genotypes, among European subjects, at all additional known variants in the interval, using IMPUTE version 2.0 (IMPUTE2; without pre-phasing) and the 1000 genome project multi-population data (March 2012 version) as a reference panel^9^,^10^. IMPUTE2 was run with default parameters and "effective size" of the population Ne = 20,000. Using an imputation–r^2^ > 0.3 in Europeans, we successfully imputed 3,134 SNPs (MAF ≥ 1%).

### Statistical Analysis

For each SNP, we estimated the per-allele log-odds ratio (OR) and standard error using logistic regression, including principal components and per-study fixed-effects to capture study-specific differences as previously described^8^. For the analyses of European subjects, we included the first six principal components as covariates, together with a seventh component derived specifically for one study (LMBC) for which there was substantial inflation not accounted for by the components derived from the analysis of all studies (this component was set to zero for all other studies). For the analysis of Asian subjects, we included two principal components^8^. We estimated per allele ORs under the assumption of a log-additive mode of inheritance, i.e. SNPs were coded according to the number of minor alleles 0, 1 or 2. We estimated main effects by subtype specific status (ER +/−) using case-control logistic regression and restricting the case sample to a specific subtype. We evaluated heterogeneity of association across tumour subtypes in a case-only analysis, treating subtype status as a dependent variable. We derived the *P* values by means of a likelihood-ratio test (one degree of freedom). Tests were two-sided. We carried out analyses separately among women of European and of Asian ancestry, defined by multiple dimensional scaling as previously described^8^. We performed multiple logistic regression analyses to identify SNPs independently associated with each phenotype. To identify the most parsimonious model, we included all SNPs with a *P* < 10^−4^ and MAF ≥ 2% in the single SNP analysis in forward selection regression analyses, utilizing the step function in R^11^ with penalty term set to 20^12^; we also used a joint analysis of all SNPs using a Bayesian-inspired penalised maximum likelihood approach (HyperLasso)^13^. To correctly account for uncertainty in the data resulting from the imputation process, we conducted analysis by regressing on the allele dosage for each genotype. For HyperLasso we utilized the most probable genotype as input, based on the posterior probability from the imputation algorithm (set to missing if all posterior probabilities were <0.9).

### Bioinformatic analyses

We combined multiple sources of *in silico* annotation from public databases to help identify potential functional SNPs. To investigate functional elements enriched across the previously defined fine-mapped region, more specifically in the region encompassing the strongest candidate causal SNPs, we analysed chromatin biofeatures data from the Encyclopedia of DNA Elements (ENCODE) Project^14^ namely: Chromatin State Segmentation by Hidden Markov Models (chromHMM), DNase I hypersensitivity sites (DHS) and histone modifications of epigenetic markers H3K4, H3K9, and H3K27 in Human Mammary Epithelial Cells (HMEC) and MCF7 breast cancer cells. To identify putative target genes, we examined chromatin interactions between distal and proximal regulatory transcription-factor binding sites and gene promoters, using Chromatin Interaction Analysis by Paired End Tag (ChiA-PET) in MCF7 cells. This detects genome-wide interactions associated with CCCTC-binding factor (CTCF) and DNA polymerase II (Pol2) – both involved in transcriptional regulation^15^. Putative regulatory elements were determined using data from ENCODE, Roadmap Epigenomics^16^, the “Predicting Specific Tissue Interactions of Genes and Enhancers” (PreSTIGE)^17^ algorithm, Hnisz^18^ and FANTOM. Intersections between candidate causal variants and regulatory elements were identified using Galaxy, and visualised in the UCSC Genome Browser. We used the ENCODE RNAseq data to evaluate the expression of exons across the 17q22 locus in HMEC and MCF7 cell lines. The alignment files for HMEC (4 biological replicates) and MCF7 (19 biological replicates) were downloaded from ENCODE and the read count in the defined region was extracted and normalized in reads per million (RPM).

### Allele specific expression (ASE) analysis

ASE analysis was performed using The Cancer Genome Atlas (TCGA) breast cancer data as described previously^19^. SNP rs2787481, genotyped on the Affymetrix SNP Array 6.0 was used as a representative SNP for the candidate causal variants (r^2^ = 0.90 with rs2787486). SNP rs2787481 genotype calls and the corresponding confidence scores were retrieved using level 2 TCGA SNP array Birdseed data downloaded from TCGA portal. Genotypes with confidence scores equal to or above 0.1 were excluded.

We utilised RNA-sequencing data from 742 breast cancer samples from women of Caucasian ancestry. The corresponding RNA-sequencing BAM files and metadata are available from the Cancer Genomics Hub (CGHub). Markers used to assess relative allelic expression were exonic SNPs located in *KIF2B, TOM1L1, COX11, STXBP4, HLF, MMD, TMEM100, PCTP,* and *ANKFN1* extracted from dbSNP human Build 142. Homozygote marker SNPs, those with low coverage (less than 15x) and those within overlapping regions of the target genes, were removed. RNA-sequencing read counts on SNP sites for reference and alternative alleles were computed. The major allele fraction (μ), representing allelic imbalance for each marker SNP, was computed and an average of allelic imbalances for each gene was calculated for individual tumour samples. Marker SNPs with extreme μ values (μ > 0.75) were not included in the analysis. Level 3 SNP array data were downloaded from TCGA portal and GISTIC version 2.0.16 was used to identify copy number variations (CNVs) for each sample. Samples with low or high CNV levels, as presented in the gene-based GISTIC module report, were excluded from the analysis of the corresponding gene.

Allelic imbalance for the target transcripts was compared between rs2787481 heterozygote (CT) and homozygote (CC and TT) samples using Levene's Test for equality of variances. *KIF2B, TMEM100,* and *ANKFN1* were excluded from the statistical analyses as they did not have enough informative marker SNPs left after applying the filtering criteria.

### Local gene expression by SNP (eQTL) association analysis

We examined the association of all genotyped or imputed SNPs with expression of nine genes (*KIF2B, TOM1L1, COX11, STXBP4, HLF, MMD, TMEM100, PCTP,* and *ANKFN1*) in the 1 Mb region on either side of the fine-mapping interval, using data from the Molecular Taxonomy of Breast Cancer International Consortium (METABRIC) study. METABRIC comprises normal tissues adjacent to tumours from breast cancer patients genetically confirmed to be of European ancestry^20^. The samples (n = 135) were assayed for expression with the Illumina HT-12 v3 microarray. Matched germline SNP genotypes were derived using the Affymetrix SNP 6.0 array. Genotyping quality control and imputation for the METABRIC data are described in Guo *et al*.^21^. Association between genotype and expression was tested by linear regression with FDR control as implemented in the MatrixEQTL^22^ package in R^11^.

Four additional SNP-expression data sets were available and analysed separately: (1) NB116 consists of 116 Caucasian normal breast samples (the majority of Norwegian descent) with n = 10 tumour-adjacent normal biopsies. (2) BC241 consists of 241 Caucasian tumor (all stages) samples (the majority of Norwegian origin). These were both genotyped on the “iCOGS” SNP array, and gene expression levels were measured with Agilent 44 K^23^. (3) NB93 consists of 93 Caucasian adjacent normal breast samples from TCGA. Birdseed processed germline genotype data from the Affy6 SNP array were obtained from the TCGA dbGaP data portal^24^. (4) BC765 consists of 765 Caucasian breast tumour samples from TCGA^24^. Gene expression levels were assayed by RNA sequencing, RSEM (RNAseq by Expectation-Maximization^25^) normalized per gene or isoform, as obtained from the TCGA consortium^24^. Unexpressed and minimally expressed genes/isoforms whose sum in expression level was less than ten were excluded, and the data log2 transformed prior to analysis. The influence of SNPs on local gene expression (transcripts within 1 MB from the most strongly associated SNP) was assessed using a linear regression model, as implemented in the R^11^ library eMAP^26^. An additive effect was assumed by modelling the patient’s number of copies of the rare allele, i.e. 0, 1 or 2 for a given genotype. Correction for multiple testing was performed using the false discovery rate (FDR) as implemented in the p.adjust function in R.

eQTL data from the Genotype-Tissue Expression (GTEx) project^27^ were downloaded from the v6 release.

## Results

**Figure 1:**
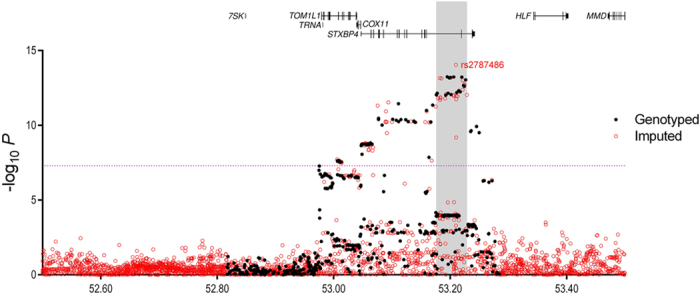
Association Results for Overall Breast Cancer Risk. Directly genotyped SNPs are shown as filled black circles, and imputed SNPs (r^2^ > 0.3, MAF > 0.02) are shown as open red circles, plotted as the negative log of the *P* value against relative position across the locus. A schematic of the gene structures is shown. Signals, encompassing all SNPs with a likelihood ratio of <1:100 compared with the most significant SNP, are labelled and are shown as grey regions. The dashed purple line represents genome-wide significance (*P *< 5 × 10^−08^).

A total of 517 SNPs at chromosome 17 positions 52,816,899 to 53,284,506 (NCI build 37 assembly) were successfully genotyped using the iCOGs chip. Genotypes of other common variants across the region were imputed in the European studies using known genotypes in combination with a reference panel from the 1000 Genomes Project. 3,134 SNPs and insertion/deletion (indel) polymorphisms were reliably imputed (imputation r^2^ score > 0.3, MAF ≥ 0.01) and included in further analysis together with the 517 genotyped SNPs. In the European studies 139 genotyped or imputed SNPs were associated with overall risk of breast cancer (*P* values < 10^−7^) (Fig. 1). This set included SNPs rs6504950 and rs1156287 (r^2^ = 0.84), both previously reported^3^,^4^ to be associated with breast cancer risk among Europeans (Supplementary Table 1).

Among the European ancestry studies the strongest association detected was with imputed SNP rs2787486 (OR [minor/major allele] = 0.92 [C/A]; 95% CI 0.90–0.94; *P* = 8.96 × 10^−15^), located in an intron of *STXBP4* and strongly correlated with both previously reported GWAS hits (r^2^ = 0.83 with rs1156287; r^2^ = 0.73 with rs6504950). The strongest genotyped SNP association was rs244353 (OR = 0.92; 95% CI 0.90–0.94); *P* = 5.75 × 10^−14^) which lies ~15 kb from rs2787486 and is correlated with it (r^2^ = 0.99). A regression model suggests that both rs2787486 (*P* = 0.02) and rs244353 (*P* = 0.11) are detecting the same risk association. To dissect further the observed associations all SNPs displaying evidence for association (*P* < 10^−4^ and MAF ≥ 0.02) with overall breast cancer risk (228 SNPs, Supplementary Table 1) in European studies were included in a forward stepwise regression model. This analysis identified a single association signal marked by top imputed variant rs2787486 (that is, no further SNPs were associated after adjustment for rs2787486). We also utilized penalized logistic regression models (based on the normal exponential gamma probability density) implemented in HyperLasso^13^, including all typed and imputed variants with an specified lambda of 0.05 and a penalty of 491 for overall risk (based on the sample size and a type I error of 0.001)^28^.

In this analysis, the best fitting model also included just one SNP, rs2787486.

On the assumption of a single causal variant, we calculated the likelihood ratio of each SNP relative to rs2787486 with respect to overall risk and SNPs with a relative likelihood ratio of <1:100 were excluded from further consideration^29^. After this exclusion process 28 SNPs (17 genotyped and 11 imputed), spanning 52.3 Kb (positions 53,176,211 to 53,228,543), remained as candidate causal variants (Table 1, Supplementary Table 2). These SNPs have very similar allele frequencies and are strongly correlated with SNP rs2787486. The two SNPs first reported to be associated with breast cancer were both excluded from this set of 28 candidate causal variants by likelihood ratio tests relative to SNP rs2787486 (likelihood ratios: 1:177439 for rs6504950 and 1: 3271 for rs1156287). Caswell *et al*.^30^ subsequently identified marker rs11658717 as a potential causal candidate, but this variant is ranked 49^th^ and has a likelihood ratio of 1: 3875 relative to lead SNP rs2787486 - hence this has also been ruled out as potential casual candidate by our analysis.

### Association with breast cancer subtypes

Based on data from European studies, 66 genotyped SNPs and 72 imputed SNPs were associated with risk of ER+ breast cancer (*P* values 10^−7^ to 10^−14^). The most strongly associated SNP for overall breast cancer (rs2787486) was also the most strongly associated for ER+ disease (OR = 0.91 (0.88–0.93), *P* = 1.39 × 10^−14^), but was more weakly associated with ER− disease (OR = 0.95 (0.91–0.99), *P* = 1.77 × 10^−02^, 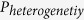 = 0.017). The most strongly associated SNP for ER− disease was c17_pos53079506 (OR = 1.19 (1.07–1.33), *P* = 0.0017, 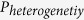 = 0.015) located ~130 kb from rs2787486.

To determine whether there were additional subtype-specific association signals, we included all SNPs displaying evidence for association with ER+ disease (345 SNPs, *P* < 10^−4^ and MAF ≥ 2%) in a separate forward stepwise regression model. The top associated SNP was rs2787486 (OR = 0.91 (0.88–0.93), *P* = 1.39 × 10^−14^) - the same SNP best-associated with overall risk and the same signal was localized by the HyperLasso search with penalty term set to 424. We calculated the likelihood ratio of each ER+ associated SNP (*r*^*2*^ > 0.6) relative to rs2787486 and retained a list of 37 markers (17 genotyped, 20 imputed) with a likelihood ratio of >1:100. This list included all 28 candidate causal SNPs for overall risk, except for imputed variant rs187242. No stepwise selection for ER- risk was performed as none of the markers fulfilled the inclusion criteria.

### Overall breast cancer and subtype risk association in Asian studies

Among Asian studies, the strongest association with overall breast cancer risk was observed for genotyped SNP rs244353 (OR = 0.91 (0.85–0.93), *P* = 2.57 × 10^−3^). This SNP was one of the candidate causal SNPs in Europeans, and conferred a similar OR in both populations (Supplementary Table 3). Of the genotyped markers 299 (among Europeans) and 38 (among Asians) exhibit a marginal P-value ≤ 0.05; of which 27 were significant in both populations and 9 (Supplementary Table 4) were selected as potential candidates by relative likelihood filtering in the European population. No evidence of heterogeneity in tumour subtype OR was observed for this SNP. The strongest association with ER+ disease was with SNP rs7503456 (OR = 0.89 (0.84–0.95), *P* = 2.73 × 10^−4^), which showed no association in the European studies (OR = 1.00 (0.97–1.03), *P* = 0.775). For ER− disease the strongest association was found with c17_pos52831447 (OR = 1.91 (1.25–2.92), *P* = 3.8 × 10^−3^), which showed no association among the European studies (OR = 1.04 (0.98–1.09), *P* = 0.181).

### Analyses of overlap between candidate causal variants and regulatory sites

**Figure 2:**
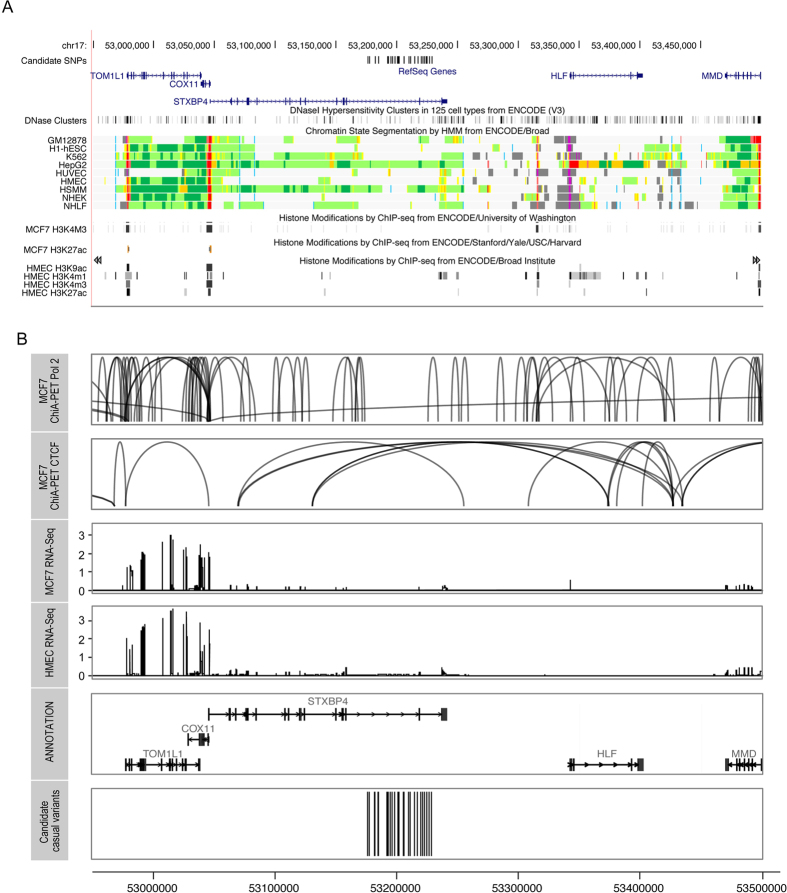
In silico analysis of the 17q22 locus. (**A**) Functional annotations using data from the ENCODE project. From top to bottom, epigenetic signals evaluated included DNase clusters, and ENCODE chromatin states (ChromHMM) and histone modifications in HMEC and MCF7 cell lines. The detailed colour scheme in chromatin states is described in the UCSC browser. All tracks were generated by the UCSC genome browser (hg 19). (**B**) Long-range chromatin interactions. From top to bottom, ChIA-PET data for Pol2 and CTCF in MCF7 cell lines. The ChIA-PET raw data available on GEO under the following accession (GSE63525.K56, GSE33664, GSE39495) were processed with the GenomicRanges package. -RNAseq data from MCF7 and HMEC cell lines. The value of the RNAseq analysis corresponds to the mean RPM value for *COX11, TOM1L1 and STXBP4* from 4 HMEC and 19 MCF7 datasets, respectively. The annotation has been obtained through the Bioconductor annotation package TxDb.Hsapiens.UCSC.hg19.knownGene. The tracks have been generated using ggplot2 and ggbio library in R.

The 28 candidate causal variants (Table 1, Supplementary Table 5) fall in a 53.2 kb region spanning two introns of *STXBP4* (Fig. 1). We mapped these to regulatory annotations from ENCODE. Analysis of DNase hypersensitivity clusters indicates that SNP rs244353 overlaps with a DHS in 23 cell lines, while rs2787481 and rs244317 show overlap in one and three cell lines, respectively. However, none of these overlaps were observed in mammary cells. None of the candidate causal SNPs overlapped with histone modification marks (H3K4me1, H3K4me3, H3K9ac, H3K27ac) in the mammary cells line HMEC and MCF7 breast cancer cells (Fig. 2A). We analysed enhancer-promoter interactions using Chromatin Interaction Analysis by Paired End Tag (ChiA-PET) data for CCCTC-binding factor (CTCF) and DNA polymerase II (Pol2) in MCF7 breast tumour derived cells. Although multiple chromosomal interactions were observed across the locus for both Pol2 and CTCF in MCF7 cells there was a notable dearth of such interactions in the region encompassing the strongest candidate causal variants (Fig. 2B). No interactions were observed in Hi-C data from HMEC cells in this region (data not shown).

**Figure 3:**
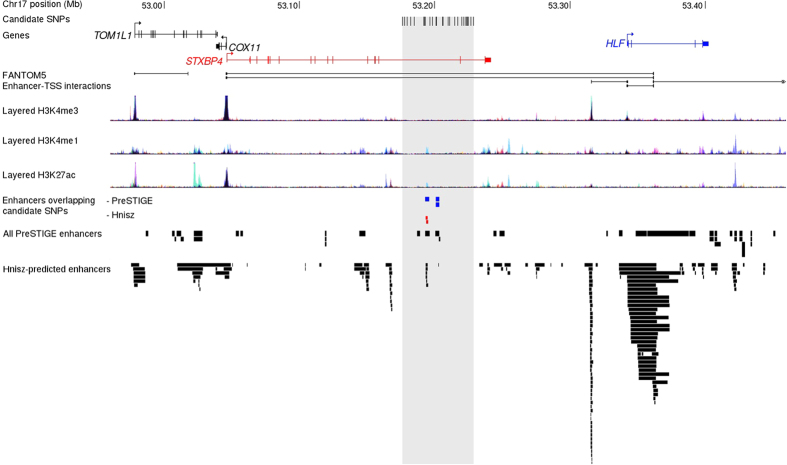
Functional annotation of the 17q22 locus. Positions of candidate causal variants are shown as black tick marks in relation to local genes. Epigenomic marks associated with enhancers are shown as described by FANTOM, ENCODE, PreSTIGE and Hnisz *et al*.^18^. Enhancers which overlap candidate SNPs are coded in color to match their predicted target gene. The grey shaded region area depicts the region bound by un-excluded variants.

Data from Hnisz *et al*.^18^ indicates the existence of several enhancers across the region, including a small one predicted to target the *STXBP4* gene (observed in both HUVEC and CD4 memory cells) that includes the candidate causal variant SNP rs244353 (Fig. 3). However, PreSTIGE^17^ indicates that an overlapping enhancer element (also containing rs244353) active in HepG2 cells may target the *HLF* gene. Another PreSTIGE element containing rs244336 and rs244337 is predicted to target *HLF* in colon crypt cells (Fig. 3).

### Local Gene Expression analyses

ENCODE RNA-seq data show that *COX11* and *TOM1L1* are highly expressed in both MCF7 and HMEC cells lines while *STXBP4* shows much lower expression levels (Fig. 2B). We performed allele specific expression (ASE) analysis using RNAseq and SNP array genotype data from TCGA^19^. Allelic imbalance at marginal statistical significance (P =0.032) in *COX11* expression was detected with the alleles of candidate causal SNP rs2787481 (r^2^ = 0.90 with rs2787486 ~1.3 Kb away) but not with any other genes within 1 Mb (*TOM1L1, STXBP4, HLF, MMD, PCTP*) using the same SNP (Supplementary Figure 3, Supplementary Table 6).

We also examined the associations of SNPs with the expression levels of the same local genes. In the normal tissue samples (n=135) from the METABRIC study the top breast cancer candidate causal variant was also associated with *COX11* expression levels (Supplementary Table 7). The most significant breast cancer associated SNP, rs2787486, was associated with differential expression of *COX11* (*P* = 0.00019, FDR corrected *P* = 0.05) but not significantly associated with expression of any other genes after FDR correction. However, other SNPs across this region were more significantly associated with *COX11* expression (strongest association with SNP rs138326143, *P* = 1.4 × 10^−7^, FDR corrected *P* = 0.003,^31^) suggesting that the observed change in *COX11* expression in normal breast tissue is unlikely to be the main driver of breast cancer risk. By contrast, no associations with *COX11* expression were observed in the TCGA breast tumour samples with the top breast cancer risk SNPs (Supplementary Figure 5). However, in TCGA multiple SNPs associate with expression of the shortest isoform of *STXBP4* (uc010dcc) with the top breast cancer risk SNP, rs2787486 having a FDR corrected *P* = 4.0 × 10^−8^ (r^2^ = 0.06, Supplementary Figure 4). Other SNPs, including rs244317 and rs11658717 displayed more significant associations with expression of this isoform (FDR corrected *P* = 3.8 × 10^−9^, r^2^ = 0.07, Supplementary Figure 4) than the top breast cancer risk SNP. The minor alleles are associated with increased expression of isoform uc010dcc, and explain 7% of the variation in its expression levels. Of note Caswell *et al*.^30^ reported that the A-G base change of SNP rs11658717 mediates the use of different splice junction between exons 5 and 6 of the STXBP4 gene and thus generates the shorter uc010dcc isoform. Our expression data thus support this report but our association evidence (likelihood ratio 1:3875 relative to lead SNP rs2787486) indicates that this SNP is unlikely to be a causal variant driving breast cancer risk.

We also interrogated candidate variants in the v6 data release from the Gene-Tissue Expression (GTEx) project^32^. We found a significant association between the minor allele of SNP rs244353 and decreased expression of their measured *STXBP4* (full length) isoform in multiple tissues including breast (n = 183; *P* = 1.3 × 10^−6^; Supplementary Figure 6, Supplementary Table 8). These different METABRIC, TCGA and GTEX findings appear contradictory of each other: SNPs rs244353 and rs244317 are highly correlated (r^2^ = 0.90 and yet their minor alleles are significantly associated with decreased *STXBP4* expression in GTEx but increased expression of isoform (uc010dcc) in TCGA. One possible explanation is that the *STXBP4* full length transcript (measured in GTEx) and the short transcript (uc010dcc, measured in TCGA) are regulated by different mechanisms^33^.

## Discussion

In this - study, using more than 100,000 cases and controls of European and Asian ancestry participating in BCAC, we have confirmed previous reports of associations of SNPs in the 17q22 region with risk of breast cancer^3^,^4^. Moreover, we identified a set of 28 strong candidate causal variants, of which one or more is the likely driver of these reported associations. Of these, SNP rs2787486, which is correlated with previously reported candidates: rs6504950 (r^2^ = 0.73), rs1156287 (r^2^ = 0.83) and rs11658717 (r^2^ = 0.84)^5^,^30^; was the most strongly associated variant with overall risk (OR = 0.92 (95% CI: 0.90–0.94), *P* = 8.96 × 10^−15^). A similar magnitude of association was observed in both European and Asian women, consistent with the same causal variant mediating risk in both populations. The association was stronger for ER+ than ER− breast cancer.

All the remaining candidate causal variants lie in a 53 Kb region (positions 52,176,211 to 53,228,543) spanning two introns of the *STXBP4* gene. None are predicted to alter the coding sequence of this gene and so it is most likely that the association is mediated through altering the regulation of one or more nearby genes. CHIA-Pet studies in the breast cancer MCF7 cell line reveal many chromatin interactions across the wider region (Fig. 2); however, there is a dearth of such interactions in the region encompassing the strongest candidate causal variants. Furthermore, in MCF7 or HMEC mammary cell lines there was no evidence of histone modification or open chromatin, indicative of the existence of regulatory regions, overlapping the best candidate causal variants, although such regions do exist in the wider region studied (Fig. 3). In this respect, this association signal differs from other breast cancer association signals in which strong evidence of regulatory elements in mammary cell lines has been observed^34^. An enhancer is predicted by FANTOM in many cell types while data from Hnisz *et al*.^18^ (Fig. 3) indicates the existence of a small enhancer region, targeting *STXBP4* (observed in both HUVEC and CD4 memory cells) that overlaps with candidate causal variant rs244353. However, PreSTIGE data indicate that nearby enhancer elements may target *HLF* in HepG2 and colonic crypt cells (Fig. 3).

Of the candidate genes in the region, both *COX11* and *TOM1L1* are highly expressed in both the HMEC and MCF7 breast cancer cell lines, while *STXBP4* shows much lower expression (detected by RNAseq in TCGA). In support of *COX11* and *TOM1L1* being the targets of this breast cancer susceptibility locus, eQTL analyses in normal breast tissue showed borderline significant associations of the risk alleles of top candidate causal SNP rs2787486 with increased expression levels of both *TOM1L1* and *COX11*; candidate SNP rs2787481 also showed evidence of allelic imbalance in *COX11* expression. *COX11* encodes a cytochrome c oxidase copper chaperone – a nuclear-encoded protein component of a mitochondrial-membrane-embedded respiratory complex and *TOM1L1* encodes a Target of myb1-like1 membrane trafficking protein^35^. Both genes are expressed in the majority of tissues examined in the Human Protein Atlas^32^. eQTL analysis in breast tumour tissues in TCGA find the risk allele of top candidate breast cancer risk SNP, rs2787486, to be significantly associated with increased *STXBP4* expression, but not with *COX11* (Supplementary Figures 4 and 5).

Furthermore, Hnisz *et al*.^18^ indicates the presence of an enhancer element that overlaps with candidate causal SNP rs244353 and potentially targets *STXBP4* (observed in both HUVEC and CD4 memory cells). Consistent with this, TCGA eQTL studies in breast tumour tissues find the risk allele of top candidate breast cancer risk SNP, rs2787486, to be significantly associated with increased *STXBP4* mRNA expression. The *STXBP4* gene encodes Syntaxin binding protein 4, a scaffold protein, which has been shown to stabilise and prevent degradation of an isoform of p63^36^. P63 is, in turn, a member of the p53 tumour suppressor protein family and thus possibly a biologically more plausible candidate cancer gene than *COX11* or *TOM1L1*.

We conclude that one or more of the 28 variants we identified is causally related to breast cancer risk, most likely through regulation of *STXBP4*, *COX11* and *TOM1L1*, with the balance of the evidence favouring *STXBP4* as the most important target. It remains possible, however, that the target gene(s) is more distant (>1 Mb) from the associated variants and so have not yet been considered. Further functional analyses will be required to determine the mechanism underlying this association and the downstream targets.

## Additional Information

**How to cite this article**: Darabi, H. *et al*. Fine scale mapping of the 17q22 breast cancer locus using dense SNPs, genotyped within the Collaborative Oncological Gene-Environment Study (COGs). *Sci. Rep.* **6**, 32512; doi: 10.1038/srep32512 (2016).

## Supplementary Material

Supplementary Information

## Figures and Tables

**Table 1:** Association result of independent signal among European and the set of highly correlated common variants.

Population		European	Candidate Causal SNPs	Position^b^	r^2^^c^	RL^c^	Info^d^	P-value^e^	OR (95% CI)^e^
			rs2787497^**§**^	53176211	0.89	0.01	1	9.27E-13	0.93 (0.91, 0.95)
**Independent Signal**		rs2787486*	rs8082622^**§**^	53177567	0.90	0.01	1	7.06E-13	0.93 (0.91, 0.94)
			rs2529510^**§§**^	53182046	0.84	0.13	0.91	6.92E-14	0.92 (0.90, 0.94)
**Major/Minor (MAF)**		A/C (0.28)	rs244373^**§§**^	53184949	0.99	0.13	0.98	7.30E-14	0.92 (0.90, 0.94)
			rs244358^**§**^	53192231	0.89	0.01	1	9.68E-13	0.93 (0.91, 0.95)
**Overall Risk** a	**P-value**	8.96E-15	rs187242^**§§**^	53192946	0.98	0.01	0.98	8.17E-13	0.93 (0.91, 0.95)
	**OR (95% CI)**	0.92 (0.90, 0.94)	rs244353^**§**^	53194769	0.99	0.16	1	5.75E-14	0.92 (0.90, 0.94)
			rs244348^**§**^	53196291	0.90	0.01	1	7.08E-13	0.93 (0.91, 0.94)
**ER+** a	**P-value**	1.39E-14	rs244342^**§**^	53198407	0.99	0.14	1	6.73E-14	0.92 (0.90, 0.94)
	**OR (95% CI)**	0.91 (0.88, 0.93)	rs244338^**§**^	53200418	0.99	0.15	1	6.21E-14	0.92 (0.90, 0.94)
			rs244337^**§§**^	53201124	0.99	0.14	0.99	6.60E-14	0.92 (0.90, 0.94)
**ER−** a	**P-value**	0.0177	rs244336^**§**^	53201502	0.90	0.01	1	8.80E-13	0.93 (0.91, 0.95)
	**OR (95% CI)**	0.95 (0.91, 0.99)	chr17:53205761:D^**§§**^	53205761	0.90	0.01	0.99	9.42E-13	0.93 (0.91, 0.95)
			rs2628321^**§**^	53205917	0.99	0.16	1	5.88E-14	0.92 (0.90, 0.94)
			rs2787486^§§^*	53209774			0.96	8.96E-15	0.92 (0.90, 0.94)
			rs2787481^**§**^	53211110	0.90	0.01	1	6.44E-13	0.92 (0.91, 0.94)
			rs244315^**§**^	53214654	0.90	0.02	1	4.72E-13	0.92 (0.91, 0.94)
			rs244317^**§**^	53216985	0.90	0.02	1	5.79E-13	0.92 (0.91, 0.94)
			rs2628316^**§**^	53219837	0.99	0.15	1	6.00E-14	0.92 (0.90, 0.94)
			chr17:53221365:I^**§§**^	53221365	0.99	0.10	0.98	9.68E-14	0.92 (0.90, 0.94)
			rs244318^**§§**^	53221368	0.97	0.02	0.98	4.19E-13	0.93 (0.91, 0.95)
			rs244319^**§**^	53222874	0.98	0.04	1	2.19E-13	0.93 (0.91, 0.94)
			rs244320^**§**^	53222920	0.98	0.04	1	2.19E-13	0.93 (0.91, 0.94)
			rs244321^**§§**^	53223998	0.98	0.05	0.99	1.72E-13	0.92 (0.91, 0.94)
			rs10432032^**§**^	53224088	0.98	0.04	1	2.47E-13	0.93 (0.91, 0.94)
			rs244322^**§§**^	53224759	0.98	0.05	0.99	2.03E-13	0.93 (0.91, 0.94)
			rs2628315^**§**^	53226622	0.99	0.10	1	9.02E-14	0.92 (0.90, 0.94)
			rs244294^**§§**^	53228543	0.98	0.01	0.99	9.28E-13	0.93 (0.91, 0.95)

^a^P-value, Odds ratio (OR) and 95% Confidence Interval (CI) for association with overall breast cancer risk, Estrogen Positive (ER+) and Estrogen Negative (ER−) disease.

^b^Build 37 coordinates on chromosome 17.

^c^Correlation (r^2^) and Relative Likelihood ratio (RL) with respect to lead SNP rs2787486

^d^IMPUTE2 info score.

^e^P-value, Odds ratio (OR) and 95% Confidence Interval (CI) for association with overall breast cancer risk.

^§/§§^Genotyped/Imputed marker.

^*^Lead SNP.
