# Supplementary material for: Fine scale mapping of the 17q22 breast cancer locus using dense SNPs, genotyped within the Collaborative Oncological Gene-Environment Study (COGs)
Source: Sci Rep. 2016 Sep 7;6:32512. doi: 10.1038/srep32512 (PMC5013272; doi:10.1038/srep32512)
Supplement: Supplementary Information [file srep32512-s1.pdf]

Fine scale mapping of the 17q22 breast cancer locus using dense SNPs, genotyped within the Collaborative Oncological Gene-Environment Study (COGs)

Hatef Darabi [1*], Jonathan Beesley [2§], Arnaud Droit [3§], Siddhartha Kar [4§], Silje Nord [5§], Mahdi Moradi Marjaneh [2§], Penny Soucy [6], Kyriaki Michailidou [7,8], Maya Ghoussaini [4], Hanna Fues Wahl [1], Manjeet K. Bolla [7], Qin Wang [7], Joe Dennis [7], M. Rosario Alonso [9], Irene L. Andrulis [10,11], Hoda Anton-Culver [12], Volker Arndt [13], Matthias W. Beckmann [14], Javier Benitez [15,16], Natalia V. Bogdanova [17], Stig E. Bojesen [18-20], Hiltrud Brauch [21-23], Hermann Brenner [13,23,24], Annegien Broeks [25], Thomas Brüning [26], Barbara Burwinkel [27,28], Jenny Chang-Claude [29,30], Ji-Yeob Choi [31,32], Don M. Conroy [4], Fergus J. Couch [33], Angela Cox [34], Simon S. Cross [35], Kamila Czene [1], Peter Devilee [36,37], Thilo Dörk [38], Douglas F. Easton [4,7], Peter A. Fasching [14,39], Jonine Figueroa [40,41], Olivia Fletcher [42,43], Henrik Flyger [44], Eva Galle [45,46], Montserrat García-Closas [41], Graham G. Giles [47,48], Mark S. Goldberg [49,50], Anna González-Neira [15], Pascal Guénel [51], Christopher A. Haiman [52], Emily Hallberg [53], Ute Hamann [54], Mikael Hartman [55,56], Antoinette Hollestelle [57], John L. Hopper [48], Hidemi Ito [58], Anna Jakubowska [59], Nichola Johnson [42,43], Daehee Kang [31,32,60], Sofia Khan [61], Veli-Matti Kosma [62-64], Mieke Kriege [57], Vessela Kristensen [5,65,66], Diether Lambrechts [45,46], Loic Le Marchand [67], Soo Chin Lee [68,69], Annika Lindblom [70], Artitaya Lophatananon [71], Jan Lubinski [59], Arto Mannermaa [62-64], Siranoush Manoukian [72], Sara Margolin [73], Keitaro Matsuo [74], Rebecca Mayes [4], James McKay [75], Alfons Meindl [76], Roger L. Milne [47,48], Kenneth Muir [71,77], Susan L. Neuhausen [78], Heli Nevanlinna [61], Curtis Olswold [53], Nick Orr [42], Paolo Peterlongo [79], Guillermo Pita [9], Katri Pylkäs [80,81], Anja Rudolph [29], Suleeporn Sangrajrang [82], Elinor J. Sawyer [83], Marjanka K. Schmidt [25], Rita K. Schmutzler [84-86], Caroline Seynaeve [57], Mitul Shah [4], Chen-Yang Shen [87,88], Xiao-Ou Shu [89], Melissa C. Southey [90], Daniel O. Stram [52], Harald Surowy [27,28], Anthony Swerdlow [43,91], Soo H. Teo [92,93], Daniel C. Tessier [94], Ian Tomlinson [95], Diana Torres [54,96], Thérèse Truong [51], Celine M. Vachon [53], Daniel

Vincent [94], Robert Winqvist [80,81], Anna H. Wu [52], Pei-Ei Wu [88], Cheng Har Yip [93], Wei Zheng [89], Paul D. P. Pharoah [4,7], Per Hall [1], Stacey L. Edwards [2], Jacques Simard [6], Juliet D. French [2], Georgia Chenevix-Trench [2], Alison M. Dunning [4]

§ Contributed equally.

1 Department of Medical Epidemiology and Biostatistics, Karolinska Institutet, Stockholm, Sweden.
2 Department of Genetics, QIMR Berghofer Medical Research Institute, Brisbane, Australia.
3 Département de Médecine Moléculaire, Faculté de Médecine, Centre Hospitalier Universitaire de Québec Research Center, Laval University, Québec City, Canada.
4 Centre for Cancer Genetic Epidemiology, Department of Oncology, University of Cambridge, Cambridge, UK.
5 Department of Cancer Genetics, Institute for Cancer Research, Oslo University Hospital Radiumhospitalet, Oslo, Norway.
6 Genomics Center, Centre Hospitalier Universitaire de Québec Research Center, Laval University, Québec City, Canada.
7 Centre for Cancer Genetic Epidemiology, Department of Public Health and Primary Care, University of Cambridge, Cambridge, UK.
8 Department of Electron Microscopy/Molecular Pathology, The Cyprus Institute of Neurology and Genetics, Nicosia, Cyprus.
9 Human Genotyping-CEGEN Unit, Human Cancer Genetic Program, Spanish National Cancer Research Centre, Madrid, Spain.
10 Lunenfeld-Tanenbaum Research Institute of Mount Sinai Hospital, Toronto, Canada.
11 Department of Molecular Genetics, University of Toronto, Toronto, Canada.
12 Department of Epidemiology, University of California Irvine, Irvine, CA, USA.
13 Division of Clinical Epidemiology and Aging Research, German Cancer Research Center (DKFZ), Heidelberg, Germany.
14 Department of Gynaecology and Obstetrics, University Hospital Erlangen, Friedrich-Alexander University Erlangen-Nuremberg, Comprehensive Cancer Center Erlangen-EMN, Erlangen, Germany.
15 Human Cancer Genetics Program, Spanish National Cancer Research Centre, Madrid, Spain.
16 Centro de Investigación en Red de Enfermedades Raras, Valencia, Spain.
17 Department of Radiation Oncology, Hannover Medical School, Hannover, Germany.
18 Copenhagen General Population Study, Herlev and Gentofte Hospital, Copenhagen University Hospital, Herlev, Denmark.
19 Department of Clinical Biochemistry, Herlev and Gentofte Hospital, Copenhagen University Hospital, Herlev, Denmark.
20 Faculty of Health and Medical Sciences, University of Copenhagen, Copenhagen, Denmark.
21 Dr. Margarete Fischer-Bosch-Institute of Clinical Pharmacology, Stuttgart, Germany.
22 University of Tübingen, Tübingen, Germany.
23 German Cancer Consortium (DKTK), German Cancer Research Center (DKFZ), Heidelberg, Germany.
24 Division of Preventive Oncology, German Cancer Research Center (DKFZ) and National Center for Tumor Diseases (NCT), Heidelberg, Germany.
25 Netherlands Cancer Institute, Antoni van Leeuwenhoek hospital, Amsterdam, The Netherlands.

26 Institute for Prevention and Occupational Medicine of the German Social Accident Insurance, Institute of the Ruhr University Bochum, Bochum, Germany.
27 Department of Obstetrics and Gynecology, University of Heidelberg, Heidelberg, Germany.
28 Molecular Epidemiology Group, German Cancer Research Center (DKFZ), Heidelberg, Germany.
29 Division of Cancer Epidemiology, German Cancer Research Center (DKFZ), Heidelberg, Germany.
30 University Cancer Center Hamburg (UCCH), University Medical Center Hamburg-Eppendorf, Hamburg, Germany.
31 Department of Biomedical Sciences, Seoul National University College of Medicine, Seoul, Korea.
32 Cancer Research Institute, Seoul National University, Seoul, Korea.
33 Department of Laboratory Medicine and Pathology, Mayo Clinic, Rochester, MN, USA.
34 Sheffield Cancer Research, Department of Oncology and Metabolism, University of Sheffield, Sheffield, UK.
35 Academic Unit of Pathology, Department of Neuroscience, University of Sheffield, Sheffield, UK.
36 Department of Pathology, Leiden University Medical Center, Leiden, The Netherlands.
37 Department of Human Genetics, Leiden University Medical Center, Leiden, The Netherlands.
38 Gynaecology Research Unit, Hannover Medical School, Hannover, Germany.
39 David Geffen School of Medicine, Department of Medicine Division of Hematology and Oncology, University of California at Los Angeles, Los Angeles, CA, USA.
40 Usher Institute of Population Health Sciences and Informatics, The University of Edinburgh Medical School, Edinburgh, UK.
41 Division of Cancer Epidemiology and Genetics, National Cancer Institute, Rockville, MD, USA.
42 Breakthrough Breast Cancer Research Centre, The Institute of Cancer Research, London, UK.
43 Division of Breast Cancer Research, The Institute of Cancer Research, London, UK.
44 Department of Breast Surgery, Herlev and Gentofte Hospital, Copenhagen University Hospital, Herlev, Denmark.
45 Vesalius Research Center, Leuven, Belgium.
46 Laboratory for Translational Genetics, Department of Oncology, University of Leuven, Leuven, Belgium.
47 Cancer Epidemiology Centre, Cancer Council Victoria, Melbourne, Australia.
48 Centre for Epidemiology and Biostatistics, Melbourne School of Population and Global health, The University of Melbourne, Melbourne, Australia.
49 Department of Medicine, McGill University, Montreal, Canada.
50 Division of Clinical Epidemiology, Royal Victoria Hospital, McGill University, Montreal, Canada.
51 Cancer & Environment Group, Center for Research in Epidemiology and Population Health (CESP), INSERM, University Paris-Sud, University Paris-Saclay, Villejuif, France.
52 Department of Preventive Medicine, Keck School of Medicine, University of Southern California, Los Angeles, CA, USA.
53 Department of Health Sciences Research, Mayo Clinic, Rochester, MN, USA.
54 Molecular Genetics of Breast Cancer, German Cancer Research Center (DKFZ), Heidelberg, Germany.
55 Saw Swee Hock School of Public Health, National University of Singapore, Singapore, Singapore.
56 Department of Surgery, National University Health System, Singapore, Singapore.

57 Department of Medical Oncology, Family Cancer Clinic, Erasmus MC Cancer Institute, Rotterdam, The Netherlands.
58 Division of Epidemiology and Prevention, Aichi Cancer Center Research Institute, Nagoya, Japan.
59 Department of Genetics and Pathology, Pomeranian Medical University, Szczecin, Poland.
60 Department of Preventive Medicine, Seoul National University College of Medicine, Seoul, Korea.
61 Department of Obstetrics and Gynecology, Helsinki University Hospital, University of Helsinki, Helsinki, Finland.
62 Cancer Center of Eastern Finland, University of Eastern Finland, Kuopio, Finland.
63 Institute of Clinical Medicine, Pathology and Forensic Medicine, University of Eastern Finland, Kuopio, Finland.
64 Imaging Center, Department of Clinical Pathology, Kuopio University Hospital, Kuopio, Finland.
65 K.G. Jebsen Center for Breast Cancer Research, Institute of Clinical Medicine, Faculty of Medicine, University of Oslo, Oslo, Norway.
66 Department of Clinical Molecular Biology, Oslo University Hospital, University of Oslo, Oslo, Norway.
67 University of Hawaii Cancer Center, Honolulu, HI, USA.
68 Department of Hematology-Oncology, National University Health System, Singapore, Singapore.
69 Cancer Science Institute of Singapore, National University of Singapore, Singapore, Singapore.
70 Department of Molecular Medicine and Surgery, Karolinska Institutet, Stockholm, Sweden.
71 Division of Health Sciences, Warwick Medical School, Warwick University, Coventry, UK.
72 Unit of Molecular Bases of Genetic Risk and Genetic Testing, Department of Preventive and Predictive Medicine, Fondazione IRCCS (Istituto Di Ricovero e Cura a Carattere Scientifico) Istituto Nazionale dei Tumori (INT), Milan, Italy.
73 Department of Oncology - Pathology, Karolinska Institutet, Stockholm, Sweden.
74 Division of Molecular Medicine, Aichi Cancer Center Research Institute, Nagoya, Japan.
75 International Agency for Research on Cancer, Lyon, France.
76 Division of Gynaecology and Obstetrics, Technische Universität München, Munich, Germany.
77 Institute of Population Health, University of Manchester, Manchester, UK.
78 Department of Population Sciences, Beckman Research Institute of City of Hope, Duarte, CA, USA.
79 IFOM, The FIRC (Italian Foundation for Cancer Research) Institute of Molecular Oncology, Milan, Italy.
80 Laboratory of Cancer Genetics and Tumor Biology, Cancer and Translational Medicine Research Unit, Biocenter Oulu, University of Oulu, Oulu, Finland.
81 Laboratory of Cancer Genetics and Tumor Biology, Northern Finland Laboratory Centre Oulu, Oulu, Finland.
82 National Cancer Institute, Bangkok, Thailand.
83 Research Oncology, Guy's Hospital, King's College London, London, UK.
84 Center for Hereditary Breast and Ovarian Cancer, University Hospital of Cologne, Cologne, Germany.
85 Center for Integrated Oncology (CIO), University Hospital of Cologne, Cologne, Germany.

86 Center for Molecular Medicine Cologne (CMMC), University of Cologne, Cologne, Germany.
87 Institute of Biomedical Sciences, Academia Sinica, Taipei, Taiwan.
88 Taiwan Biobank, Institute of Biomedical Sciences, Academia Sinica, Taipei, Taiwan.
89 Division of Epidemiology, Department of Medicine, Vanderbilt-Ingram Cancer Center, Vanderbilt University School of Medicine, Nashville, TN, USA.
90 Department of Pathology, The University of Melbourne, Melbourne, Australia.
91 Division of Genetics and Epidemiology, The Institute of Cancer Research, London, UK.
92 Cancer Research Initiatives Foundation, Subang Jaya, Selangor, Malaysia.
93 Breast Cancer Research Unit, Cancer Research Institute, University Malaya Medical Centre, Kuala Lumpur, Malaysia.
94 McGill University and Génome Québec Innovation Centre, Montréal, Canada.
95 Wellcome Trust Centre for Human Genetics and Oxford NIHR Biomedical Research Centre, University of Oxford, Oxford, UK.
96 Institute of Human Genetics, Pontificia Universidad Javeriana, Bogota, Colombia.

Supplementary Table 1: Based on data from all European studies, genotyped or imputed variants associated with overall risk (P-values < $1x10^{-4}$).

						Overall Risk [e]		ER+ [e]		ER- [e]		Heterogeneity [f]	
SNP	Position [a]	Type	Major/Minor [b]	MAF [c]	Info [d]	P-value	OR (95% CI)	P-value	OR (95% CI)	P-value	OR (95% CI)	P-value	RL [g]
rs2787486	53209774	Imputed	A/C	0.28	0.958	8.96E-15	0.92 (0.9,0.94)	1.39E-14	0.91 (0.88,0.93)	0.0177	0.95 (0.91,0.99)	0.016634	-
rs244353	53194769	Genotyped	G/A	0.29	1	5.75E-14	0.92 (0.9,0.94)	5.96E-14	0.91 (0.89,0.93)	0.0275	0.96 (0.92,1)	0.015142	0.161
rs2628321	53205917	Genotyped	A/G	0.29	1	5.88E-14	0.92 (0.9,0.94)	7.01E-14	0.91 (0.89,0.93)	0.0277	0.96 (0.92,1)	0.015346	0.157
rs2628316	53219837	Genotyped	A/G	0.29	1	6.00E-14	0.92 (0.9,0.94)	1.17E-13	0.91 (0.89,0.93)	0.0276	0.96 (0.92,1)	0.015608	0.154
rs244338	53200418	Genotyped	G/A	0.29	1	6.21E-14	0.92 (0.9,0.94)	6.70E-14	0.91 (0.89,0.93)	0.0276	0.96 (0.92,1)	0.015585	0.149
rs244337	53201124	Imputed	G/T	0.29	0.992	6.60E-14	0.92 (0.9,0.94)	6.29E-14	0.91 (0.89,0.93)	0.0304	0.96 (0.92,1)	0.013809	0.14
rs244342	53198407	Genotyped	C/T	0.29	1	6.73E-14	0.92 (0.9,0.94)	7.48E-14	0.91 (0.89,0.93)	0.03	0.96 (0.92,1)	0.014533	0.138
rs2529510	53182046	Imputed	T/A	0.3	0.91	6.92E-14	0.92 (0.9,0.94)	2.08E-14	0.91 (0.88,0.93)	0.0645	0.96 (0.92,1)	0.00655	0.134
rs244373	53184949	Imputed	C/T	0.29	0.984	7.30E-14	0.92 (0.9,0.94)	7.84E-14	0.91 (0.89,0.93)	0.0315	0.96 (0.92,1)	0.012324	0.127
rs2628315	53226622	Genotyped	G/A	0.29	1	9.02E-14	0.92 (0.9,0.94)	1.78E-13	0.91 (0.89,0.93)	0.0259	0.96 (0.92,0.99)	0.018409	0.103
chr17:53221365:I	53221365	Imputed	T/TGC	0.29	0.978	9.68E-14	0.92 (0.9,0.94)	1.44E-13	0.91 (0.89,0.93)	0.0254	0.95 (0.92,0.99)	0.018015	0.096
rs244321	53223998	Imputed	T/A	0.29	0.989	1.72E-13	0.92 (0.91,0.94)	3.22E-13	0.91 (0.89,0.94)	0.0245	0.95 (0.92,0.99)	0.023115	0.055
rs244322	53224759	Imputed	C/T	0.29	0.99	2.03E-13	0.93 (0.91,0.94)	3.35E-13	0.91 (0.89,0.94)	0.0281	0.96 (0.92,1)	0.021021	0.046
rs244319	53222874	Genotyped	G/A	0.29	1	2.19E-13	0.93 (0.91,0.94)	4.18E-13	0.91 (0.89,0.94)	0.028	0.96 (0.92,1)	0.021933	0.043
rs244320	53222920	Genotyped	C/T	0.29	1	2.19E-13	0.93 (0.91,0.94)	4.18E-13	0.91 (0.89,0.94)	0.028	0.96 (0.92,1)	0.021933	0.043
rs10432032	53224088	Genotyped	A/G	0.29	1	2.47E-13	0.93 (0.91,0.94)	5.33E-13	0.91 (0.89,0.94)	0.0281	0.96 (0.92,1)	0.023436	0.038
rs244318	53221368	Imputed	A/G	0.29	0.985	4.19E-13	0.93 (0.91,0.95)	3.56E-13	0.91 (0.89,0.94)	0.036	0.96 (0.92,1)	0.014627	0.023
rs244315	53214654	Genotyped	C/T	0.27	1	4.72E-13	0.92 (0.91,0.94)	4.27E-13	0.91 (0.89,0.93)	0.0493	0.96 (0.92,1)	0.010962	0.02
rs244317	53216985	Genotyped	A/T	0.27	1	5.79E-13	0.92 (0.91,0.94)	2.74E-13	0.91 (0.89,0.93)	0.0649	0.96 (0.92,1)	0.007933	0.017

rs2787481	53211110	Genotyped	T/C	0.27	1	6.44E-13	0.92 (0.91,0.94)	2.93E-13	0.91 (0.89,0.93)	0.0637	0.96 (0.92,1)	0.008094	0.015
rs8082622	53177567	Genotyped	C/T	0.27	1	7.06E-13	0.93 (0.91,0.94)	6.24E-13	0.91 (0.89,0.94)	0.0528	0.96 (0.92,1)	0.011023	0.014
rs244348	53196291	Genotyped	G/A	0.27	1	7.08E-13	0.93 (0.91,0.94)	5.20E-13	0.91 (0.89,0.93)	0.0547	0.96 (0.92,1)	0.010577	0.014
rs187242	53192946	Imputed	T/A	0.29	0.984	8.17E-13	0.93 (0.91,0.95)	2.65E-12	0.92 (0.89,0.94)	0.0278	0.96 (0.92,1)	0.029991	0.012
rs244336	53201502	Genotyped	G/A	0.27	1	8.80E-13	0.93 (0.91,0.95)	7.60E-13	0.91 (0.89,0.94)	0.047	0.96 (0.92,1)	0.012981	0.011
rs2787497	53176211	Genotyped	A/G	0.27	1	9.27E-13	0.93 (0.91,0.95)	3.52E-13	0.91 (0.89,0.93)	0.0702	0.96 (0.92,1)	0.007276	0.01
rs244294	53228543	Imputed	A/G	0.29	0.986	9.28E-13	0.93 (0.91,0.95)	1.21E-12	0.91 (0.89,0.94)	0.0354	0.96 (0.92,1)	0.021678	0.01
chr17:53205761:D	53205761	Imputed	AT/A	0.27	0.987	9.42E-13	0.93 (0.91,0.95)	7.95E-13	0.91 (0.89,0.94)	0.0552	0.96 (0.92,1)	0.011595	0.01
rs244358	53192231	Genotyped	C/T	0.27	1	9.68E-13	0.93 (0.91,0.95)	2.97E-13	0.91 (0.89,0.93)	0.0893	0.97 (0.93,1.01)	0.005256	0.01
rs2787483	53210314	Imputed	G/A	0.27	0.982	1.09E-12	0.93 (0.91,0.95)	5.89E-13	0.91 (0.89,0.93)	0.0693	0.96 (0.92,1)	0.009154	0.009
rs2529504	53206164	Imputed	C/T	0.27	0.988	1.10E-12	0.93 (0.91,0.95)	8.72E-13	0.91 (0.89,0.94)	0.058	0.96 (0.92,1)	0.010945	0.009
rs2628317	53212573	Imputed	A/G	0.26	0.953	1.14E-12	0.92 (0.9,0.94)	5.42E-13	0.91 (0.89,0.93)	0.068	0.96 (0.92,1)	0.009292	0.009
rs244377	53183079	Imputed	T/C	0.27	0.987	1.31E-12	0.93 (0.91,0.95)	9.88E-13	0.91 (0.89,0.94)	0.0634	0.96 (0.92,1)	0.009435	0.007
chr17:53179335:I	53179335	Imputed	ACT/A	0.27	0.983	1.54E-12	0.93 (0.91,0.95)	6.19E-13	0.91 (0.89,0.93)	0.0805	0.96 (0.92,1)	0.007396	0.006
rs244376	53183199	Imputed	C/A	0.27	0.985	1.56E-12	0.93 (0.91,0.95)	6.75E-13	0.91 (0.89,0.93)	0.0788	0.96 (0.92,1)	0.007441	0.006
chr17:53179336:I	53179336	Imputed	CTT/C	0.27	0.983	1.60E-12	0.93 (0.91,0.95)	5.85E-13	0.91 (0.89,0.93)	0.0822	0.96 (0.92,1)	0.007065	0.006
rs181727	53190995	Imputed	T/C	0.27	0.986	1.63E-12	0.93 (0.91,0.95)	7.51E-13	0.91 (0.89,0.94)	0.0764	0.96 (0.92,1)	0.008189	0.006
chr17:53210314:I	53210314	Imputed	G/GTA	0.27	0.978	1.78E-12	0.93 (0.91,0.95)	8.50E-13	0.91 (0.89,0.93)	0.0742	0.96 (0.92,1)	0.008923	0.006
rs244379	53182578	Imputed	G/A	0.35	0.804	2.01E-12	0.92 (0.9,0.94)	4.41E-12	0.91 (0.89,0.94)	0.0329	0.95 (0.91,1)	0.067268	0.005
rs2787501	53093965	Imputed	C/G	0.3	0.989	2.91E-12	0.93 (0.91,0.95)	2.22E-12	0.92 (0.89,0.94)	0.0795	0.96 (0.93,1)	0.01109	0.003
rs12449538	53111121	Genotyped	C/T	0.3	1	3.56E-12	0.93 (0.91,0.95)	4.05E-12	0.92 (0.9,0.94)	0.0835	0.97 (0.93,1)	0.010763	0.003
rs2787500	53169806	Genotyped	T/G	0.3	1	4.41E-12	0.93 (0.91,0.95)	4.36E-12	0.92 (0.9,0.94)	0.0788	0.96 (0.93,1)	0.01295	0.002

rs4794551	53075427	Imputed	G/A	0.3	0.986	4.79E-12	0.93 (0.91,0.95)	2.06E-12	0.92 (0.89,0.94)	0.0839	0.97 (0.93,1)	0.009085	0.002
rs1812715	53174966	Imputed	G/A	0.32	0.942	5.62E-12	0.93 (0.91,0.95)	1.33E-12	0.92 (0.89,0.94)	0.0613	0.96 (0.92,1)	0.012593	0.002
rs2628301	53159273	Genotyped	C/T	0.27	1	1.01E-11	0.93 (0.91,0.95)	8.88E-12	0.92 (0.89,0.94)	0.0979	0.97 (0.93,1.01)	0.008736	0.001
rs2541236	53159832	Imputed	G/T	0.27	0.987	1.14E-11	0.93 (0.91,0.95)	1.65E-11	0.92 (0.89,0.94)	0.0848	0.96 (0.93,1.01)	0.012018	8.91e-4
rs2541242	53089687	Imputed	C/T	0.27	0.987	1.16E-11	0.93 (0.91,0.95)	9.65E-12	0.92 (0.89,0.94)	0.0995	0.97 (0.93,1.01)	0.009764	8.8e-4
rs1156287	53076799	Genotyped	A/G	0.28	1	3.41E-11	0.93 (0.91,0.95)	2.31E-11	0.92 (0.9,0.94)	0.158	0.97 (0.93,1.01)	0.00694	3.06e-4
rs2541245	53101200	Genotyped	A/G	0.28	1	3.97E-11	0.93 (0.91,0.95)	2.53E-11	0.92 (0.9,0.94)	0.161	0.97 (0.93,1.01)	0.007331	2.64e-4
rs11658717	53076986	Genotyped	A/G	0.28	1	4.05E-11	0.93 (0.91,0.95)	5.32E-11	0.92 (0.9,0.94)	0.129	0.97 (0.93,1.01)	0.010944	2.58e-4
rs2628309	53115419	Genotyped	A/G	0.28	1	4.10E-11	0.93 (0.91,0.95)	2.47E-11	0.92 (0.9,0.94)	0.175	0.97 (0.93,1.01)	0.005769	2.55e-4
rs2628308	53123573	Genotyped	G/C	0.28	1	4.23E-11	0.93 (0.91,0.95)	5.65E-11	0.92 (0.9,0.94)	0.131	0.97 (0.93,1.01)	0.011167	2.48e-4
rs2787505	53107432	Genotyped	C/T	0.28	1	4.61E-11	0.93 (0.91,0.95)	2.62E-11	0.92 (0.9,0.94)	0.167	0.97 (0.93,1.01)	0.00696	2.27e-4
rs17745534	53137506	Genotyped	G/A	0.28	1	5.25E-11	0.93 (0.91,0.95)	2.58E-11	0.92 (0.9,0.94)	0.171	0.97 (0.93,1.01)	0.006991	2.01e-4
rs9889687	53132411	Imputed	A/G	0.28	0.992	5.52E-11	0.93 (0.91,0.95)	2.87E-11	0.92 (0.9,0.94)	0.182	0.97 (0.93,1.01)	0.006642	1.91e-4
rs2787476	53089133	Imputed	C/T	0.28	0.981	5.59E-11	0.93 (0.91,0.95)	3.72E-11	0.92 (0.9,0.94)	0.143	0.97 (0.93,1.01)	0.011311	1.88e-4
rs1484776	53111383	Genotyped	C/A	0.28	1	5.61E-11	0.93 (0.91,0.95)	6.80E-11	0.92 (0.9,0.94)	0.149	0.97 (0.93,1.01)	0.009019	1.88e-4
rs2787507	53127924	Genotyped	T/C	0.28	1	5.70E-11	0.93 (0.91,0.95)	3.73E-11	0.92 (0.9,0.94)	0.159	0.97 (0.93,1.01)	0.007897	1.85e-4
rs9303363	53137974	Genotyped	G/A	0.28	1	5.74E-11	0.93 (0.91,0.95)	2.29E-11	0.92 (0.9,0.94)	0.191	0.97 (0.93,1.01)	0.005695	1.84e-4
chr17:53100141:I	53100141	Imputed	GA/G	0.28	0.987	5.87E-11	0.93 (0.91,0.95)	3.44E-11	0.92 (0.9,0.94)	0.162	0.97 (0.93,1.01)	0.009011	1.8e-4
rs2787494	53091181	Imputed	C/G	0.28	0.987	5.97E-11	0.93 (0.91,0.95)	3.37E-11	0.92 (0.9,0.94)	0.166	0.97 (0.93,1.01)	0.008615	1.77e-4
rs2628296	53166495	Genotyped	A/T	0.28	1	6.02E-11	0.93 (0.91,0.95)	6.93E-11	0.92 (0.9,0.94)	0.118	0.97 (0.93,1.01)	0.013887	1.75e-4
rs2628304	53157189	Imputed	A/G	0.28	0.988	6.09E-11	0.93 (0.91,0.95)	5.87E-11	0.92 (0.9,0.94)	0.142	0.97 (0.93,1.01)	0.010396	1.73e-4
rs1484769	53166651	Imputed	A/G	0.28	0.989	6.09E-11	0.93 (0.91,0.95)	4.11E-11	0.92 (0.9,0.94)	0.149	0.97 (0.93,1.01)	0.009746	1.73e-4

rs2787477	53089139	Imputed	C/G	0.28	0.987	6.10E-11	0.93 (0.91,0.95)	3.63E-11	0.92 (0.9,0.94)	0.16	0.97 (0.93,1.01)	0.009348	1.73e-4
rs2628305	53140427	Genotyped	C/A	0.28	1	6.26E-11	0.93 (0.91,0.95)	6.30E-11	0.92 (0.9,0.94)	0.14	0.97 (0.93,1.01)	0.011499	1.69e-4
rs2541237	53164037	Imputed	G/C	0.28	0.987	6.37E-11	0.93 (0.91,0.95)	4.09E-11	0.92 (0.9,0.94)	0.158	0.97 (0.93,1.01)	0.009019	1.66e-4
rs11079143	53083122	Genotyped	G/A	0.28	1	9.53E-11	0.93 (0.91,0.95)	5.37E-11	0.92 (0.9,0.94)	0.149	0.97 (0.93,1.01)	0.008871	1.12e-4
rs7218719	53245227	Genotyped	A/G	0.28	1	1.20E-10	0.93 (0.91,0.95)	4.93E-11	0.92 (0.9,0.94)	0.0908	0.97 (0.93,1.01)	0.016761	8.91e-5
rs12936639	53235982	Genotyped	G/A	0.28	1	2.28E-10	0.93 (0.91,0.95)	9.74E-11	0.92 (0.9,0.94)	0.112	0.97 (0.93,1.01)	0.016191	4.78e-5
rs7211787	53234912	Genotyped	A/T	0.28	1	2.61E-10	0.93 (0.91,0.95)	1.00E-10	0.92 (0.9,0.94)	0.12	0.97 (0.93,1.01)	0.014567	4.18e-5
rs9898886	53249679	Genotyped	A/G	0.28	1	3.11E-10	0.93 (0.92,0.95)	2.62E-10	0.92 (0.9,0.95)	0.0783	0.96 (0.93,1)	0.029238	3.52e-5
rs2541243	53091266	Imputed	G/A	0.3	0.933	3.28E-10	0.93 (0.91,0.95)	2.52E-10	0.92 (0.9,0.95)	0.116	0.97 (0.93,1.01)	0.021469	3.35e-5
chr17:53210312:D	53210312	Imputed	ATG/A	0.23	0.894	6.44E-10	0.93 (0.91,0.95)	2.23E-10	0.91 (0.89,0.94)	0.0781	0.96 (0.92,1)	0.028596	1.73e-5
rs10468513	53062903	Genotyped	C/A	0.27	1	1.48E-09	0.94 (0.92,0.96)	8.34E-09	0.93 (0.91,0.95)	0.128	0.97 (0.93,1.01)	0.026396	7.69e-6
rs9915832	53054697	Imputed	G/A	0.27	0.994	1.55E-09	0.94 (0.92,0.96)	8.22E-09	0.93 (0.91,0.95)	0.133	0.97 (0.93,1.01)	0.024531	7.37e-6
rs17818028	53062755	Genotyped	C/G	0.27	1	1.55E-09	0.94 (0.92,0.96)	5.32E-09	0.93 (0.9,0.95)	0.155	0.97 (0.93,1.01)	0.019138	7.35e-6
rs8078550	53053379	Imputed	T/G	0.27	0.994	1.65E-09	0.94 (0.92,0.96)	8.17E-09	0.93 (0.91,0.95)	0.133	0.97 (0.93,1.01)	0.024721	6.9e-6
rs9914732	53054367	Imputed	C/G	0.27	0.995	1.69E-09	0.94 (0.92,0.96)	8.23E-09	0.93 (0.91,0.95)	0.135	0.97 (0.93,1.01)	0.024461	6.74e-6
rs7221223	53059007	Genotyped	A/G	0.27	1	1.69E-09	0.94 (0.92,0.96)	5.39E-09	0.93 (0.9,0.95)	0.158	0.97 (0.93,1.01)	0.019138	6.77e-6
rs9893306	53054749	Imputed	A/T	0.27	0.995	1.71E-09	0.94 (0.92,0.96)	8.26E-09	0.93 (0.91,0.95)	0.135	0.97 (0.93,1.01)	0.024481	6.68e-6
rs9902687	53048924	Imputed	G/A	0.27	0.994	1.75E-09	0.94 (0.92,0.96)	8.69E-09	0.93 (0.91,0.95)	0.135	0.97 (0.93,1.01)	0.025009	6.52e-6
rs8080491	53049869	Imputed	T/A	0.27	0.995	1.79E-09	0.94 (0.92,0.96)	8.80E-09	0.93 (0.91,0.95)	0.135	0.97 (0.93,1.01)	0.024952	6.4e-6
rs9916642	53054497	Genotyped	T/C	0.27	1	1.80E-09	0.94 (0.92,0.96)	9.22E-09	0.93 (0.91,0.95)	0.127	0.97 (0.93,1.01)	0.026962	6.35e-6
rs7219874	53065807	Genotyped	C/T	0.27	1	1.80E-09	0.94 (0.92,0.96)	9.47E-09	0.93 (0.91,0.95)	0.127	0.97 (0.93,1.01)	0.026962	6.35e-6
rs6504949	53050133	Genotyped	T/G	0.27	1	1.81E-09	0.94 (0.92,0.96)	9.00E-09	0.93 (0.91,0.95)	0.129	0.97 (0.93,1.01)	0.026791	6.32e-6

rs6504948	53049987	Genotyped	T/C	0.27	1	1.82E-09	0.94 (0.92,0.96)	9.56E-09	0.93 (0.91,0.95)	0.13	0.97 (0.93,1.01)	0.02668	6.28e-6
chr17:53057391:I	53057391	Imputed	C/CTA	0.27	0.994	1.83E-09	0.94 (0.92,0.96)	8.84E-09	0.93 (0.91,0.95)	0.138	0.97 (0.93,1.01)	0.024638	6.23e-6
rs9897447	53048469	Imputed	T/C	0.27	0.993	1.84E-09	0.94 (0.92,0.96)	8.59E-09	0.93 (0.91,0.95)	0.14	0.97 (0.93,1.01)	0.024111	6.22e-6
rs9892173	53060214	Genotyped	C/T	0.27	1	1.85E-09	0.94 (0.92,0.96)	9.34E-09	0.93 (0.91,0.95)	0.134	0.97 (0.93,1.01)	0.026396	6.2e-6
rs6504951	53056975	Imputed	A/G	0.27	0.995	1.86E-09	0.94 (0.92,0.96)	8.72E-09	0.93 (0.91,0.95)	0.138	0.97 (0.93,1.01)	0.024599	6.16e-6
rs7222197	53047499	Genotyped	G/A	0.27	1	1.90E-09	0.94 (0.92,0.96)	9.26E-09	0.93 (0.91,0.95)	0.135	0.97 (0.93,1.01)	0.026109	6.02e-6
rs9896044	53048542	Genotyped	C/G	0.27	1	1.90E-09	0.94 (0.92,0.96)	9.88E-09	0.93 (0.91,0.95)	0.134	0.97 (0.93,1.01)	0.02668	6.03e-6
rs9891865	53060033	Genotyped	C/T	0.27	1	1.90E-09	0.94 (0.92,0.96)	9.57E-09	0.93 (0.91,0.95)	0.135	0.97 (0.93,1.01)	0.026396	6.03e-6
rs1990674	53061075	Genotyped	G/A	0.27	1	1.90E-09	0.94 (0.92,0.96)	9.57E-09	0.93 (0.91,0.95)	0.135	0.97 (0.93,1.01)	0.026396	6.03e-6
rs9902718	53061622	Genotyped	T/C	0.27	1	1.90E-09	0.94 (0.92,0.96)	9.57E-09	0.93 (0.91,0.95)	0.135	0.97 (0.93,1.01)	0.026396	6.03e-6
rs9903146	53057747	Imputed	C/T	0.27	0.994	1.94E-09	0.94 (0.92,0.96)	9.02E-09	0.93 (0.91,0.95)	0.14	0.97 (0.93,1.01)	0.024274	5.92e-6
rs17745344	53048319	Genotyped	A/G	0.27	1	1.95E-09	0.94 (0.92,0.96)	5.84E-09	0.93 (0.9,0.95)	0.163	0.97 (0.93,1.01)	0.019313	5.88e-6
rs9903220	53057865	Imputed	A/G	0.27	0.994	1.95E-09	0.94 (0.92,0.96)	9.07E-09	0.93 (0.91,0.95)	0.14	0.97 (0.93,1.01)	0.02423	5.88e-6
rs9903825	53057914	Imputed	G/A	0.27	0.994	1.95E-09	0.94 (0.92,0.96)	9.08E-09	0.93 (0.91,0.95)	0.141	0.97 (0.93,1.01)	0.024217	5.87e-6
rs9892976	53060329	Genotyped	G/A	0.27	1	1.95E-09	0.94 (0.92,0.96)	9.66E-09	0.93 (0.91,0.95)	0.137	0.97 (0.93,1.01)	0.025861	5.87e-6
rs16955471	53058807	Imputed	G/T	0.27	0.994	1.96E-09	0.94 (0.92,0.96)	9.28E-09	0.93 (0.91,0.95)	0.136	0.97 (0.93,1.01)	0.025543	5.86e-6
chr17:53059061:I	53059061	Imputed	T/TA	0.27	0.994	1.97E-09	0.94 (0.92,0.96)	9.25E-09	0.93 (0.91,0.95)	0.137	0.97 (0.93,1.01)	0.025287	5.83e-6
rs9903444	53057893	Imputed	C/T	0.27	0.994	1.98E-09	0.94 (0.92,0.96)	9.16E-09	0.93 (0.91,0.95)	0.142	0.97 (0.93,1.01)	0.023898	5.78e-6
rs9902950	53057764	Imputed	A/T	0.27	0.992	1.99E-09	0.94 (0.92,0.96)	9.01E-09	0.93 (0.91,0.95)	0.138	0.97 (0.93,1.01)	0.025725	5.75e-6
rs9894529	53055246	Imputed	A/T	0.27	0.993	2.00E-09	0.94 (0.92,0.96)	9.90E-09	0.93 (0.91,0.95)	0.138	0.97 (0.93,1.01)	0.025826	5.73e-6
rs9890971	53059776	Genotyped	A/T	0.27	1	2.01E-09	0.94 (0.92,0.96)	1.03E-08	0.93 (0.91,0.95)	0.138	0.97 (0.93,1.01)	0.026026	5.71e-6
rs28558726	53058676	Genotyped	A/G	0.27	1	2.02E-09	0.94 (0.92,0.96)	9.93E-09	0.93 (0.91,0.95)	0.137	0.97 (0.93,1.01)	0.025861	5.68e-6

rs6504950	53056471	Genotyped	G/A	0.27	1	2.04E-09	0.94 (0.92,0.96)	1.01E-08	0.93 (0.91,0.95)	0.136	0.97 (0.93,1.01)	0.026396	5.64e-6
rs56348638	53064550	Imputed	C/T	0.27	0.992	2.05E-09	0.94 (0.92,0.96)	9.23E-09	0.93 (0.91,0.95)	0.139	0.97 (0.93,1.01)	0.024889	5.6e-6
rs35051208	53052568	Imputed	C/T	0.27	0.985	2.22E-09	0.94 (0.92,0.96)	7.87E-09	0.93 (0.91,0.95)	0.149	0.97 (0.93,1.01)	0.02461	5.18e-6
rs9915183	53047137	Genotyped	G/A	0.27	1	2.27E-09	0.94 (0.92,0.96)	1.10E-08	0.93 (0.91,0.95)	0.139	0.97 (0.93,1.01)	0.026962	5.07e-6
chr17:53061695:D	53061695	Imputed	TTTTTC/T	0.27	0.961	2.39E-09	0.94 (0.92,0.96)	1.17E-08	0.93 (0.91,0.95)	0.123	0.97 (0.93,1.01)	0.034389	4.81e-6
rs8082471	53067993	Imputed	T/A	0.27	0.986	2.68E-09	0.94 (0.92,0.96)	1.20E-08	0.93 (0.91,0.95)	0.145	0.97 (0.93,1.01)	0.025898	4.32e-6
rs12940106	53067683	Imputed	A/G	0.27	0.982	2.98E-09	0.94 (0.92,0.96)	8.69E-09	0.93 (0.91,0.95)	0.17	0.97 (0.93,1.01)	0.021875	3.88e-6
chr17:53059774:I	53059774	Imputed	T/TA	0.26	0.947	4.11E-09	0.94 (0.92,0.96)	1.43E-08	0.93 (0.9,0.95)	0.129	0.97 (0.93,1.01)	0.03286	2.85e-6
rs9892848	53059775	Imputed	T/A	0.26	0.948	4.26E-09	0.94 (0.92,0.96)	1.45E-08	0.93 (0.9,0.95)	0.129	0.97 (0.93,1.01)	0.032976	2.75e-6
chr17:53059776:I	53059776	Imputed	A/AT	0.26	0.947	4.57E-09	0.94 (0.92,0.96)	1.45E-08	0.93 (0.9,0.95)	0.133	0.97 (0.93,1.01)	0.03062	2.57e-6
rs28394892	53066545	Imputed	A/T	0.27	0.963	4.91E-09	0.94 (0.92,0.96)	1.86E-08	0.93 (0.91,0.95)	0.125	0.97 (0.93,1.01)	0.037645	2.39e-6
rs9895808	53048442	Genotyped	C/G	0.27	1	8.75E-09	0.94 (0.92,0.96)	4.07E-08	0.93 (0.91,0.96)	0.157	0.97 (0.93,1.01)	0.029415	1.36e-6
rs2628298	53163478	Genotyped	A/T	0.26	1	1.37E-08	0.94 (0.92,0.96)	1.35E-08	0.93 (0.91,0.95)	0.188	0.97 (0.93,1.01)	0.037111	8.8e-7
rs12952253	53005716	Imputed	A/G	0.29	0.988	1.88E-08	0.94 (0.92,0.96)	1.66E-08	0.93 (0.91,0.95)	0.0789	0.96 (0.93,1)	0.070695	6.48e-7
ch17_pos53009280	53009280	Genotyped	G/T	0.29	1	2.24E-08	0.94 (0.92,0.96)	2.30E-08	0.93 (0.91,0.96)	0.0735	0.96 (0.93,1)	0.077663	5.48e-7
rs7208123	53006339	Genotyped	G/A	0.29	1	2.30E-08	0.94 (0.92,0.96)	2.15E-08	0.93 (0.91,0.96)	0.0744	0.96 (0.93,1)	0.075566	5.32e-7
chr17:53168208:D	53168208	Imputed	G/GTGTGTGTC	0.33	0.791	2.32E-08	0.94 (0.92,0.96)	2.02E-08	0.93 (0.9,0.95)	0.0867	0.96 (0.92,1.01)	0.088326	5.3e-7
rs7209926	53006324	Genotyped	A/G	0.29	1	2.42E-08	0.94 (0.92,0.96)	3.46E-08	0.93 (0.91,0.96)	0.0645	0.96 (0.93,1)	0.091482	5.07e-7
rs9916547	53006890	Imputed	G/A	0.29	0.991	2.60E-08	0.94 (0.92,0.96)	3.90E-08	0.93 (0.91,0.96)	0.0636	0.96 (0.93,1)	0.093246	4.74e-7
rs8065361	53008853	Imputed	A/T	0.29	0.993	2.60E-08	0.94 (0.92,0.96)	3.77E-08	0.93 (0.91,0.96)	0.0642	0.96 (0.93,1)	0.091554	4.73e-7
rs34652394	53013367	Imputed	C/T	0.29	0.991	2.75E-08	0.94 (0.92,0.96)	2.36E-08	0.93 (0.91,0.96)	0.0793	0.96 (0.93,1)	0.071315	4.48e-7
rs35465233	53013357	Imputed	G/A	0.29	0.992	2.78E-08	0.94 (0.92,0.96)	2.37E-08	0.93 (0.91,0.96)	0.0796	0.96 (0.93,1)	0.071168	4.43e-7

rs9912589	53009971	Imputed	G/A	0.29	0.993	2.80E-08	0.94 (0.92,0.96)	3.93E-08	0.93 (0.91,0.96)	0.0634	0.96 (0.92,1)	0.092733	4.4e-7
rs9900816	53012057	Genotyped	G/A	0.29	1	2.81E-08	0.94 (0.92,0.96)	4.13E-08	0.93 (0.91,0.96)	0.0676	0.96 (0.93,1)	0.091235	4.38e-7
rs17745183	53013595	Imputed	G/T	0.29	0.991	2.83E-08	0.94 (0.92,0.96)	2.42E-08	0.93 (0.91,0.96)	0.0796	0.96 (0.93,1)	0.071434	4.37e-7
rs12603899	53010098	Genotyped	T/C	0.29	1	2.88E-08	0.94 (0.92,0.96)	3.99E-08	0.93 (0.91,0.96)	0.068	0.96 (0.93,1)	0.09014	4.29e-7
rs9891704	53010755	Genotyped	A/G	0.29	1	2.91E-08	0.94 (0.92,0.96)	3.96E-08	0.93 (0.91,0.96)	0.0684	0.96 (0.93,1)	0.090802	4.25e-7
rs9907961	53012927	Genotyped	C/G	0.29	1	2.91E-08	0.94 (0.92,0.96)	4.13E-08	0.93 (0.91,0.96)	0.0645	0.96 (0.93,1)	0.094821	4.25e-7
rs9909096	53013289	Imputed	G/T	0.29	0.993	3.38E-08	0.94 (0.92,0.96)	4.77E-08	0.93 (0.91,0.96)	0.0674	0.96 (0.93,1)	0.093011	3.67e-7
ch17_pos52975265	52975265	Genotyped	C/G	0.19	1	5.30E-08	0.94 (0.91,0.96)	7.25E-08	0.92 (0.9,0.95)	0.0453	0.95 (0.91,1)	0.183278	2.37e-7
rs35647022	53008071	Imputed	C/T	0.27	0.986	8.79E-08	0.94 (0.92,0.96)	9.34E-08	0.93 (0.91,0.96)	0.139	0.97 (0.93,1.01)	0.045415	1.45e-7
chr17:53035381:D	53035381	Imputed	CTGAGGCCTGCAA/C	0.27	0.983	9.97E-08	0.94 (0.92,0.96)	1.13E-07	0.93 (0.91,0.96)	0.131	0.97 (0.93,1.01)	0.049383	1.29e-7
ch17_pos52974643	52974643	Genotyped	G/C	0.15	1	1.02E-07	0.93 (0.91,0.96)	5.68E-07	0.92 (0.89,0.95)	0.125	0.96 (0.91,1.01)	0.1105	1.26e-7
rs17817877	53034628	Imputed	T/A	0.27	0.991	1.29E-07	0.94 (0.92,0.96)	1.54E-07	0.94 (0.91,0.96)	0.153	0.97 (0.93,1.01)	0.044523	1,00E-07
rs9895901	52984885	Genotyped	A/G	0.3	1	1.70E-07	0.95 (0.93,0.97)	1.82E-07	0.94 (0.91,0.96)	0.105	0.97 (0.93,1.01)	0.083674	7.66e-8
rs9904377	52985982	Genotyped	G/A	0.3	1	1.78E-07	0.95 (0.93,0.97)	1.79E-07	0.94 (0.91,0.96)	0.104	0.97 (0.93,1.01)	0.085355	7.35e-8
rs2958913	52976942	Genotyped	G/C	0.2	1	1.84E-07	0.94 (0.92,0.96)	1.78E-07	0.93 (0.9,0.95)	0.057	0.96 (0.91,1)	0.199376	7.12e-8
rs2877634	52987545	Imputed	A/G	0.31	0.984	1.91E-07	0.95 (0.93,0.97)	1.83E-07	0.94 (0.92,0.96)	0.0914	0.97 (0.93,1.01)	0.098864	6.85e-8
chr17:52992128:D	52992128	Imputed	TA/T	0.3	0.981	1.91E-07	0.95 (0.93,0.97)	1.95E-07	0.94 (0.91,0.96)	0.104	0.97 (0.93,1.01)	0.083135	6.87e-8
rs7214573	53006654	Genotyped	T/C	0.27	1	2.02E-07	0.95 (0.93,0.97)	2.32E-07	0.94 (0.91,0.96)	0.131	0.97 (0.93,1.01)	0.059508	6.5e-8
chr17:53030584:I	53030584	Imputed	C/CA	0.27	0.994	2.04E-07	0.95 (0.93,0.97)	2.29E-07	0.94 (0.91,0.96)	0.158	0.97 (0.93,1.01)	0.051159	6.44e-8
chr17:53067647:D	53067647	Imputed	TCAGGCGACA/T	0.26	0.957	2.14E-07	0.94 (0.92,0.96)	7.95E-07	0.94 (0.91,0.96)	0.175	0.97 (0.93,1.01)	0.074036	6.15e-8
chr17:53041890:D	53041890	Imputed	TACTTTA/T	0.27	0.988	2.21E-07	0.95 (0.93,0.97)	3.02E-07	0.94 (0.91,0.96)	0.152	0.97 (0.93,1.01)	0.057636	5.97e-8
rs8071094	53086937	Genotyped	C/T	0.1	1	2.22E-07	0.92 (0.89,0.95)	1.06E-05	0.92 (0.89,0.96)	0.0613	0.94 (0.89,1)	0.538967	5.92e-8

chr17:53044475:I	53044475	Imputed	T/TA	0.27	0.977	2.23E-07	0.95 (0.93,0.97)	3.07E-07	0.94 (0.91,0.96)	0.159	0.97 (0.93,1.01)	0.056581	5.9e-8
rs12937006	53030461	Genotyped	C/T	0.27	1	2.24E-07	0.95 (0.93,0.97)	2.78E-07	0.94 (0.91,0.96)	0.15	0.97 (0.93,1.01)	0.053969	5.89e-8
rs9889559	52983722	Imputed	T/C	0.3	0.992	2.26E-07	0.95 (0.93,0.97)	3.82E-07	0.94 (0.92,0.96)	0.0805	0.97 (0.93,1)	0.130636	5.82e-8
rs9913784	52989071	Genotyped	A/T	0.3	1	2.26E-07	0.95 (0.93,0.97)	2.28E-07	0.94 (0.91,0.96)	0.0934	0.97 (0.93,1.01)	0.093189	5.83e-8
rs12949538	53010670	Genotyped	C/T	0.27	1	2.28E-07	0.95 (0.93,0.97)	2.61E-07	0.94 (0.91,0.96)	0.135	0.97 (0.93,1.01)	0.059804	5.77e-8
rs35097996	53021923	Imputed	T/C	0.27	0.993	2.29E-07	0.95 (0.93,0.97)	3.06E-07	0.94 (0.91,0.96)	0.126	0.97 (0.93,1.01)	0.064026	5.76e-8
rs17745189	53013980	Genotyped	A/G	0.27	1	2.34E-07	0.95 (0.93,0.97)	2.67E-07	0.94 (0.91,0.96)	0.141	0.97 (0.93,1.01)	0.057168	5.64e-8
rs3087650	53029328	Genotyped	G/A	0.27	1	2.36E-07	0.95 (0.93,0.97)	2.81E-07	0.94 (0.91,0.96)	0.155	0.97 (0.93,1.01)	0.052747	5.6e-8
rs8066588	53025689	Genotyped	C/T	0.27	1	2.37E-07	0.95 (0.93,0.97)	2.91E-07	0.94 (0.91,0.96)	0.14	0.97 (0.93,1.01)	0.054664	5.57e-8
rs8076984	53035428	Imputed	T/C	0.27	0.996	2.39E-07	0.95 (0.93,0.97)	2.75E-07	0.94 (0.91,0.96)	0.158	0.97 (0.93,1.01)	0.051294	5.53e-8
rs9908752	52983216	Genotyped	C/T	0.3	1	2.40E-07	0.95 (0.93,0.97)	3.83E-07	0.94 (0.92,0.96)	0.0764	0.96 (0.93,1)	0.14129	5.5e-8
rs8066833	53025475	Imputed	A/G	0.27	0.994	2.43E-07	0.95 (0.93,0.97)	3.12E-07	0.94 (0.91,0.96)	0.132	0.97 (0.93,1.01)	0.061724	5.44e-8
rs17817847	53018281	Imputed	T/A	0.27	0.993	2.44E-07	0.95 (0.93,0.97)	3.27E-07	0.94 (0.91,0.96)	0.132	0.97 (0.93,1.01)	0.061394	5.42e-8
rs12949718	52981853	Genotyped	C/T	0.31	1	2.49E-07	0.95 (0.93,0.97)	3.76E-07	0.94 (0.92,0.96)	0.0787	0.97 (0.93,1)	0.143385	5.32e-8
chr17:53035494:D	53035494	Imputed	GA/G	0.27	0.992	2.49E-07	0.95 (0.93,0.97)	2.77E-07	0.94 (0.91,0.96)	0.163	0.97 (0.93,1.01)	0.05085	5.31e-8
rs12936860	53030226	Genotyped	G/A	0.27	1	2.50E-07	0.95 (0.93,0.97)	3.00E-07	0.94 (0.91,0.96)	0.156	0.97 (0.93,1.01)	0.05249	5.28e-8
rs9910653	52992268	Genotyped	G/A	0.31	1	2.59E-07	0.95 (0.93,0.97)	2.38E-07	0.94 (0.92,0.96)	0.0965	0.97 (0.93,1.01)	0.111745	5.12e-8
chr17:53016155:D	53016155	Imputed	CTT/C	0.27	0.993	2.61E-07	0.95 (0.93,0.97)	3.73E-07	0.94 (0.91,0.96)	0.136	0.97 (0.93,1.01)	0.062538	5.07e-8
rs17817901	53038745	Imputed	A/G	0.27	0.995	2.66E-07	0.95 (0.93,0.97)	2.96E-07	0.94 (0.91,0.96)	0.161	0.97 (0.93,1.01)	0.052265	4.99e-8
chr17:53026823:D	53026823	Imputed	TAAAAG/T	0.27	0.992	2.79E-07	0.95 (0.93,0.97)	3.01E-07	0.94 (0.91,0.96)	0.15	0.97 (0.93,1.01)	0.054805	4.76e-8
rs12150038	53041520	Genotyped	T/G	0.27	1	2.82E-07	0.95 (0.93,0.97)	3.40E-07	0.94 (0.91,0.96)	0.163	0.97 (0.93,1.01)	0.052667	4.71e-8
rs9914088	52979985	Genotyped	T/C	0.3	1	2.84E-07	0.95 (0.93,0.97)	4.88E-07	0.94 (0.92,0.96)	0.076	0.96 (0.93,1)	0.147245	4.68e-8

rs1802212	53038654	Genotyped	A/C	0.27	1	2.85E-07	0.95 (0.93,0.97)	3.36E-07	0.94 (0.91,0.96)	0.157	0.97 (0.93,1.01)	0.05377	4.66e-8
rs8073158	53028615	Imputed	C/A	0.27	0.993	2.89E-07	0.95 (0.93,0.97)	3.41E-07	0.94 (0.91,0.96)	0.15	0.97 (0.93,1.01)	0.057298	4.6e-8
rs17745231	53036529	Genotyped	C/G	0.27	1	3.00E-07	0.95 (0.93,0.97)	3.42E-07	0.94 (0.91,0.96)	0.161	0.97 (0.93,1.01)	0.052315	4.43e-8
rs12938118	53037359	Genotyped	T/C	0.27	1	3.10E-07	0.95 (0.93,0.97)	3.51E-07	0.94 (0.91,0.96)	0.161	0.97 (0.93,1.01)	0.052537	4.3e-8
rs12602751	52994174	Genotyped	C/A	0.31	1	3.11E-07	0.95 (0.93,0.97)	3.32E-07	0.94 (0.92,0.96)	0.0889	0.97 (0.93,1.01)	0.119844	4.29e-8
rs12937760	53016130	Genotyped	G/T	0.27	1	3.11E-07	0.95 (0.93,0.97)	3.72E-07	0.94 (0.91,0.96)	0.155	0.97 (0.93,1.01)	0.0542	4.29e-8
rs7216138	52995664	Genotyped	G/A	0.31	1	3.24E-07	0.95 (0.93,0.97)	3.22E-07	0.94 (0.92,0.96)	0.0892	0.97 (0.93,1.01)	0.118214	4.12e-8
rs12951542	53041965	Genotyped	T/C	0.27	1	3.25E-07	0.95 (0.93,0.97)	4.00E-07	0.94 (0.91,0.96)	0.161	0.97 (0.93,1.01)	0.055946	4.11e-8
ch17_pos53017747	53017747	Genotyped	A/G	0.27	1	3.69E-07	0.95 (0.93,0.97)	4.03E-07	0.94 (0.91,0.96)	0.141	0.97 (0.93,1.01)	0.060556	3.64e-8
chr17:53041892:D	53041892	Imputed	CTTTAACT/C	0.27	0.986	4.48E-07	0.95 (0.93,0.97)	5.12E-07	0.94 (0.91,0.96)	0.16	0.97 (0.93,1.01)	0.061572	3.01e-8
rs17712917	53271365	Imputed	T/G	0.22	0.991	4.50E-07	0.94 (0.92,0.96)	1.79E-07	0.93 (0.91,0.96)	0.285	0.98 (0.93,1.02)	0.016876	3,00E-08
rs7212321	53258437	Genotyped	C/G	0.22	1	4.95E-07	0.94 (0.92,0.96)	1.21E-07	0.93 (0.9,0.96)	0.315	0.98 (0.94,1.02)	0.012958	2.74e-8
rs8075983	53271918	Genotyped	G/A	0.22	1	5.06E-07	0.94 (0.92,0.96)	2.58E-07	0.93 (0.91,0.96)	0.27	0.98 (0.93,1.02)	0.020872	2.68e-8
rs714896	53256982	Imputed	T/C	0.22	0.99	5.12E-07	0.94 (0.92,0.96)	1.22E-07	0.93 (0.9,0.95)	0.311	0.98 (0.93,1.02)	0.012228	2.65e-8
rs714897	53256579	Genotyped	C/T	0.22	1	5.36E-07	0.94 (0.92,0.96)	1.41E-07	0.93 (0.9,0.96)	0.333	0.98 (0.94,1.02)	0.011172	2.53e-8
rs9910034	52983596	Imputed	G/A	0.18	0.979	6.11E-07	0.94 (0.92,0.96)	1.21E-06	0.93 (0.9,0.96)	0.0563	0.95 (0.91,1)	0.279546	2.23e-8
rs9907270	53266415	Genotyped	G/A	0.22	1	6.38E-07	0.94 (0.92,0.97)	2.09E-07	0.93 (0.91,0.96)	0.302	0.98 (0.93,1.02)	0.013714	2.14e-8
rs9914836	52997875	Genotyped	C/G	0.28	1	7.11E-07	0.95 (0.93,0.97)	1.27E-06	0.94 (0.92,0.96)	0.105	0.97 (0.93,1.01)	0.10606	1.93e-8
chr17:52998317:I	52998317	Imputed	G/GAC	0.29	0.984	7.43E-07	0.95 (0.93,0.97)	1.08E-06	0.94 (0.92,0.96)	0.0934	0.97 (0.93,1.01)	0.121049	1.85e-8
rs9915913	52998168	Genotyped	G/T	0.29	1	7.98E-07	0.95 (0.93,0.97)	1.03E-06	0.94 (0.92,0.96)	0.106	0.97 (0.93,1.01)	0.106638	1.73e-8
rs59623427	53121907	Imputed	T/C	0.46	0.984	8.01E-07	0.95 (0.94,0.97)	1.55E-06	0.95 (0.93,0.97)	0.16	0.97 (0.94,1.01)	0.280645	1.72e-8
rs7208403	53046667	Genotyped	C/A	0.4	1	1.01E-06	0.95 (0.93,0.97)	9.52E-06	0.95 (0.93,0.97)	0.183	0.97 (0.94,1.01)	0.249619	1.37e-8

rs7226272	53046447	Genotyped	G/A	0.4	1	1.08E-06	0.95 (0.93,0.97)	9.71E-06	0.95 (0.93,0.97)	0.182	0.97 (0.94,1.01)	0.258887	1.29e-8
rs9914596	52996551	Genotyped	C/T	0.29	1	1.11E-06	0.95 (0.93,0.97)	1.26E-06	0.94 (0.92,0.96)	0.119	0.97 (0.93,1.01)	0.099011	1.25e-8
rs12937360	53046298	Genotyped	C/T	0.4	1	1.21E-06	0.95 (0.94,0.97)	1.01E-05	0.95 (0.93,0.97)	0.196	0.98 (0.94,1.01)	0.24398	1.16e-8
rs35079296	52994210	Genotyped	T/G	0.29	1	1.29E-06	0.95 (0.93,0.97)	1.62E-06	0.94 (0.92,0.97)	0.118	0.97 (0.93,1.01)	0.116142	1.09e-8
rs66898273	52993956	Imputed	C/T	0.29	0.991	1.38E-06	0.95 (0.93,0.97)	1.78E-06	0.94 (0.92,0.97)	0.115	0.97 (0.93,1.01)	0.122404	1.02e-8
rs72831295	53043804	Imputed	T/C	0.28	0.985	1.47E-06	0.95 (0.93,0.97)	1.36E-06	0.94 (0.92,0.96)	0.147	0.97 (0.93,1.01)	0.097356	9.6e-9
rs1962045	52996150	Genotyped	C/T	0.29	1	1.50E-06	0.95 (0.93,0.97)	1.84E-06	0.94 (0.92,0.97)	0.122	0.97 (0.93,1.01)	0.113386	9.4e-9
rs9897646	52985351	Genotyped	G/C	0.28	1	1.64E-06	0.95 (0.93,0.97)	1.98E-06	0.94 (0.92,0.97)	0.161	0.97 (0.93,1.01)	0.081252	8.63e-9
rs17745123	52988232	Genotyped	G/T	0.28	1	1.64E-06	0.95 (0.93,0.97)	2.00E-06	0.94 (0.92,0.97)	0.146	0.97 (0.93,1.01)	0.08771	8.65e-9
rs9899602	52986908	Genotyped	T/C	0.28	1	1.66E-06	0.95 (0.93,0.97)	2.00E-06	0.94 (0.92,0.97)	0.163	0.97 (0.93,1.01)	0.082072	8.52e-9
rs9899545	52986845	Genotyped	T/C	0.28	1	1.67E-06	0.95 (0.93,0.97)	2.02E-06	0.94 (0.92,0.97)	0.153	0.97 (0.93,1.01)	0.087512	8.46e-9
rs28564882	52989598	Genotyped	C/T	0.29	1	1.70E-06	0.95 (0.93,0.97)	2.27E-06	0.94 (0.92,0.97)	0.0988	0.97 (0.93,1.01)	0.143936	8.36e-9
rs2628297	53164243	Imputed	A/G	0.47	0.986	1.76E-06	0.95 (0.94,0.97)	4.34E-06	0.95 (0.93,0.97)	0.165	0.97 (0.94,1.01)	0.344238	8.09e-9
rs2628300	53160154	Genotyped	A/G	0.47	1	2.66E-06	0.96 (0.94,0.97)	6.02E-06	0.95 (0.93,0.97)	0.179	0.98 (0.94,1.01)	0.341058	5.42e-9
rs12449786	53085570	Genotyped	A/C	0.47	1	2.75E-06	0.96 (0.94,0.97)	6.17E-06	0.95 (0.93,0.97)	0.159	0.97 (0.94,1.01)	0.36557	5.24e-9
rs2541235	53157394	Genotyped	T/C	0.47	1	3.03E-06	0.96 (0.94,0.97)	7.31E-06	0.95 (0.93,0.97)	0.179	0.98 (0.94,1.01)	0.357866	4.78e-9
rs2628302	53158361	Genotyped	A/G	0.47	1	3.55E-06	0.96 (0.94,0.97)	8.23E-06	0.95 (0.93,0.97)	0.188	0.98 (0.94,1.01)	0.36058	4.11e-9
rs2787491	53206519	Imputed	G/C	0.44	0.924	1.37E-05	1.05 (1.02,1.07)	5.57E-07	1.06 (1.04,1.09)	0.655	1.01 (0.97,1.05)	0.001351	1.13e-9
chr17:53195938:D	53195938	Imputed	A/AAATG	0.43	0.904	1.43E-05	1.05 (1.02,1.07)	3.34E-07	1.06 (1.04,1.09)	0.673	1.01 (0.97,1.05)	0.001166	1.09e-9
rs2958915	52975892	Genotyped	G/T	0.43	1	4.47E-05	0.96 (0.94,0.98)	9.48E-05	0.96 (0.94,0.98)	0.116	0.97 (0.94,1.01)	0.572598	3.67e-10
rs244375	53183659	Imputed	A/G	0.38	0.926	6.73E-05	1.04 (1.02,1.06)	1.47E-06	1.06 (1.04,1.09)	0.993	1 (0.96,1.04)	0.000426	2.49e-10
rs8080876	53175267	Genotyped	T/C	0.41	1	6.82E-05	1.04 (1.02,1.06)	3.09E-06	1.06 (1.03,1.08)	0.652	1.01 (0.97,1.05)	0.002776	2.46e-10

rs2787487	53209382	Imputed	C/G	0.4	0.99	7.92E-05	1.04 (1.02,1.06)	1.61E-06	1.06 (1.03,1.08)	0.967	1 (0.96,1.04)	0.000563	2.13e-10
rs2787504	53178341	Genotyped	A/G	0.4	1	8.91E-05	1.04 (1.02,1.06)	2.39E-06	1.06 (1.03,1.08)	0.835	1 (0.97,1.04)	0.000963	1.91e-10
rs2529508	53212572	Imputed	T/A	0.41	0.981	9.50E-05	1.04 (1.02,1.06)	2.21E-06	1.06 (1.03,1.08)	0.884	1 (0.97,1.04)	0.000924	1.8e-10
rs244347	53196350	Imputed	G/T	0.4	0.985	9.69E-05	1.04 (1.02,1.06)	2.20E-06	1.06 (1.03,1.08)	0.924	1 (0.96,1.04)	0.000793	1.76e-10
rs2787484	53210120	Genotyped	T/C	0.4	1	9.72E-05	1.04 (1.02,1.06)	2.07E-06	1.06 (1.03,1.08)	0.917	1 (0.97,1.04)	0.000791	1.76e-10
chr17:53195933:D	53195933	Imputed	G/GAAAT	0.4	0.992	9.77E-05	1.04 (1.02,1.06)	2.11E-06	1.06 (1.03,1.08)	0.924	1 (0.96,1.04)	0.000814	1.75e-10
rs2787474	53214634	Imputed	C/T	0.4	0.993	9.83E-05	1.04 (1.02,1.06)	2.35E-06	1.06 (1.03,1.08)	0.894	1 (0.97,1.04)	0.00087	1.74e-10
rs2529505	53206808	Imputed	A/G	0.4	0.992	9.88E-05	1.04 (1.02,1.06)	1.97E-06	1.06 (1.03,1.08)	0.885	1 (0.97,1.04)	0.000858	1.73e-10
rs171513	53190426	Genotyped	G/A	0.4	1	9.90E-05	1.04 (1.02,1.06)	2.04E-06	1.06 (1.03,1.08)	0.925	1 (0.96,1.04)	0.000732	1.73e-10
rs244354	53194142	Genotyped	T/C	0.4	1	1.00E-04	1.04 (1.02,1.06)	2.03E-06	1.06 (1.03,1.08)	0.949	1 (0.96,1.04)	0.000675	1.71e-10
rs244341	53198922	Genotyped	A/G	0.4	1	1.00E-04	1.04 (1.02,1.06)	2.20E-06	1.06 (1.03,1.08)	0.929	1 (0.96,1.04)	0.000784	1.71e-10

a: Build 37 coordinates on chromosome 17.
b: Major and minor allele.
c: Minor allele frequency.
d: IMPUTE2 info score.
e: P-value, Odds ratio (OR) and 95% Confidence Interval (CI) for association with Overall Breast Cancer Risk, Estrogen Positive (ER+) and Estrogen Negative (ER-) disease.
f: Case-only analysis, treating subtype status as a dependent variable, models adjusted for principal components and per-study fixed-effects.
g: Relative Likelihood with respect to lead SNP rs2787486.

Supplementary Table 2: Markers correlated (r^2 > 0.6) with, and likelihood ratio (RL) of <1:100 relative to rs2787486 with respect to overall Risk.

							Overall Risk [f]		ER+ [f]		ER- [f]		Heterogeneity [g]
SNP	Position [a]	Type	Maj/Min [b]	MAF [c]	Info [d]	Rsq [e]	P-value	OR (95%CI)	P-value	OR (95%CI)	P-value	OR (95%CI)	P-value
rs2787497	53176211	Genotyped	A/G	0.27	1	0.89	9.27E-13	0.93 (0.91,0.95)	3.52E-13	0.91 (0.89,0.93)	0.0702	0.96 (0.92,1.00)	0.007
rs8082622	53177567	Genotyped	C/T	0.27	1	0.9	7.06E-13	0.93 (0.91,0.94)	6.24E-13	0.91 (0.89,0.94)	0.0528	0.96 (0.92,1.00)	0.011
rs2529510	53182046	Imputed	T/A	0.3	0.91	0.84	6.92E-14	0.92 (0.90,0.94)	2.08E-14	0.91 (0.88,0.93)	0.0645	0.96 (0.92,1.00)	0.007
rs244373	53184949	Imputed	C/T	0.29	0.98	0.99	7.30E-14	0.92 (0.90,0.94)	7.84E-14	0.91 (0.89,0.93)	0.0315	0.96 (0.92,1.00)	0.012
rs244358	53192231	Genotyped	C/T	0.27	1	0.89	9.68E-13	0.93 (0.91,0.95)	2.97E-13	0.91 (0.89,0.93)	0.0893	0.97 (0.93,1.01)	0.005
rs187242	53192946	Imputed	T/A	0.29	0.98	0.98	8.17E-13	0.93 (0.91,0.95)	2.65E-12	0.92 (0.89,0.94)	0.0278	0.96 (0.92,1.00)	0.030
rs244353	53194769	Genotyped	G/A	0.29	1	0.99	5.75E-14	0.92 (0.90,0.94)	5.96E-14	0.91 (0.89,0.93)	0.0275	0.96 (0.92,1.00)	0.015
rs244348	53196291	Genotyped	G/A	0.27	1	0.9	7.08E-13	0.93 (0.91,0.94)	5.20E-13	0.91 (0.89,0.93)	0.0547	0.96 (0.92,1.00)	0.011
rs244342	53198407	Genotyped	C/T	0.29	1	0.99	6.73E-14	0.92 (0.90,0.94)	7.48E-14	0.91 (0.89,0.93)	0.03	0.96 (0.92,1.00)	0.015
rs244338	53200418	Genotyped	G/A	0.29	1	0.99	6.21E-14	0.92 (0.90,0.94)	6.70E-14	0.91 (0.89,0.93)	0.0276	0.96 (0.92,1.00)	0.016
rs244337	53201124	Imputed	G/T	0.29	0.99	0.99	6.60E-14	0.92 (0.90,0.94)	6.29E-14	0.91 (0.89,0.93)	0.0304	0.96 (0.92,1.00)	0.014
rs244336	53201502	Genotyped	G/A	0.27	1	0.9	8.80E-13	0.93 (0.91,0.95)	7.60E-13	0.91 (0.89,0.94)	0.047	0.96 (0.92,1.00)	0.013
chr17:53205761:D	53205761	Imputed	AT/A	0.27	0.99	0.9	9.42E-13	0.93 (0.91,0.95)	7.95E-13	0.91 (0.89,0.94)	0.0552	0.96 (0.92,1.00)	0.012
rs2628321	53205917	Genotyped	A/G	0.29	1	0.99	5.88E-14	0.92 (0.90,0.94)	7.01E-14	0.91 (0.89,0.93)	0.0277	0.96 (0.92,1.00)	0.015
rs2787486	53209774	Imputed	A/C	0.28	0.96	-	8.96E-15	0.92 (0.90,0.94)	1.39E-14	0.91 (0.88,0.93)	0.0177	0.95 (0.91,0.99)	0.017
rs2787481	53211110	Genotyped	T/C	0.27	1	0.9	6.44E-13	0.92 (0.91,0.94)	2.93E-13	0.91 (0.89,0.93)	0.0637	0.96 (0.92,1.00)	0.008
rs244315	53214654	Genotyped	C/T	0.27	1	0.9	4.72E-13	0.92 (0.91,0.94)	4.27E-13	0.91 (0.89,0.93)	0.0493	0.96 (0.92,1.00)	0.011
rs244317	53216985	Genotyped	A/T	0.27	1	0.9	5.79E-13	0.92 (0.91,0.94)	2.74E-13	0.91 (0.89,0.93)	0.0649	0.96 (0.92,1.00)	0.008
rs2628316	53219837	Genotyped	A/G	0.29	1	0.99	6.00E-14	0.92 (0.90,0.94)	1.17E-13	0.91 (0.89,0.93)	0.0276	0.96 (0.92,1.00)	0.016
chr17:53221365:I	53221365	Imputed	T/TGC	0.29	0.98	0.99	9.68E-14	0.92 (0.90,0.94)	1.44E-13	0.91 (0.89,0.93)	0.0254	0.95 (0.92,0.99)	0.018
rs244318	53221368	Imputed	A/G	0.29	0.98	0.97	4.19E-13	0.93 (0.91,0.95)	3.56E-13	0.91 (0.89,0.94)	0.036	0.96 (0.92,1.00)	0.015
rs244319	53222874	Genotyped	G/A	0.29	1	0.98	2.19E-13	0.93 (0.91,0.94)	4.18E-13	0.91 (0.89,0.94)	0.028	0.96 (0.92,1.00)	0.022
rs244320	53222920	Genotyped	C/T	0.29	1	0.98	2.19E-13	0.93 (0.91,0.94)	4.18E-13	0.91 (0.89,0.94)	0.028	0.96 (0.92,1.00)	0.022
rs244321	53223998	Imputed	T/A	0.29	0.99	0.98	1.72E-13	0.92 (0.91,0.94)	3.22E-13	0.91 (0.89,0.94)	0.0245	0.95 (0.92,0.99)	0.023
rs10432032	53224088	Genotyped	A/G	0.29	1	0.98	2.47E-13	0.93 (0.91,0.94)	5.33E-13	0.91 (0.89,0.94)	0.0281	0.96 (0.92,1.00)	0.023
rs244322	53224759	Imputed	C/T	0.29	0.99	0.98	2.03E-13	0.93 (0.91,0.94)	3.35E-13	0.91 (0.89,0.94)	0.0281	0.96 (0.92,1.00)	0.021
rs2628315	53226622	Genotyped	G/A	0.29	1	0.99	9.02E-14	0.92 (0.90,0.94)	1.78E-13	0.91 (0.89,0.93)	0.0259	0.96 (0.92,0.99)	0.018

rs244294	53228543	Imputed	A/G	0.29	0.99	0.98	9.28E-13	0.93 (0.91,0.95)	1.21E-12	0.91 (0.89,0.94)	0.0354	0.96 (0.92,1.00)	0.022

a: Build 37 coordinates on chromosome 17.
b: Major and minor allele.
c: Minor Allele Frequency
d: IMPUTE2 info score.
e: Correlation with respect to lead SNP rs2787486.
f: P-value, Odds ratio (OR) and 95% Confidence Interval (CI) for association with Overall Breast Cancer Risk, Estrogen Positive (ER+) and Estrogen Negative (ER-) disease.
g: Case-only analysis, treating subtype status as a dependent variable, models adjusted for principal components and per-study fixed-effects.

Supplementary Table 3: Based on data from all Asian studies, association results for genotyped markers with MAF≥0.01 ranked by Overall Risk association.										
				Overall Risk [d]		ER+ [d]		ER- [d]		Heterogeneity [e]
SNP	Position [a]	Maj/Min [b]	MAF [c]	P-value	OR (95% CI)	P-value	OR (95% CI)	P-value	OR (95% CI)	P-value
rs244353	53194769	G/A	0.28	0.00257	0.92 (0.87,0.97)	0.00506	0.91 (0.85,0.97)	0.0443	0.91 (0.83,1)	0.765
rs244338	53200418	G/A	0.28	0.00278	0.92 (0.87,0.97)	0.00581	0.91 (0.85,0.97)	0.041	0.91 (0.83,1)	0.798
rs2628321	53205917	A/G	0.28	0.00281	0.92 (0.87,0.97)	0.0054	0.91 (0.85,0.97)	0.0447	0.91 (0.83,1)	0.775
rs244342	53198407	G/A	0.28	0.003	0.92 (0.87,0.97)	0.00642	0.91 (0.85,0.97)	0.0393	0.91 (0.83,1)	0.849
rs12449538	53111121	G/A	0.28	0.0041	0.92 (0.87,0.97)	0.0053	0.91 (0.85,0.97)	0.0582	0.92 (0.84,1)	0.698
rs2787500	53169806	A/C	0.29	0.00543	0.92 (0.87,0.98)	0.00683	0.91 (0.85,0.97)	0.0541	0.92 (0.84,1)	0.813
rs2628315	53226622	G/A	0.29	0.00594	0.92 (0.87,0.98)	0.00655	0.91 (0.85,0.97)	0.0778	0.92 (0.84,1.01)	0.715
rs7211784	53279433	A/G	0.44	0.012	0.94 (0.89,0.99)	0.000987	0.9 (0.85,0.96)	0.00978	0.9 (0.83,0.97)	0.868
rs2628316	53219837	A/G	0.28	0.0151	0.93 (0.88,0.99)	0.0115	0.91 (0.85,0.98)	0.19	0.94 (0.86,1.03)	0.501
rs2958905	52863768	A/G	0.19	0.0152	0.92 (0.86,0.98)	0.0484	0.92 (0.85,1)	0.17	0.93 (0.83,1.03)	0.867
rs11650051	52944139	G/A	0.13	0.0167	1.1 (1.02,1.18)	0.311	1.05 (0.96,1.15)	0.0161	1.16 (1.03,1.3)	0.0911
rs2628314	53235182	A/G	0.44	0.0175	0.94 (0.89,0.99)	0.00107	0.9 (0.85,0.96)	0.245	0.95 (0.88,1.03)	0.123
rs7503456	53280553	G/A	0.49	0.0198	0.94 (0.89,0.99)	0.000273	0.89 (0.84,0.95)	0.035	0.92 (0.85,0.99)	0.428
rs244298	53241387	G/A	0.44	0.0201	0.94 (0.89,0.99)	0.00111	0.9 (0.85,0.96)	0.285	0.96 (0.88,1.04)	0.101
rs244303	53238728	G/A	0.44	0.0202	0.94 (0.89,0.99)	0.00124	0.9 (0.85,0.96)	0.278	0.96 (0.88,1.04)	0.114
rs10432032	53224088	A/G	0.3	0.0203	0.94 (0.88,0.99)	0.0197	0.92 (0.86,0.99)	0.202	0.94 (0.86,1.03)	0.565
rs244300	53239240	T/A	0.44	0.0213	0.94 (0.9,0.99)	0.00126	0.9 (0.85,0.96)	0.288	0.96 (0.88,1.04)	0.109
rs244291	53231137	G/A	0.44	0.0222	0.94 (0.9,0.99)	0.00111	0.9 (0.85,0.96)	0.314	0.96 (0.89,1.04)	0.0895
rs244293	53230722	A/G	0.44	0.0225	0.94 (0.9,0.99)	0.00105	0.9 (0.85,0.96)	0.284	0.96 (0.88,1.04)	0.0993
rs244299	53240266	C/A	0.44	0.0225	0.94 (0.9,0.99)	0.00124	0.9 (0.85,0.96)	0.289	0.96 (0.88,1.04)	0.106
rs244320	53222920	G/A	0.3	0.0233	0.94 (0.89,0.99)	0.0194	0.92 (0.86,0.99)	0.312	0.96 (0.88,1.04)	0.424
rs244319	53222874	G/A	0.3	0.0263	0.94 (0.89,0.99)	0.022	0.92 (0.86,0.99)	0.309	0.96 (0.88,1.04)	0.452
rs2541235	53157394	G/A	0.41	0.0303	1.06 (1.01,1.11)	0.278	1.04 (0.97,1.1)	0.00705	1.12 (1.03,1.21)	0.136
rs244301	53239140	G/A	0.46	0.032	0.95 (0.9,1)	0.00177	0.91 (0.85,0.96)	0.518	0.97 (0.9,1.05)	0.0602
rs11079139	52960751	A/G	0.15	0.0341	0.93 (0.86,0.99)	0.192	0.94 (0.87,1.03)	0.426	0.96 (0.86,1.07)	0.683
rs12449786	53085570	C/A	0.42	0.0379	1.06 (1,1.11)	0.311	1.03 (0.97,1.1)	0.00973	1.11 (1.03,1.21)	0.168
rs7215402	52963855	A/G	0.1	0.0434	0.91 (0.84,1)	0.0796	0.91 (0.82,1.01)	0.513	0.96 (0.84,1.09)	0.393

rs7207239	52942456	G/A	0.15	0.044	1.08 (1,1.15)	0.448	1.03 (0.95,1.13)	0.0996	1.1 (0.98,1.23)	0.229
rs7225949	53096771	G/A	0.45	0.0445	0.95 (0.9,1)	0.00299	0.91 (0.86,0.97)	0.345	0.96 (0.89,1.04)	0.128
rs244288	53231841	G/A	0.46	0.0455	0.95 (0.9,1)	0.00393	0.91 (0.86,0.97)	0.567	0.98 (0.9,1.06)	0.0776
rs244297	53241700	A/G	0.46	0.0456	0.95 (0.9,1)	0.00279	0.91 (0.86,0.97)	0.6	0.98 (0.9,1.06)	0.061
rs7207731	52942722	G/A	0.15	0.0458	1.07 (1,1.15)	0.458	1.03 (0.95,1.13)	0.102	1.1 (0.98,1.23)	0.229
rs17817442	53099514	A/G	0.45	0.0473	0.95 (0.9,1)	0.00273	0.91 (0.86,0.97)	0.374	0.96 (0.89,1.04)	0.117
rs244339	53200114	G/A	0.47	0.0476	0.95 (0.9,1)	0.00259	0.91 (0.86,0.97)	0.581	0.98 (0.9,1.06)	0.0516
rs2170943	53209505	C/A	0.47	0.0487	0.95 (0.9,1)	0.00308	0.91 (0.86,0.97)	0.627	0.98 (0.9,1.06)	0.0554
rs2529506	53211177	G/A	0.47	0.0511	0.95 (0.9,1)	0.00372	0.91 (0.86,0.97)	0.512	0.97 (0.9,1.05)	0.0807
rs244305	53237843	A/G	0.46	0.0541	0.95 (0.9,1)	0.00433	0.92 (0.86,0.97)	0.595	0.98 (0.9,1.06)	0.0763
rs244289	53231788	A/G	0.46	0.0544	0.95 (0.9,1)	0.00446	0.92 (0.86,0.97)	0.593	0.98 (0.9,1.06)	0.0783
rs244295	53242899	T/A	0.46	0.0548	0.95 (0.9,1)	0.00433	0.92 (0.86,0.97)	0.626	0.98 (0.9,1.06)	0.0708
rs244296	53242878	A/G	0.46	0.0548	0.95 (0.9,1)	0.00424	0.91 (0.86,0.97)	0.632	0.98 (0.91,1.06)	0.0689
rs183618	53235974	A/G	0.46	0.0561	0.95 (0.91,1)	0.00463	0.92 (0.86,0.97)	0.6	0.98 (0.9,1.06)	0.0783
ch17_pos52942053	52942053	A/G	0.14	0.0581	1.07 (1,1.15)	0.432	1.04 (0.95,1.13)	0.182	1.08 (0.96,1.21)	0.364
rs244308	53212053	A/G	0.46	0.0633	0.95 (0.91,1)	0.00409	0.91 (0.86,0.97)	0.597	0.98 (0.9,1.06)	0.0661
rs2628300	53160154	G/A	0.42	0.0652	1.05 (1,1.1)	0.44	1.02 (0.96,1.09)	0.0119	1.11 (1.02,1.21)	0.139
rs244292	53230734	A/G	0.46	0.0677	0.95 (0.91,1)	0.0048	0.92 (0.86,0.97)	0.646	0.98 (0.91,1.06)	0.0701
rs244309	53212093	C/G	0.47	0.0682	0.95 (0.91,1)	0.00462	0.92 (0.86,0.97)	0.603	0.98 (0.9,1.06)	0.0698
rs244313	53214332	G/A	0.46	0.0704	0.95 (0.91,1)	0.00485	0.92 (0.86,0.97)	0.613	0.98 (0.9,1.06)	0.069
rs9891263	52937108	G/A	0.38	0.0711	1.05 (1,1.1)	0.197	1.04 (0.98,1.11)	0.53	1.03 (0.95,1.12)	0.711
rs12941961	52958586	G/A	0.09	0.0715	0.92 (0.85,1.01)	0.119	0.92 (0.83,1.02)	0.524	0.96 (0.84,1.09)	0.475
rs244311	53212803	G/A	0.47	0.0773	0.96 (0.91,1)	0.00595	0.92 (0.86,0.98)	0.604	0.98 (0.9,1.06)	0.0799
rs171510	53211527	G/A	0.47	0.0777	0.96 (0.91,1.01)	0.00576	0.92 (0.86,0.98)	0.598	0.98 (0.9,1.06)	0.0798
rs2787482	53210774	G/A	0.47	0.0805	0.96 (0.91,1.01)	0.00618	0.92 (0.86,0.98)	0.6	0.98 (0.9,1.06)	0.0823
rs244290	53231231	A/G	0.46	0.0814	0.96 (0.91,1.01)	0.00628	0.92 (0.86,0.98)	0.686	0.98 (0.91,1.07)	0.0714
rs244312	53213197	A/G	0.47	0.082	0.96 (0.91,1.01)	0.00652	0.92 (0.86,0.98)	0.597	0.98 (0.9,1.06)	0.0866
rs2628318	53210312	A/G	0.47	0.0845	0.96 (0.91,1.01)	0.00685	0.92 (0.87,0.98)	0.6	0.98 (0.9,1.06)	0.0869
rs2787488	53208151	A/G	0.47	0.0867	0.96 (0.91,1.01)	0.00644	0.92 (0.86,0.98)	0.677	0.98 (0.91,1.06)	0.0677
rs17746183	52966587	G/A	0.03	0.0927	1.14 (0.98,1.32)	0.056	1.19 (1,1.42)	0.902	0.99 (0.78,1.25)	0.148

rs244341	53198922	G/A	0.47	0.0938	0.96 (0.91,1.01)	0.00808	0.92 (0.87,0.98)	0.661	0.98 (0.91,1.06)	0.0766
rs2058026	53204759	T/A	0.47	0.0954	0.96 (0.91,1.01)	0.00762	0.92 (0.87,0.98)	0.645	0.98 (0.91,1.06)	0.0768
rs244316	53215223	G/A	0.47	0.0958	0.96 (0.91,1.01)	0.00644	0.92 (0.86,0.98)	0.727	0.99 (0.91,1.07)	0.0607
rs244350	53196132	G/C	0.47	0.0979	0.96 (0.91,1.01)	0.00808	0.92 (0.87,0.98)	0.64	0.98 (0.91,1.06)	0.0802
rs244356	53193809	C/A	0.47	0.0982	0.96 (0.91,1.01)	0.0079	0.92 (0.87,0.98)	0.676	0.98 (0.91,1.06)	0.0731
rs2787484	53210120	G/A	0.47	0.0982	0.96 (0.91,1.01)	0.00751	0.92 (0.87,0.98)	0.705	0.98 (0.91,1.07)	0.069
rs244334	53204055	A/G	0.47	0.0983	0.96 (0.91,1.01)	0.00762	0.92 (0.87,0.98)	0.667	0.98 (0.91,1.06)	0.0731
rs1994234	53229345	A/G	0.47	0.0983	0.96 (0.91,1.01)	0.00709	0.92 (0.87,0.98)	0.705	0.98 (0.91,1.07)	0.0701
rs8079105	52966403	G/A	0.22	0.0986	0.95 (0.89,1.01)	0.161	0.95 (0.88,1.02)	0.192	0.94 (0.85,1.03)	0.964
rs7212184	52942558	A/G	0.19	0.0995	1.06 (0.99,1.12)	0.269	1.05 (0.97,1.13)	0.223	1.06 (0.96,1.18)	0.559
rs17683089	52943044	C/G	0.19	0.1	1.05 (0.99,1.12)	0.261	1.05 (0.97,1.13)	0.236	1.06 (0.96,1.18)	0.589
rs244355	53194085	T/A	0.47	0.1	0.96 (0.91,1.01)	0.00845	0.92 (0.87,0.98)	0.66	0.98 (0.91,1.06)	0.0769
rs2628323	53203097	A/T	0.47	0.1	0.96 (0.91,1.01)	0.00801	0.92 (0.87,0.98)	0.667	0.98 (0.91,1.06)	0.0751
rs244307	53211713	A/G	0.47	0.101	0.96 (0.91,1.01)	0.00651	0.92 (0.86,0.98)	0.743	0.99 (0.91,1.07)	0.0576
rs244360	53191703	A/G	0.47	0.103	0.96 (0.91,1.01)	0.00805	0.92 (0.87,0.98)	0.693	0.98 (0.91,1.07)	0.0719
rs244335	53201930	C/G	0.47	0.104	0.96 (0.91,1.01)	0.00867	0.92 (0.87,0.98)	0.667	0.98 (0.91,1.06)	0.0777
rs244351	53195566	G/A	0.47	0.106	0.96 (0.91,1.01)	0.00923	0.92 (0.87,0.98)	0.679	0.98 (0.91,1.06)	0.0774
rs244368	53186537	G/A	0.47	0.107	0.96 (0.91,1.01)	0.00818	0.92 (0.87,0.98)	0.7	0.98 (0.91,1.07)	0.0737
rs2628302	53158361	G/A	0.39	0.11	1.04 (0.99,1.1)	0.556	1.02 (0.96,1.09)	0.0272	1.1 (1.01,1.19)	0.143
rs2958902	52866817	G/A	0.08	0.111	0.92 (0.84,1.02)	0.0906	0.9 (0.8,1.02)	0.685	0.97 (0.83,1.13)	0.502
rs244343	53197612	A/G	0.47	0.112	0.96 (0.91,1.01)	0.00974	0.92 (0.87,0.98)	0.68	0.98 (0.91,1.06)	0.0799
rs244344	53196998	A/G	0.47	0.113	0.96 (0.91,1.01)	0.0112	0.92 (0.87,0.98)	0.668	0.98 (0.91,1.06)	0.0897
rs244340	53199345	G/A	0.47	0.114	0.96 (0.91,1.01)	0.00919	0.92 (0.87,0.98)	0.708	0.98 (0.91,1.07)	0.0726
rs244354	53194142	G/A	0.47	0.116	0.96 (0.91,1.01)	0.0085	0.92 (0.87,0.98)	0.785	0.99 (0.91,1.07)	0.0569
rs244352	53195478	A/G	0.47	0.117	0.96 (0.91,1.01)	0.00862	0.92 (0.87,0.98)	0.767	0.99 (0.91,1.07)	0.0598
rs244333	53204178	G/A	0.47	0.117	0.96 (0.91,1.01)	0.00899	0.92 (0.87,0.98)	0.772	0.99 (0.91,1.07)	0.061
rs12603955	52996688	G/A	0.11	0.119	0.94 (0.86,1.02)	0.156	0.93 (0.84,1.03)	0.483	0.95 (0.84,1.09)	0.693
rs8065212	53024240	A/G	0.46	0.119	0.96 (0.91,1.01)	0.746	0.99 (0.93,1.05)	0.134	0.94 (0.87,1.02)	0.327
rs8080876	53175267	G/A	0.46	0.12	0.96 (0.91,1.01)	0.00923	0.92 (0.87,0.98)	0.693	0.98 (0.91,1.07)	0.0819
rs244361	53191356	A/G	0.47	0.12	0.96 (0.91,1.01)	0.00884	0.92 (0.87,0.98)	0.791	0.99 (0.91,1.07)	0.0583

rs171513	53190426	A/G	0.47	0.121	0.96 (0.91,1.01)	0.009	0.92 (0.87,0.98)	0.811	0.99 (0.91,1.07)	0.0558
rs10515083	52964057	T/A	0.25	0.123	0.95 (0.9,1.01)	0.0784	0.94 (0.87,1.01)	0.634	0.98 (0.89,1.07)	0.408
rs8072090	53174434	T/A	0.46	0.123	0.96 (0.91,1.01)	0.00923	0.92 (0.87,0.98)	0.665	0.98 (0.91,1.06)	0.0975
rs244345	53196977	T/A	0.47	0.124	0.96 (0.91,1.01)	0.00955	0.92 (0.87,0.98)	0.781	0.99 (0.91,1.07)	0.0615
rs2541241	53072993	C/A	0.47	0.126	0.96 (0.91,1.01)	0.0102	0.92 (0.87,0.98)	0.717	0.99 (0.91,1.07)	0.0894
rs244357	53193181	A/G	0.47	0.126	0.96 (0.91,1.01)	0.00953	0.92 (0.87,0.98)	0.784	0.99 (0.91,1.07)	0.061
rs2958898	52869248	C/A	0.25	0.127	1.05 (0.99,1.11)	0.471	1.03 (0.96,1.1)	0.117	1.08 (0.98,1.18)	0.192
rs7224560	53176539	G/C	0.47	0.127	0.96 (0.91,1.01)	0.0103	0.92 (0.87,0.98)	0.721	0.99 (0.91,1.07)	0.0781
rs7209029	53173046	G/A	0.46	0.128	0.96 (0.91,1.01)	0.00911	0.92 (0.87,0.98)	0.711	0.99 (0.91,1.07)	0.0856
rs2628324	53202977	A/T	0.47	0.129	0.96 (0.91,1.01)	0.00971	0.92 (0.87,0.98)	0.804	0.99 (0.91,1.07)	0.0597
rs11655949	53169883	G/A	0.46	0.13	0.96 (0.91,1.01)	0.00869	0.92 (0.87,0.98)	0.731	0.99 (0.91,1.07)	0.0828
rs4794552	53087132	T/A	0.49	0.131	0.96 (0.91,1.01)	0.856	0.99 (0.93,1.06)	0.12	0.94 (0.86,1.02)	0.22
rs244363	53188893	G/A	0.47	0.133	0.96 (0.92,1.01)	0.0103	0.92 (0.87,0.98)	0.791	0.99 (0.91,1.07)	0.0648
rs2958925	52960715	A/G	0.33	0.136	1.04 (0.99,1.1)	0.243	1.04 (0.97,1.11)	0.637	1.02 (0.94,1.11)	0.847
rs244364	53188280	G/A	0.47	0.136	0.96 (0.92,1.01)	0.00989	0.92 (0.87,0.98)	0.837	0.99 (0.92,1.07)	0.0574
rs2934892	52933304	A/C	0.14	0.137	1.06 (0.98,1.13)	0.983	1 (0.91,1.09)	0.0849	1.1 (0.99,1.24)	0.093
rs12150025	52932154	T/A	0.14	0.138	1.06 (0.98,1.13)	0.994	1 (0.91,1.09)	0.0958	1.1 (0.98,1.23)	0.116
rs9914596	52996551	G/A	0.11	0.14	1.06 (0.98,1.15)	0.648	1.02 (0.93,1.13)	0.619	1.03 (0.91,1.17)	0.837
rs9303359	52934629	A/G	0.26	0.14	1.04 (0.99,1.11)	0.662	1.02 (0.95,1.09)	0.0602	1.09 (1,1.19)	0.159
rs2787504	53178341	G/A	0.46	0.14	0.96 (0.92,1.01)	0.0114	0.92 (0.87,0.98)	0.75	0.99 (0.91,1.07)	0.077
rs2332311	52957444	A/G	0.49	0.144	1.04 (0.99,1.09)	0.424	1.03 (0.96,1.09)	0.667	1.02 (0.94,1.1)	0.92
ch17_pos52969673	52969673	G/A	0.14	0.145	0.95 (0.88,1.02)	0.034	0.91 (0.83,0.99)	0.435	1.05 (0.93,1.18)	0.0209
rs11079153	53176023	G/C	0.46	0.146	0.96 (0.92,1.01)	0.0114	0.92 (0.87,0.98)	0.773	0.99 (0.91,1.07)	0.0737
rs1948000	53147716	C/A	0.47	0.146	0.96 (0.92,1.01)	0.0128	0.93 (0.87,0.98)	0.701	0.98 (0.91,1.07)	0.108
rs2628326	53178283	G/A	0.47	0.148	0.96 (0.92,1.01)	0.0115	0.92 (0.87,0.98)	0.848	0.99 (0.92,1.07)	0.0593
rs12453615	53177509	A/G	0.47	0.15	0.96 (0.92,1.01)	0.0113	0.92 (0.87,0.98)	0.85	0.99 (0.92,1.07)	0.0608
rs8068337	53177899	G/A	0.47	0.151	0.96 (0.92,1.01)	0.0119	0.93 (0.87,0.98)	0.842	0.99 (0.92,1.07)	0.0617
rs7207057	53172968	G/C	0.47	0.157	0.96 (0.92,1.01)	0.0119	0.93 (0.87,0.98)	0.767	0.99 (0.91,1.07)	0.0922
rs8080157	53208450	A/G	0.24	0.158	1.04 (0.98,1.11)	0.138	1.06 (0.98,1.13)	0.257	1.06 (0.96,1.16)	0.631
rs12453779	53172109	A/G	0.46	0.158	0.96 (0.92,1.01)	0.0112	0.92 (0.87,0.98)	0.706	0.98 (0.91,1.07)	0.102

rs2934894	52940581	A/C	0.09	0.159	0.94 (0.86,1.03)	0.0046	0.85 (0.76,0.95)	0.5	1.05 (0.91,1.2)	0.00918
rs9303362	53062553	G/A	0.47	0.165	0.96 (0.92,1.01)	0.013	0.93 (0.87,0.98)	0.797	0.99 (0.91,1.07)	0.0859
rs2907643	52886167	A/G	0.04	0.166	0.91 (0.79,1.04)	0.281	0.91 (0.76,1.08)	0.679	0.95 (0.77,1.19)	0.66
rs2934891	52933244	A/C	0.11	0.174	0.95 (0.87,1.03)	0.0478	0.9 (0.82,1)	0.582	1.04 (0.91,1.17)	0.042
rs6504933	52828898	A/G	0.21	0.175	1.04 (0.98,1.11)	0.446	1.03 (0.96,1.11)	0.4	1.04 (0.95,1.15)	0.904
rs12450607	53127255	C/G	0.48	0.175	0.97 (0.92,1.02)	0.024	0.93 (0.88,0.99)	0.646	0.98 (0.91,1.06)	0.176
rs7218719	53245227	A/G	0.11	0.178	0.94 (0.87,1.03)	0.0607	0.91 (0.82,1)	0.0793	0.89 (0.78,1.02)	0.717
rs2907631	52867544	A/C	0.26	0.178	1.04 (0.98,1.1)	0.489	1.02 (0.96,1.1)	0.212	1.06 (0.97,1.16)	0.316
rs1860457	52868303	A/G	0.26	0.18	1.04 (0.98,1.1)	0.537	1.02 (0.95,1.1)	0.185	1.06 (0.97,1.16)	0.245
rs12937006	53030461	G/A	0.11	0.181	1.06 (0.98,1.14)	0.741	1.02 (0.92,1.12)	0.825	1.01 (0.9,1.15)	0.927
rs12453699	53171846	A/C	0.47	0.181	0.97 (0.92,1.02)	0.013	0.93 (0.87,0.98)	0.817	0.99 (0.91,1.07)	0.0832
rs10852971	53058738	G/A	0.24	0.184	1.04 (0.98,1.1)	0.17	1.05 (0.98,1.13)	0.102	1.08 (0.98,1.19)	0.888
rs6504934	52830106	G/A	0.14	0.187	1.05 (0.98,1.14)	0.306	1.05 (0.96,1.16)	0.16	1.09 (0.97,1.24)	0.566
rs1106602	52916757	C/G	0.16	0.187	1.05 (0.98,1.12)	0.364	1.04 (0.96,1.13)	0.584	1.03 (0.93,1.15)	0.818
rs2958940	52950183	A/G	0.09	0.189	0.94 (0.86,1.03)	0.011	0.87 (0.78,0.97)	0.579	1.04 (0.91,1.19)	0.017
rs2958908	52846363	A/G	0.05	0.198	0.92 (0.82,1.04)	0.178	0.9 (0.78,1.05)	0.941	0.99 (0.82,1.2)	0.427
rs6504942	52958987	G/C	0.45	0.199	0.97 (0.92,1.02)	0.28	0.97 (0.91,1.03)	0.813	0.99 (0.91,1.07)	0.65
rs8072453	52956033	G/C	0.17	0.2	0.96 (0.89,1.02)	0.135	0.94 (0.86,1.02)	0.441	0.96 (0.86,1.07)	0.835
rs7221313	52957823	T/A	0.43	0.205	0.97 (0.92,1.02)	0.188	0.96 (0.9,1.02)	0.799	1.01 (0.93,1.1)	0.299
rs7225247	52933176	G/A	0.39	0.208	1.03 (0.98,1.09)	0.535	1.02 (0.96,1.09)	0.528	1.03 (0.95,1.11)	0.916
rs974588	52864264	C/A	0.01	0.21	0.86 (0.67,1.09)	0.396	0.88 (0.64,1.19)	0.345	0.84 (0.57,1.22)	0.703
rs2934928	52956487	A/G	0.33	0.212	1.03 (0.98,1.09)	0.793	1.01 (0.94,1.08)	0.706	1.02 (0.93,1.11)	0.832
rs12945137	53065846	G/A	0.24	0.213	1.04 (0.98,1.1)	0.197	1.05 (0.98,1.13)	0.107	1.08 (0.98,1.19)	0.871
ch17_pos52846490	52846490	G/A	0.05	0.224	0.93 (0.82,1.05)	0.172	0.9 (0.77,1.05)	0.959	1 (0.83,1.21)	0.337
rs11079135	52919409	A/G	0.03	0.237	0.91 (0.79,1.06)	0.506	0.94 (0.78,1.13)	0.544	0.93 (0.73,1.18)	0.96
rs1106601	52916671	G/C	0.16	0.238	1.04 (0.97,1.12)	0.393	1.04 (0.95,1.13)	0.736	1.02 (0.91,1.13)	0.997
rs2934929	52913199	T/A	0.16	0.238	1.04 (0.97,1.12)	0.365	1.04 (0.96,1.13)	0.822	1.01 (0.91,1.13)	0.963
rs2332308	52944373	A/G	0.34	0.242	1.03 (0.98,1.09)	0.745	0.99 (0.93,1.06)	0.0816	1.08 (0.99,1.17)	0.0639
rs7211787	53234912	T/A	0.1	0.244	0.95 (0.87,1.04)	0.0579	0.9 (0.81,1)	0.124	0.9 (0.79,1.03)	0.988
rs11657590	52864887	G/A	0.27	0.245	1.03 (0.98,1.09)	0.514	1.02 (0.95,1.1)	0.262	1.05 (0.96,1.15)	0.323

rs12951542	53041965	A/G	0.11	0.248	1.05 (0.97,1.13)	0.868	1.01 (0.92,1.11)	0.997	1 (0.88,1.13)	0.984
rs2958919	52969601	A/G	0.22	0.25	1.04 (0.98,1.1)	0.792	1.01 (0.94,1.09)	0.317	1.05 (0.96,1.15)	0.313
rs12938118	53037359	A/G	0.11	0.251	1.05 (0.97,1.13)	0.867	1.01 (0.92,1.11)	0.997	1 (0.88,1.13)	0.984
rs17745231	53036529	C/G	0.11	0.251	1.05 (0.97,1.13)	0.867	1.01 (0.92,1.11)	0.997	1 (0.88,1.13)	0.984
rs1802212	53038654	A/C	0.11	0.251	1.05 (0.97,1.13)	0.867	1.01 (0.92,1.11)	0.997	1 (0.88,1.13)	0.984
rs9898886	53249679	A/G	0.09	0.254	0.95 (0.87,1.04)	0.0723	0.91 (0.81,1.01)	0.134	0.9 (0.78,1.03)	0.893
rs2332314	53033007	A/C	0.24	0.261	1.04 (0.97,1.1)	0.748	0.99 (0.92,1.06)	0.963	1 (0.91,1.1)	0.567
rs17745049	52935126	G/A	0.04	0.263	0.93 (0.82,1.06)	0.882	0.99 (0.85,1.15)	0.399	0.92 (0.75,1.12)	0.59
rs3087650	53029328	G/A	0.11	0.266	1.05 (0.97,1.13)	0.881	1.01 (0.91,1.11)	0.96	1 (0.88,1.13)	0.984
rs12150038	53041520	A/C	0.11	0.266	1.05 (0.97,1.13)	0.91	1.01 (0.91,1.11)	0.997	1 (0.88,1.13)	0.951
rs12936639	53235982	G/A	0.1	0.27	0.95 (0.87,1.04)	0.0619	0.9 (0.81,1.01)	0.137	0.9 (0.79,1.03)	0.967
rs8066588	53025689	G/A	0.11	0.27	1.05 (0.97,1.13)	0.928	1 (0.91,1.11)	0.988	1 (0.88,1.13)	0.914
rs12936860	53030226	G/A	0.11	0.275	1.04 (0.97,1.13)	0.933	1 (0.91,1.11)	0.994	1 (0.88,1.13)	0.932
rs8072720	52955672	A/T	0.43	0.278	0.97 (0.92,1.02)	0.309	0.97 (0.91,1.03)	0.268	0.96 (0.88,1.04)	0.99
rs2907636	52879449	A/G	0.16	0.28	1.04 (0.97,1.11)	0.348	1.04 (0.96,1.13)	0.674	1.02 (0.92,1.14)	0.892
rs2907641	52883339	G/A	0.15	0.283	1.04 (0.97,1.11)	0.405	1.04 (0.95,1.13)	0.646	1.03 (0.92,1.14)	0.802
rs16955216	52929254	A/C	0.48	0.298	1.03 (0.98,1.08)	0.889	1 (0.94,1.06)	0.182	1.06 (0.97,1.14)	0.169
rs12937760	53016130	C/A	0.12	0.317	1.04 (0.96,1.12)	0.933	1 (0.91,1.09)	0.847	1.01 (0.9,1.14)	0.731
rs2332265	52830795	T/A	0.35	0.318	1.03 (0.97,1.08)	0.686	1.01 (0.95,1.08)	0.194	1.06 (0.97,1.15)	0.395
rs7226272	53046447	A/G	0.43	0.319	1.03 (0.98,1.08)	0.881	1 (0.94,1.07)	0.0815	1.08 (0.99,1.17)	0.21
rs12951898	53035602	G/A	0.23	0.323	1.03 (0.97,1.1)	0.648	0.98 (0.91,1.06)	0.901	0.99 (0.9,1.1)	0.593
rs4793783	53025480	A/G	0.23	0.329	1.03 (0.97,1.1)	0.596	0.98 (0.91,1.06)	0.901	0.99 (0.9,1.1)	0.546
rs12944690	53029614	A/G	0.23	0.334	1.03 (0.97,1.1)	0.638	0.98 (0.91,1.06)	0.891	0.99 (0.9,1.09)	0.591
rs12165058	53022049	A/G	0.23	0.335	1.03 (0.97,1.09)	0.586	0.98 (0.91,1.06)	0.898	0.99 (0.9,1.09)	0.541
rs7224432	53176480	C/A	0.24	0.337	1.03 (0.97,1.09)	0.272	1.04 (0.97,1.12)	0.278	1.05 (0.96,1.16)	0.794
rs7208403	53046667	A/C	0.43	0.346	1.02 (0.97,1.08)	0.931	1 (0.94,1.07)	0.0857	1.07 (0.99,1.17)	0.201
rs12946841	52971277	C/A	0.08	0.354	1.05 (0.95,1.15)	0.852	1.01 (0.9,1.13)	0.871	0.99 (0.85,1.15)	0.934
rs2934890	52931473	G/A	0.07	0.356	0.95 (0.86,1.05)	0.0469	0.88 (0.78,1)	0.522	1.05 (0.9,1.23)	0.0301
rs6504947	53004144	A/T	0.41	0.36	0.98 (0.93,1.03)	0.0571	0.94 (0.88,1)	0.753	0.99 (0.91,1.07)	0.173
rs1008523	53263614	C/G	0.3	0.367	1.03 (0.97,1.08)	0.024	1.08 (1.01,1.15)	0.272	0.95 (0.87,1.04)	0.00859

rs12952166	53005586	G/A	0.41	0.369	0.98 (0.93,1.03)	0.0634	0.94 (0.89,1)	0.696	0.98 (0.91,1.07)	0.203
rs2529527	53186402	A/G	0.16	0.376	1.03 (0.96,1.11)	0.438	0.97 (0.89,1.05)	0.203	1.07 (0.96,1.2)	0.0392
rs2628298	53163478	T/A	0.1	0.378	0.96 (0.88,1.05)	0.138	0.92 (0.83,1.03)	0.127	0.9 (0.78,1.03)	0.885
rs7212321	53258437	C/G	0.1	0.378	0.96 (0.89,1.05)	0.1	0.92 (0.83,1.02)	0.161	0.91 (0.8,1.04)	0.809
ch17_pos53018824	53018824	G/A	0.41	0.379	0.98 (0.93,1.03)	0.0533	0.94 (0.88,1)	0.712	0.98 (0.91,1.07)	0.176
rs12937360	53046298	A/G	0.42	0.379	1.02 (0.97,1.08)	0.959	1 (0.94,1.07)	0.117	1.07 (0.98,1.16)	0.233
rs7214573	53006654	A/G	0.12	0.38	1.04 (0.96,1.12)	0.902	0.99 (0.9,1.09)	0.923	0.99 (0.88,1.12)	0.902
rs244329	53267230	A/G	0.3	0.38	1.02 (0.97,1.08)	0.0221	1.08 (1.01,1.15)	0.251	0.95 (0.87,1.04)	0.00696
rs2934884	52918902	A/G	0.28	0.381	1.03 (0.97,1.08)	0.163	0.95 (0.89,1.02)	0.0316	1.1 (1.01,1.2)	0.00277
rs17745189	53013980	A/G	0.12	0.394	1.03 (0.96,1.12)	0.937	1 (0.91,1.1)	0.873	0.99 (0.88,1.12)	0.972
rs8066213	52930323	A/C	0.47	0.396	1.02 (0.97,1.08)	0.717	0.99 (0.93,1.05)	0.234	1.05 (0.97,1.14)	0.171
rs1601297	53253019	A/G	0.3	0.398	1.02 (0.97,1.08)	0.025	1.08 (1.01,1.15)	0.247	0.95 (0.87,1.04)	0.00782
rs12949538	53010670	G/A	0.12	0.399	1.03 (0.96,1.12)	0.838	0.99 (0.9,1.09)	0.937	1 (0.88,1.12)	0.841
rs2958900	52868658	A/G	0.18	0.413	1.03 (0.96,1.1)	0.704	1.02 (0.94,1.1)	0.441	1.04 (0.94,1.15)	0.372
rs7218098	52834303	A/G	0.21	0.415	1.03 (0.96,1.09)	0.689	1.02 (0.94,1.1)	0.317	1.05 (0.95,1.16)	0.645
rs2643023	53052002	G/A	0.33	0.417	1.02 (0.97,1.08)	0.563	1.02 (0.95,1.09)	0.63	1.02 (0.94,1.11)	0.86
rs2332303	52969844	A/G	0.47	0.417	1.02 (0.97,1.07)	0.125	1.05 (0.99,1.11)	0.96	1 (0.92,1.08)	0.154
rs11869357	53193721	A/G	0.17	0.428	1.03 (0.96,1.1)	0.42	0.97 (0.89,1.05)	0.269	1.06 (0.95,1.18)	0.0613
rs11079129	52869546	G/A	0.36	0.429	1.02 (0.97,1.08)	0.894	1 (0.93,1.06)	0.106	1.07 (0.99,1.16)	0.0552
ch17_pos52831447	52831447	G/A	0.01	0.433	1.14 (0.82,1.58)	0.354	0.81 (0.53,1.26)	0.0038	1.91 (1.25,2.92)	0.00692
rs17675596	52872714	G/C	0.11	0.433	0.97 (0.89,1.05)	0.981	1 (0.9,1.1)	0.0862	0.89 (0.78,1.02)	0.0737
rs9915913	52998168	C/A	0.12	0.435	1.03 (0.95,1.11)	0.729	0.98 (0.89,1.08)	0.924	0.99 (0.88,1.12)	0.761
rs9904768	53270820	A/C	0.24	0.436	1.02 (0.97,1.09)	0.459	1.03 (0.96,1.1)	0.129	1.08 (0.98,1.18)	0.528
rs17745534	53137506	G/A	0.09	0.437	0.97 (0.88,1.05)	0.2	0.93 (0.84,1.04)	0.113	0.89 (0.78,1.03)	0.819
ch17_pos53017747	53017747	A/G	0.12	0.439	1.03 (0.95,1.11)	0.967	1 (0.91,1.1)	0.913	0.99 (0.88,1.12)	0.936
rs2628310	53255472	A/G	0.3	0.446	1.02 (0.97,1.08)	0.0301	1.08 (1.01,1.15)	0.231	0.95 (0.87,1.04)	0.00827
rs11079143	53083122	G/A	0.09	0.447	0.97 (0.89,1.06)	0.202	0.93 (0.84,1.04)	0.125	0.9 (0.78,1.03)	0.857
rs9303363	53137974	G/A	0.09	0.451	0.97 (0.89,1.06)	0.206	0.93 (0.84,1.04)	0.115	0.89 (0.78,1.03)	0.819
rs2787505	53107432	G/A	0.09	0.452	0.97 (0.89,1.06)	0.202	0.93 (0.84,1.04)	0.117	0.89 (0.78,1.03)	0.827
rs244328	53268945	G/A	0.3	0.453	1.02 (0.97,1.08)	0.0318	1.07 (1.01,1.15)	0.238	0.95 (0.87,1.04)	0.00829

rs714897	53256579	G/A	0.09	0.457	0.97 (0.89,1.06)	0.118	0.92 (0.83,1.02)	0.224	0.92 (0.8,1.05)	0.975
rs16955257	52942491	C/A	0.06	0.46	1.04 (0.93,1.16)	0.796	1.02 (0.89,1.16)	0.407	1.07 (0.91,1.27)	0.822
rs2628309	53115419	A/G	0.09	0.463	0.97 (0.89,1.06)	0.201	0.93 (0.84,1.04)	0.123	0.9 (0.78,1.03)	0.85
rs2628305	53140427	C/A	0.09	0.466	0.97 (0.89,1.06)	0.205	0.93 (0.84,1.04)	0.13	0.9 (0.78,1.03)	0.865
rs2541245	53101200	A/G	0.09	0.467	0.97 (0.89,1.06)	0.197	0.93 (0.83,1.04)	0.129	0.9 (0.78,1.03)	0.876
rs2628322	53205356	G/A	0.17	0.467	1.03 (0.96,1.1)	0.395	0.96 (0.89,1.05)	0.302	1.06 (0.95,1.18)	0.0657
ch17_pos52822633	52822633	A/C	0.06	0.469	1.04 (0.93,1.16)	0.61	1.03 (0.91,1.18)	0.195	1.12 (0.95,1.31)	0.529
ch17_pos53036109	53036109	A/G	0.25	0.471	1.02 (0.96,1.08)	0.478	0.97 (0.91,1.05)	0.899	0.99 (0.9,1.09)	0.483
rs4794540	52945104	G/A	0.49	0.474	0.98 (0.93,1.03)	0.941	1 (0.94,1.07)	0.578	0.98 (0.9,1.06)	0.493
rs2529499	53188361	G/A	0.17	0.475	1.02 (0.96,1.1)	0.365	0.96 (0.89,1.05)	0.277	1.06 (0.95,1.18)	0.0567
rs2529500	53188379	G/A	0.17	0.475	1.02 (0.96,1.1)	0.365	0.96 (0.89,1.05)	0.277	1.06 (0.95,1.18)	0.0567
rs11079128	52867748	G/A	0.33	0.476	1.02 (0.97,1.08)	0.945	1 (0.93,1.07)	0.205	1.06 (0.97,1.15)	0.139
rs11658717	53076986	A/G	0.09	0.478	0.97 (0.89,1.06)	0.209	0.93 (0.84,1.04)	0.129	0.9 (0.78,1.03)	0.85
rs2628308	53123573	G/C	0.09	0.478	0.97 (0.89,1.06)	0.209	0.93 (0.84,1.04)	0.129	0.9 (0.78,1.03)	0.85
rs2787507	53127924	A/G	0.09	0.481	0.97 (0.89,1.06)	0.208	0.93 (0.84,1.04)	0.129	0.9 (0.78,1.03)	0.85
rs12947685	53045519	A/G	0.18	0.481	0.98 (0.91,1.04)	0.0932	0.93 (0.86,1.01)	0.908	1.01 (0.91,1.12)	0.133
rs1484776	53111383	C/A	0.09	0.482	0.97 (0.89,1.06)	0.216	0.93 (0.84,1.04)	0.125	0.9 (0.78,1.03)	0.833
rs8075983	53271918	G/A	0.09	0.484	0.97 (0.89,1.06)	0.125	0.92 (0.83,1.02)	0.249	0.92 (0.8,1.06)	0.991
rs2787499	53170985	A/T	0.15	0.485	1.03 (0.96,1.1)	0.392	0.96 (0.88,1.05)	0.303	1.06 (0.95,1.18)	0.0693
ch17_pos52974643	52974643	C/G	0.2	0.49	0.98 (0.92,1.04)	0.251	0.96 (0.89,1.03)	0.263	0.94 (0.85,1.04)	0.994
rs16955267	52943855	G/C	0.48	0.491	0.98 (0.93,1.03)	0.981	1 (0.94,1.06)	0.498	0.97 (0.9,1.05)	0.476
rs16955290	52949534	T/A	0.17	0.492	0.98 (0.91,1.04)	0.773	1.01 (0.93,1.1)	0.0837	0.91 (0.81,1.01)	0.0981
rs1484770	53168340	G/A	0.15	0.493	1.02 (0.96,1.1)	0.41	0.96 (0.88,1.05)	0.336	1.06 (0.95,1.18)	0.0836
rs8072827	53073858	A/G	0.17	0.494	1.02 (0.96,1.1)	0.417	0.97 (0.89,1.05)	0.348	1.05 (0.95,1.17)	0.115
rs2541240	53042872	G/A	0.31	0.498	1.02 (0.96,1.08)	0.511	1.02 (0.96,1.09)	0.108	1.07 (0.98,1.17)	0.568
rs1156287	53076799	A/G	0.09	0.502	0.97 (0.89,1.06)	0.221	0.93 (0.84,1.04)	0.133	0.9 (0.78,1.03)	0.841
rs1484771	53168495	A/C	0.15	0.502	1.02 (0.96,1.1)	0.386	0.96 (0.88,1.05)	0.328	1.06 (0.95,1.18)	0.073
rs1962045	52996150	G/A	0.12	0.507	1.03 (0.95,1.11)	0.59	0.97 (0.89,1.07)	0.834	0.99 (0.87,1.11)	0.765
rs4372751	52986725	G/A	0.28	0.511	1.02 (0.96,1.08)	0.396	1.03 (0.96,1.1)	0.0944	1.08 (0.99,1.18)	0.771
rs35496173	52896367	C/A	0.32	0.511	1.02 (0.96,1.08)	0.87	0.99 (0.93,1.06)	0.278	1.05 (0.96,1.14)	0.165

rs2628319	53210110	G/A	0.17	0.513	1.02 (0.96,1.09)	0.355	0.96 (0.88,1.05)	0.29	1.06 (0.95,1.18)	0.0577
rs9910653	52992268	G/A	0.23	0.513	0.98 (0.92,1.04)	0.158	0.95 (0.88,1.02)	0.45	0.96 (0.88,1.06)	0.681
rs17817950	53045325	G/A	0.18	0.516	0.98 (0.92,1.04)	0.101	0.93 (0.86,1.01)	0.848	1.01 (0.91,1.12)	0.124
rs9899545	52986845	A/G	0.13	0.519	0.98 (0.91,1.05)	0.0649	0.92 (0.84,1.01)	0.215	0.93 (0.83,1.04)	0.67
rs2787495	53159626	G/C	0.15	0.52	1.02 (0.95,1.1)	0.39	0.96 (0.88,1.05)	0.336	1.06 (0.95,1.18)	0.0848
rs8070668	52991636	A/G	0.31	0.525	1.02 (0.96,1.08)	0.46	1.03 (0.96,1.1)	0.0909	1.08 (0.99,1.17)	0.61
rs9895901	52984885	A/G	0.25	0.53	0.98 (0.93,1.04)	0.138	0.95 (0.88,1.02)	0.325	0.95 (0.87,1.05)	0.633
rs17635420	52833331	A/C	0.17	0.536	1.02 (0.95,1.09)	0.514	1.03 (0.95,1.12)	0.712	0.98 (0.88,1.09)	0.426
rs7216138	52995664	G/A	0.23	0.536	0.98 (0.92,1.04)	0.154	0.95 (0.88,1.02)	0.49	0.97 (0.88,1.06)	0.637
rs12602751	52994174	C/A	0.23	0.54	0.98 (0.92,1.04)	0.157	0.95 (0.88,1.02)	0.512	0.97 (0.88,1.07)	0.609
rs2111098	53174171	G/A	0.16	0.542	1.02 (0.95,1.1)	0.37	0.96 (0.88,1.05)	0.343	1.05 (0.94,1.18)	0.0753
rs1026961	53155666	G/A	0.15	0.544	1.02 (0.95,1.1)	0.344	0.96 (0.88,1.05)	0.344	1.05 (0.94,1.18)	0.0729
rs2958907	52850855	A/G	0.44	0.547	0.98 (0.94,1.04)	0.742	1.01 (0.95,1.07)	0.0153	0.9 (0.83,0.98)	0.00858
rs17818040	53083891	G/A	0.17	0.548	1.02 (0.95,1.09)	0.391	0.96 (0.89,1.05)	0.353	1.05 (0.95,1.17)	0.115
rs9904377	52985982	G/A	0.25	0.556	0.98 (0.93,1.04)	0.156	0.95 (0.88,1.02)	0.352	0.96 (0.87,1.05)	0.641
rs35079296	52994210	A/C	0.12	0.557	1.02 (0.95,1.1)	0.618	0.98 (0.89,1.07)	0.736	0.98 (0.87,1.1)	0.862
rs2787481	53211110	A/G	0.1	0.559	0.98 (0.9,1.06)	0.178	0.93 (0.84,1.03)	0.151	0.91 (0.79,1.04)	0.857
rs2958914	52975937	A/G	0.26	0.559	0.98 (0.93,1.04)	0.533	0.98 (0.91,1.05)	0.642	0.98 (0.89,1.07)	0.755
rs7222890	52989060	T/A	0.28	0.566	1.02 (0.96,1.08)	0.457	1.03 (0.96,1.1)	0.0982	1.08 (0.99,1.18)	0.725
rs2958913	52976942	G/C	0.38	0.567	1.02 (0.96,1.07)	0.747	0.99 (0.93,1.05)	0.455	1.03 (0.95,1.12)	0.599
rs9914088	52979985	A/G	0.25	0.568	0.98 (0.93,1.04)	0.147	0.95 (0.88,1.02)	0.346	0.96 (0.87,1.05)	0.618
ch17_pos53120203	53120203	A/G	0.17	0.569	1.02 (0.95,1.09)	0.361	0.96 (0.88,1.05)	0.409	1.05 (0.94,1.17)	0.118
rs2787506	53110072	A/G	0.17	0.573	1.02 (0.95,1.09)	0.358	0.96 (0.88,1.05)	0.41	1.05 (0.94,1.17)	0.118
rs2787475	53215644	A/G	0.17	0.574	1.02 (0.95,1.09)	0.312	0.96 (0.88,1.04)	0.334	1.05 (0.95,1.17)	0.0619
rs17745091	52938797	G/A	0.34	0.574	1.02 (0.96,1.07)	0.238	0.96 (0.9,1.03)	0.083	1.08 (0.99,1.17)	0.0176
rs9908752	52983216	G/A	0.25	0.577	0.98 (0.93,1.04)	0.152	0.95 (0.88,1.02)	0.35	0.96 (0.87,1.05)	0.61
rs244317	53216985	T/A	0.1	0.578	0.98 (0.9,1.06)	0.185	0.93 (0.84,1.03)	0.156	0.91 (0.79,1.04)	0.857
rs2541244	53100168	A/G	0.17	0.58	1.02 (0.95,1.09)	0.332	0.96 (0.88,1.04)	0.383	1.05 (0.94,1.17)	0.0961
rs1933325	53148136	G/A	0.17	0.584	1.02 (0.95,1.09)	0.351	0.96 (0.88,1.05)	0.414	1.05 (0.94,1.17)	0.118
rs12452447	52964587	C/A	0.05	0.585	0.97 (0.86,1.09)	0.66	1.03 (0.89,1.2)	0.569	0.95 (0.78,1.15)	0.501

rs17818058	53084124	A/G	0.17	0.585	1.02 (0.95,1.09)	0.35	0.96 (0.88,1.05)	0.418	1.05 (0.94,1.17)	0.118
rs17745391	53071872	A/G	0.17	0.586	1.02 (0.95,1.09)	0.365	0.96 (0.88,1.05)	0.395	1.05 (0.94,1.17)	0.112
rs9899602	52986908	A/G	0.13	0.588	0.98 (0.91,1.06)	0.0732	0.92 (0.84,1.01)	0.235	0.93 (0.83,1.05)	0.655
rs2628301	53159273	G/A	0.09	0.593	0.98 (0.89,1.07)	0.19	0.93 (0.83,1.04)	0.215	0.92 (0.8,1.05)	0.968
rs9897646	52985351	G/C	0.13	0.594	0.98 (0.91,1.06)	0.0694	0.92 (0.84,1.01)	0.239	0.93 (0.83,1.05)	0.644
rs1351888	53149239	G/A	0.17	0.596	1.02 (0.95,1.09)	0.342	0.96 (0.88,1.04)	0.425	1.05 (0.94,1.16)	0.118
rs17683844	52965694	A/G	0.16	0.598	0.98 (0.91,1.05)	0.288	0.96 (0.88,1.04)	0.186	0.93 (0.83,1.04)	0.608
rs244315	53214654	G/A	0.1	0.599	0.98 (0.9,1.06)	0.182	0.93 (0.84,1.03)	0.173	0.91 (0.79,1.04)	0.893
rs1564821	53248927	G/A	0.12	0.599	0.98 (0.91,1.06)	0.0873	0.92 (0.83,1.01)	0.302	1.07 (0.94,1.2)	0.012
rs17745201	53027561	A/T	0.05	0.6	1.03 (0.92,1.16)	0.47	0.95 (0.82,1.09)	0.812	0.98 (0.81,1.17)	0.652
rs4436838	52947714	A/C	0.17	0.601	0.98 (0.92,1.05)	0.694	1.02 (0.94,1.1)	0.116	0.92 (0.82,1.02)	0.108
rs9913784	52989071	T/A	0.25	0.601	0.98 (0.93,1.04)	0.175	0.95 (0.89,1.02)	0.33	0.96 (0.87,1.05)	0.704
rs2958921	52965793	G/A	0.26	0.601	0.98 (0.93,1.04)	0.932	1 (0.93,1.07)	0.336	0.96 (0.87,1.05)	0.496
rs2628303	53158106	G/A	0.17	0.602	1.02 (0.95,1.09)	0.346	0.96 (0.88,1.04)	0.412	1.05 (0.94,1.17)	0.109
rs12453444	52839090	A/G	0.08	0.604	1.02 (0.93,1.12)	0.444	0.96 (0.85,1.07)	0.0428	1.16 (1.01,1.34)	0.0151
rs7210248	52964283	G/A	0.21	0.605	0.98 (0.92,1.05)	0.664	0.98 (0.91,1.06)	0.707	0.98 (0.89,1.09)	0.956
rs2787497	53176211	A/G	0.1	0.611	0.98 (0.9,1.06)	0.177	0.93 (0.84,1.03)	0.194	0.91 (0.8,1.05)	0.933
rs2628299	53160946	C/A	0.17	0.613	1.02 (0.95,1.09)	0.342	0.96 (0.88,1.04)	0.453	1.04 (0.94,1.16)	0.125
rs16955774	53272003	A/G	0.17	0.615	0.98 (0.92,1.05)	0.515	0.97 (0.9,1.06)	0.904	0.99 (0.89,1.11)	0.699
rs4794530	52837689	G/A	0.31	0.623	1.01 (0.96,1.07)	0.431	1.03 (0.96,1.1)	0.9	0.99 (0.91,1.09)	0.41
rs17745123	52988232	C/A	0.13	0.626	0.98 (0.91,1.06)	0.0746	0.92 (0.84,1.01)	0.235	0.93 (0.83,1.05)	0.662
rs244302	53238871	G/A	0.14	0.63	1.02 (0.95,1.1)	0.466	0.97 (0.88,1.06)	0.239	1.07 (0.96,1.2)	0.0439
rs2787498	53172871	A/G	0.17	0.63	1.02 (0.95,1.09)	0.302	0.96 (0.88,1.04)	0.415	1.05 (0.94,1.17)	0.0966
rs9895808	53048442	C/G	0.09	0.631	0.98 (0.9,1.07)	0.262	0.94 (0.84,1.05)	0.173	0.91 (0.79,1.04)	0.832
ch17_pos52850065	52850065	T/A	0.1	0.637	1.02 (0.94,1.11)	0.516	1.03 (0.93,1.14)	0.558	0.96 (0.84,1.1)	0.219
rs11079130	52889930	G/A	0.46	0.644	1.01 (0.96,1.07)	0.785	1.01 (0.95,1.07)	0.422	1.03 (0.95,1.12)	0.485
rs244358	53192231	G/A	0.09	0.645	0.98 (0.9,1.07)	0.19	0.93 (0.83,1.04)	0.223	0.92 (0.8,1.06)	0.923
rs2958891	52881683	A/G	0.2	0.645	0.99 (0.92,1.05)	0.306	0.96 (0.89,1.04)	0.29	1.06 (0.95,1.17)	0.103
rs244304	53237959	A/G	0.14	0.648	1.02 (0.95,1.09)	0.436	0.96 (0.88,1.06)	0.246	1.07 (0.95,1.2)	0.0422
rs2332307	52944043	A/G	0.19	0.65	0.99 (0.92,1.05)	0.24	0.95 (0.88,1.03)	0.528	1.03 (0.93,1.14)	0.215

ch17_pos52881093	52881093	A/G	0.04	0.651	0.97 (0.86,1.1)	0.709	0.97 (0.83,1.14)	0.454	1.08 (0.89,1.31)	0.307
rs2958915	52975892	A/C	0.14	0.656	0.98 (0.91,1.06)	0.509	0.97 (0.88,1.06)	0.576	0.97 (0.85,1.09)	0.846
rs10468513	53062903	C/A	0.09	0.66	0.98 (0.9,1.07)	0.299	0.94 (0.85,1.05)	0.163	0.91 (0.79,1.04)	0.75
rs7212889	52834061	C/A	0.16	0.66	1.02 (0.95,1.09)	0.486	1.03 (0.95,1.12)	0.541	0.97 (0.86,1.08)	0.305
rs2628296	53166495	T/A	0.1	0.661	0.98 (0.9,1.07)	0.212	0.94 (0.84,1.04)	0.229	0.92 (0.8,1.05)	0.943
rs1990674	53061075	G/A	0.09	0.662	0.98 (0.9,1.07)	0.297	0.94 (0.85,1.05)	0.161	0.91 (0.79,1.04)	0.758
rs2958876	52904526	A/G	0.09	0.664	1.02 (0.93,1.12)	0.94	1 (0.89,1.11)	0.459	1.06 (0.92,1.22)	0.411
rs17745344	53048319	A/G	0.09	0.666	0.98 (0.9,1.07)	0.299	0.94 (0.85,1.05)	0.172	0.91 (0.79,1.04)	0.779
rs7219874	53065807	G/A	0.09	0.668	0.98 (0.9,1.07)	0.3	0.94 (0.85,1.05)	0.162	0.91 (0.79,1.04)	0.758
rs9890971	53059776	A/T	0.09	0.668	0.98 (0.9,1.07)	0.3	0.94 (0.85,1.05)	0.162	0.91 (0.79,1.04)	0.758
rs9892173	53060214	G/A	0.09	0.668	0.98 (0.9,1.07)	0.3	0.94 (0.85,1.05)	0.162	0.91 (0.79,1.04)	0.758
rs9902718	53061622	A/G	0.09	0.668	0.98 (0.9,1.07)	0.3	0.94 (0.85,1.05)	0.162	0.91 (0.79,1.04)	0.758
ch17_pos52917757	52917757	C/A	0.14	0.669	1.02 (0.94,1.09)	0.435	1.04 (0.95,1.13)	0.758	1.02 (0.9,1.15)	0.493
rs6504949	53050133	A/C	0.09	0.67	0.98 (0.9,1.07)	0.302	0.94 (0.85,1.05)	0.162	0.91 (0.79,1.04)	0.754
rs1029626	53220620	A/G	0.17	0.67	1.01 (0.95,1.09)	0.343	0.96 (0.88,1.04)	0.449	1.04 (0.94,1.16)	0.0953
rs12953288	53227444	A/G	0.17	0.67	1.01 (0.95,1.09)	0.328	0.96 (0.88,1.04)	0.47	1.04 (0.93,1.16)	0.0974
rs16954962	52816899	G/A	0.11	0.671	1.02 (0.94,1.1)	0.925	1 (0.91,1.1)	0.49	1.04 (0.92,1.18)	0.805
rs9916642	53054497	A/G	0.09	0.672	0.98 (0.9,1.07)	0.3	0.94 (0.85,1.05)	0.162	0.91 (0.79,1.04)	0.758
rs9891865	53060033	G/A	0.09	0.676	0.98 (0.9,1.07)	0.308	0.95 (0.85,1.05)	0.164	0.91 (0.79,1.04)	0.75
rs7221223	53059007	A/G	0.09	0.679	0.98 (0.9,1.07)	0.306	0.95 (0.85,1.05)	0.164	0.91 (0.79,1.04)	0.758
rs6504948	53049987	A/G	0.09	0.681	0.98 (0.9,1.07)	0.3	0.94 (0.85,1.05)	0.176	0.91 (0.79,1.04)	0.794
rs9896044	53048542	C/G	0.09	0.681	0.98 (0.9,1.07)	0.3	0.94 (0.85,1.05)	0.176	0.91 (0.79,1.04)	0.794
rs2191230	52869571	C/G	0.45	0.681	1.01 (0.96,1.06)	0.913	1 (0.94,1.06)	0.388	1.04 (0.96,1.12)	0.263
rs9914836	52997875	C/G	0.1	0.683	0.98 (0.9,1.07)	0.18	0.93 (0.84,1.03)	0.111	0.9 (0.78,1.03)	0.795
rs6504950	53056471	G/A	0.09	0.685	0.98 (0.9,1.07)	0.317	0.95 (0.85,1.05)	0.162	0.91 (0.79,1.04)	0.738
rs7219986	53263207	G/A	0.17	0.69	0.99 (0.92,1.06)	0.515	0.97 (0.9,1.06)	0.991	1 (0.9,1.11)	0.629
rs17711168	53225017	A/G	0.17	0.693	1.01 (0.95,1.08)	0.333	0.96 (0.88,1.04)	0.452	1.04 (0.94,1.16)	0.0933
rs7222197	53047499	G/A	0.09	0.694	0.98 (0.9,1.07)	0.303	0.94 (0.85,1.05)	0.178	0.91 (0.79,1.04)	0.794
rs4793786	53228869	A/C	0.17	0.698	1.01 (0.95,1.08)	0.321	0.96 (0.88,1.04)	0.443	1.04 (0.94,1.16)	0.0865
rs16954966	52818980	G/A	0.31	0.698	1.01 (0.96,1.07)	0.322	1.03 (0.97,1.1)	0.757	0.99 (0.9,1.08)	0.346

rs4794549	53046726	C/A	0.19	0.709	0.99 (0.93,1.05)	0.133	0.94 (0.87,1.02)	0.842	1.01 (0.91,1.12)	0.147
rs17708843	52838076	T/A	0.13	0.712	0.99 (0.91,1.06)	0.367	0.96 (0.87,1.05)	0.853	1.01 (0.89,1.14)	0.354
rs12938755	52920878	A/G	0.16	0.713	0.99 (0.92,1.06)	0.527	1.03 (0.94,1.12)	0.0709	0.9 (0.8,1.01)	0.0426
rs17682072	52915390	G/A	0.17	0.714	1.01 (0.95,1.08)	0.701	1.02 (0.94,1.1)	0.72	1.02 (0.92,1.13)	0.783
rs8064882	53046209	G/A	0.19	0.718	0.99 (0.93,1.05)	0.132	0.94 (0.87,1.02)	0.818	1.01 (0.91,1.12)	0.141
rs2958943	52948531	G/A	0.14	0.719	0.99 (0.92,1.06)	0.0828	0.92 (0.85,1.01)	0.3	1.06 (0.95,1.19)	0.0521
rs11657917	52918326	C/A	0.15	0.719	1.01 (0.94,1.09)	0.496	1.03 (0.95,1.12)	0.716	1.02 (0.91,1.14)	0.549
rs9907270	53266415	G/A	0.1	0.723	0.98 (0.9,1.07)	0.264	0.94 (0.85,1.05)	0.418	0.95 (0.82,1.08)	0.989
rs11653075	52873744	G/A	0.36	0.726	1.01 (0.96,1.06)	0.668	0.99 (0.92,1.05)	0.172	1.06 (0.98,1.15)	0.0614
rs244336	53201502	G/A	0.09	0.728	0.98 (0.9,1.08)	0.241	0.94 (0.84,1.05)	0.226	0.92 (0.8,1.06)	0.987
rs17745322	52942748	G/A	0.04	0.734	0.98 (0.87,1.11)	0.438	1.06 (0.92,1.22)	0.622	0.95 (0.79,1.16)	0.348
rs2958875	52906122	A/G	0.27	0.747	1.01 (0.95,1.07)	0.613	1.02 (0.95,1.09)	0.492	0.97 (0.89,1.06)	0.407
rs2934909	52948507	T/A	0.14	0.761	0.99 (0.92,1.06)	0.1	0.93 (0.85,1.01)	0.305	1.06 (0.95,1.19)	0.0584
rs2907639	52882437	A/C	0.23	0.763	1.01 (0.95,1.07)	0.811	0.99 (0.92,1.07)	0.605	1.03 (0.93,1.13)	0.386
rs16955309	52961757	G/A	0.33	0.763	0.99 (0.94,1.05)	0.776	0.99 (0.93,1.06)	0.604	1.02 (0.94,1.12)	0.667
rs2907659	52898034	G/A	0.2	0.765	0.99 (0.93,1.06)	0.35	0.96 (0.89,1.04)	0.22	1.07 (0.96,1.18)	0.0899
rs12449648	52871802	G/A	0.47	0.776	1.01 (0.96,1.06)	0.982	1 (0.94,1.07)	0.491	1.03 (0.95,1.12)	0.445
rs8076678	53284506	A/T	0.06	0.783	1.02 (0.91,1.14)	0.423	0.95 (0.82,1.08)	0.625	1.05 (0.87,1.25)	0.296
rs4793781	52962115	G/A	0.16	0.785	0.99 (0.92,1.06)	0.47	0.97 (0.89,1.06)	0.871	0.99 (0.89,1.11)	0.835
rs12936957	53058202	A/G	0.19	0.785	0.99 (0.93,1.06)	0.166	0.94 (0.87,1.02)	0.78	1.01 (0.91,1.13)	0.158
rs1008524	53263720	G/A	0.15	0.786	1.01 (0.94,1.08)	0.293	0.95 (0.87,1.04)	0.144	1.09 (0.97,1.22)	0.0161
rs2787480	53102900	G/A	0.13	0.787	0.99 (0.92,1.07)	0.12	0.93 (0.85,1.02)	0.333	1.06 (0.94,1.19)	0.0335
rs28558726	53058676	A/G	0.1	0.788	0.99 (0.91,1.08)	0.367	0.95 (0.86,1.06)	0.256	0.92 (0.81,1.06)	0.842
rs9892976	53060329	G/A	0.1	0.788	0.99 (0.91,1.08)	0.367	0.95 (0.86,1.06)	0.256	0.92 (0.81,1.06)	0.842
rs10491163	52816901	G/A	0.23	0.792	0.99 (0.93,1.05)	0.994	1 (0.93,1.07)	0.508	0.97 (0.88,1.07)	0.517
rs7359696	53066189	A/G	0.19	0.794	0.99 (0.93,1.06)	0.162	0.94 (0.87,1.02)	0.729	1.02 (0.92,1.13)	0.13
ch17_pos52975265	52975265	G/C	0.13	0.797	0.99 (0.92,1.07)	0.119	0.93 (0.85,1.02)	0.298	0.94 (0.83,1.06)	0.721
rs4794538	52925502	A/G	0.1	0.804	1.01 (0.93,1.1)	0.942	1 (0.91,1.11)	0.381	1.06 (0.93,1.21)	0.501
rs16955329	52969128	A/C	0.43	0.806	1.01 (0.96,1.06)	0.277	1.03 (0.97,1.1)	0.983	1 (0.92,1.08)	0.216
rs17745332	53047994	G/A	0.19	0.81	0.99 (0.93,1.06)	0.166	0.95 (0.87,1.02)	0.752	1.02 (0.92,1.13)	0.143

rs12952523	53044084	C/A	0.09	0.811	0.99 (0.9,1.08)	0.27	0.94 (0.84,1.05)	0.751	1.02 (0.89,1.18)	0.187
rs17818028	53062755	G/C	0.09	0.812	0.99 (0.91,1.08)	0.405	0.96 (0.86,1.06)	0.198	0.91 (0.79,1.05)	0.684
rs9909390	52839122	T/A	0.08	0.818	1.01 (0.92,1.11)	0.438	0.95 (0.85,1.07)	0.118	1.13 (0.97,1.31)	0.0303
rs244348	53196291	G/A	0.09	0.818	0.99 (0.91,1.08)	0.329	0.95 (0.85,1.06)	0.25	0.92 (0.8,1.06)	0.949
rs12452421	52876664	G/A	0.47	0.818	1.01 (0.96,1.06)	0.96	1 (0.94,1.06)	0.554	1.02 (0.94,1.11)	0.443
rs34946336	53033200	G/A	0.12	0.823	1.01 (0.93,1.09)	0.523	0.97 (0.87,1.07)	0.747	0.98 (0.86,1.12)	0.679
rs17635965	52852700	A/G	0.13	0.824	1.01 (0.93,1.09)	0.815	0.99 (0.9,1.09)	0.406	1.05 (0.93,1.18)	0.097
rs34918041	53048027	G/A	0.19	0.824	0.99 (0.93,1.06)	0.175	0.95 (0.87,1.03)	0.752	1.02 (0.92,1.13)	0.148
rs2877636	53006043	A/G	0.12	0.826	1.01 (0.93,1.09)	0.447	0.96 (0.87,1.06)	0.975	1 (0.88,1.14)	0.419
rs2643025	53059082	G/A	0.02	0.829	0.98 (0.82,1.17)	0.75	1.04 (0.83,1.29)	0.943	1.01 (0.77,1.32)	0.735
rs2907623	52858375	G/A	0.44	0.829	1.01 (0.96,1.06)	0.242	1.04 (0.98,1.1)	0.0799	0.93 (0.86,1.01)	0.0164
rs6504944	52972640	G/A	0.44	0.831	0.99 (0.94,1.05)	0.476	1.02 (0.96,1.09)	0.816	1.01 (0.93,1.1)	0.533
rs17636218	52861905	G/A	0.13	0.832	1.01 (0.93,1.09)	0.948	1 (0.91,1.1)	0.576	1.03 (0.92,1.17)	0.284
rs12940239	53068930	A/G	0.3	0.836	1.01 (0.95,1.06)	0.223	0.96 (0.9,1.03)	0.836	1.01 (0.92,1.1)	0.19
rs16955165	52907475	C/A	0.04	0.837	1.01 (0.89,1.15)	0.825	1.02 (0.86,1.2)	0.202	1.14 (0.93,1.4)	0.321
rs9903810	53016053	T/A	0.12	0.838	1.01 (0.93,1.09)	0.436	0.96 (0.87,1.06)	0.884	0.99 (0.87,1.13)	0.462
rs16955299	52957210	A/G	0.34	0.839	0.99 (0.94,1.05)	0.726	0.99 (0.93,1.06)	0.567	1.03 (0.94,1.12)	0.607
rs28564882	52989598	G/A	0.15	0.843	1.01 (0.94,1.08)	0.286	0.95 (0.88,1.04)	0.749	0.98 (0.88,1.1)	0.494
rs2191231	52874735	G/A	0.13	0.846	1.01 (0.93,1.09)	0.913	0.99 (0.91,1.09)	0.29	1.07 (0.95,1.2)	0.222
rs4794548	53014955	C/G	0.28	0.849	1.01 (0.95,1.06)	0.194	1.05 (0.98,1.12)	0.175	0.94 (0.86,1.03)	0.0446
rs17817829	53010381	A/C	0.12	0.85	1.01 (0.93,1.09)	0.415	0.96 (0.87,1.06)	0.947	1 (0.87,1.13)	0.423
ch17_pos52931737	52931737	A/G	0.1	0.851	1.01 (0.93,1.1)	0.974	1 (0.9,1.11)	0.364	1.06 (0.93,1.21)	0.438
rs8066559	52930583	C/G	0.15	0.852	0.99 (0.92,1.07)	0.612	1.02 (0.94,1.11)	0.114	0.91 (0.81,1.02)	0.068
rs16955153	52890788	A/G	0.04	0.855	0.99 (0.87,1.12)	0.818	0.98 (0.83,1.16)	0.257	1.12 (0.92,1.37)	0.226
rs2332297	52918241	A/G	0.12	0.855	1.01 (0.93,1.09)	0.692	1.02 (0.93,1.12)	0.656	1.03 (0.91,1.16)	0.857
rs35698820	52974086	A/G	0.15	0.857	0.99 (0.93,1.07)	0.359	0.96 (0.88,1.05)	0.956	1 (0.9,1.12)	0.502
rs28658459	52955962	G/A	0.41	0.862	1 (0.95,1.06)	0.448	1.02 (0.96,1.09)	0.998	1 (0.92,1.09)	0.488
rs12948479	52957221	G/A	0.16	0.866	1.01 (0.94,1.08)	0.746	0.99 (0.91,1.07)	0.94	1 (0.9,1.12)	0.905
rs17709877	52871114	C/A	0.1	0.867	0.99 (0.91,1.08)	0.691	1.02 (0.92,1.13)	0.234	0.92 (0.8,1.06)	0.12
rs36024363	52977185	C/G	0.12	0.87	0.99 (0.92,1.07)	0.94	1 (0.91,1.1)	0.941	1 (0.89,1.14)	0.803

rs11871690	53025741	G/A	0.12	0.872	1.01 (0.93,1.09)	0.395	0.96 (0.87,1.06)	0.863	0.99 (0.87,1.13)	0.441
rs28364644	52977353	A/G	0.12	0.875	0.99 (0.92,1.07)	0.989	1 (0.91,1.1)	0.954	1 (0.89,1.14)	0.868
rs2877631	52863321	G/A	0.26	0.877	1 (0.95,1.06)	0.306	1.04 (0.97,1.11)	0.525	0.97 (0.89,1.06)	0.279
rs8067139	52976319	A/C	0.36	0.879	1 (0.94,1.05)	0.432	1.03 (0.96,1.09)	0.589	0.98 (0.9,1.06)	0.385
rs8082622	53177567	G/A	0.09	0.886	0.99 (0.91,1.09)	0.329	0.95 (0.85,1.06)	0.325	0.93 (0.81,1.07)	0.971
rs12936375	53022866	C/A	0.12	0.894	1.01 (0.93,1.09)	0.388	0.96 (0.86,1.06)	0.864	0.99 (0.87,1.13)	0.45
ch17_pos53043196	53043196	G/A	0.12	0.894	1.01 (0.93,1.09)	0.645	0.98 (0.88,1.08)	0.997	1 (0.88,1.14)	0.622
rs4793782	53025342	G/A	0.12	0.896	1.01 (0.93,1.09)	0.388	0.96 (0.86,1.06)	0.868	0.99 (0.87,1.13)	0.446
rs7208123	53006339	G/A	0.24	0.896	1 (0.94,1.06)	0.338	0.97 (0.9,1.04)	0.868	1.01 (0.92,1.11)	0.319
rs2907627	52863699	G/A	0.37	0.896	1 (0.95,1.06)	0.724	1.01 (0.95,1.08)	0.687	1.02 (0.94,1.11)	0.979
rs11869723	53023385	G/A	0.12	0.897	1.01 (0.93,1.09)	0.388	0.96 (0.86,1.06)	0.864	0.99 (0.87,1.13)	0.45
rs11869726	53023489	A/G	0.12	0.897	1.01 (0.93,1.09)	0.388	0.96 (0.86,1.06)	0.864	0.99 (0.87,1.13)	0.45
rs41455544	52989776	C/G	0.12	0.9	1.01 (0.93,1.09)	0.42	0.96 (0.87,1.06)	0.904	0.99 (0.87,1.13)	0.471
rs12949687	52843506	G/A	0.13	0.9	1 (0.92,1.08)	0.77	0.99 (0.9,1.09)	0.661	1.03 (0.91,1.16)	0.185
rs2287136	53038082	G/A	0.12	0.904	1 (0.93,1.09)	0.425	0.96 (0.87,1.06)	0.865	0.99 (0.87,1.13)	0.477
rs9891704	53010755	A/G	0.24	0.905	1 (0.94,1.06)	0.352	0.97 (0.9,1.04)	0.822	1.01 (0.92,1.11)	0.301
rs2024465	53034652	A/G	0.12	0.91	1 (0.93,1.09)	0.423	0.96 (0.87,1.06)	0.868	0.99 (0.87,1.13)	0.472
ch17_pos53024410	53024410	G/A	0.12	0.911	1 (0.93,1.09)	0.423	0.96 (0.87,1.06)	0.863	0.99 (0.87,1.13)	0.477
rs11869638	53043894	A/G	0.12	0.911	1 (0.93,1.09)	0.423	0.96 (0.87,1.06)	0.863	0.99 (0.87,1.13)	0.477
rs11870311	53023891	G/A	0.12	0.911	1 (0.93,1.09)	0.423	0.96 (0.87,1.06)	0.863	0.99 (0.87,1.13)	0.477
rs11871939	53043769	G/A	0.12	0.911	1 (0.93,1.09)	0.423	0.96 (0.87,1.06)	0.863	0.99 (0.87,1.13)	0.477
rs17817865	53032425	G/A	0.12	0.911	1 (0.93,1.09)	0.423	0.96 (0.87,1.06)	0.863	0.99 (0.87,1.13)	0.477
rs35551341	53032644	A/G	0.12	0.911	1 (0.93,1.09)	0.423	0.96 (0.87,1.06)	0.863	0.99 (0.87,1.13)	0.477
rs7220451	52883230	C/A	0.04	0.913	0.99 (0.87,1.13)	0.891	0.99 (0.84,1.16)	0.234	1.13 (0.93,1.38)	0.232
rs34838222	52818321	G/A	0.21	0.915	1 (0.94,1.06)	0.917	1 (0.93,1.08)	0.357	0.95 (0.86,1.05)	0.396
rs12603899	53010098	A/G	0.24	0.915	1 (0.94,1.06)	0.344	0.97 (0.9,1.04)	0.812	1.01 (0.92,1.11)	0.293
rs7209926	53006324	A/G	0.24	0.915	1 (0.94,1.06)	0.357	0.97 (0.9,1.04)	0.868	1.01 (0.92,1.11)	0.332
ch17_pos53242391	53242391	T/A	0.02	0.916	1.01 (0.83,1.23)	0.302	1.14 (0.89,1.45)	0.529	1.1 (0.82,1.49)	0.852
ch17_pos53009280	53009280	C/A	0.24	0.916	1 (0.94,1.06)	0.339	0.97 (0.9,1.04)	0.792	1.01 (0.92,1.11)	0.287
rs2332305	52978674	A/T	0.12	0.919	1 (0.92,1.08)	0.387	0.96 (0.87,1.06)	0.728	0.98 (0.86,1.11)	0.562

rs12949718	52981853	G/A	0.27	0.923	1 (0.95,1.06)	0.339	0.97 (0.9,1.04)	0.988	1 (0.92,1.09)	0.342
rs9900816	53012057	G/A	0.24	0.924	1 (0.94,1.06)	0.347	0.97 (0.9,1.04)	0.806	1.01 (0.92,1.11)	0.289
rs35168324	52980004	A/C	0.12	0.925	1 (0.92,1.08)	0.994	1 (0.91,1.1)	0.897	1.01 (0.89,1.14)	0.82
rs8064660	52975823	G/A	0.36	0.925	1 (0.95,1.06)	0.367	1.03 (0.97,1.1)	0.639	0.98 (0.9,1.07)	0.412
rs17745279	53042743	A/C	0.12	0.927	1 (0.93,1.09)	0.405	0.96 (0.87,1.06)	0.863	0.99 (0.87,1.13)	0.468
rs34391032	53028683	G/A	0.12	0.927	1 (0.93,1.09)	0.417	0.96 (0.87,1.06)	0.87	0.99 (0.87,1.13)	0.461
rs9907961	53012927	C/G	0.24	0.931	1 (0.94,1.06)	0.349	0.97 (0.9,1.04)	0.773	1.01 (0.92,1.11)	0.278
rs12952699	52998340	G/A	0.11	0.935	1 (0.92,1.09)	0.363	0.95 (0.86,1.06)	0.91	0.99 (0.87,1.13)	0.402
rs8069447	53008873	A/C	0.36	0.939	1 (0.95,1.06)	0.222	0.96 (0.9,1.02)	0.87	1.01 (0.93,1.1)	0.178
rs11871985	53043979	G/A	0.12	0.942	1 (0.93,1.09)	0.391	0.96 (0.86,1.06)	0.863	0.99 (0.87,1.13)	0.462
rs9303360	53001456	A/G	0.35	0.944	1 (0.95,1.05)	0.144	0.95 (0.89,1.02)	0.896	1.01 (0.92,1.09)	0.14
rs2332312	52996429	G/A	0.12	0.95	1 (0.92,1.08)	0.354	0.95 (0.86,1.05)	0.793	0.98 (0.86,1.12)	0.483
rs1017099	52879063	A/C	0.47	0.954	1 (0.95,1.05)	0.699	0.99 (0.93,1.05)	0.748	1.01 (0.93,1.1)	0.441
rs12941894	52843777	A/G	0.13	0.955	1 (0.93,1.08)	0.847	0.99 (0.9,1.09)	0.691	1.02 (0.91,1.16)	0.239
rs2907626	52861335	G/A	0.44	0.955	1 (0.95,1.05)	0.426	1.03 (0.96,1.09)	0.0783	0.93 (0.86,1.01)	0.0303
rs244331	53266069	G/A	0.43	0.969	1 (0.95,1.05)	0.46	0.98 (0.92,1.04)	0.067	1.08 (0.99,1.17)	0.0256
rs2191232	52874930	A/C	0.13	0.978	1 (0.93,1.08)	0.73	0.98 (0.9,1.08)	0.299	1.06 (0.95,1.2)	0.166
rs12939352	53007655	A/C	0.36	0.978	1 (0.95,1.06)	0.191	0.96 (0.9,1.02)	0.867	1.01 (0.93,1.1)	0.156
ch17_pos52966078	52966078	A/G	0.09	0.982	1 (0.91,1.1)	0.386	1.05 (0.94,1.17)	0.514	0.95 (0.82,1.1)	0.205
rs8068574	52972247	A/G	0.46	0.982	1 (0.95,1.05)	0.272	1.04 (0.97,1.1)	0.87	1.01 (0.93,1.09)	0.352
rs16955327	52967511	T/A	0.24	0.984	1 (0.94,1.06)	0.366	1.03 (0.96,1.11)	0.418	0.96 (0.87,1.06)	0.143
rs244332	53266012	G/A	0.43	0.987	1 (0.95,1.05)	0.481	0.98 (0.92,1.04)	0.069	1.08 (0.99,1.17)	0.0287
rs9904670	53000707	G/A	0.12	0.988	1 (0.92,1.09)	0.36	0.95 (0.86,1.06)	0.803	0.98 (0.86,1.12)	0.478
rs9915183	53047137	G/A	0.09	0.994	1 (0.92,1.09)	0.545	0.97 (0.87,1.08)	0.278	0.93 (0.81,1.06)	0.744
ch17_pos52963621	52963621	A/G	0.36	0.996	1 (0.95,1.05)	0.875	1.01 (0.94,1.07)	0.785	1.01 (0.93,1.1)	0.946

a: Build 37 coordinates on chromosome 17.

b: Major and minor allele.

c: Minor Allele Frequency.

d: P-value, Odds ratio (OR) and 95% Confidence Interval (CI) for association with Overall Breast Cancer Risk, Estrogen Positive (ER+) and Estrogen Negative (ER-) disease.

e: Case-only analysis, treating subtype status as a dependent variable, models adjusted for principal components and per-study fixed-effects .

Supplementary Table 4: Association result of genotyped markers correlated (r^2 > 0.6) with, and likelihood ratio (RL) of <1:100 relative to rs2787486 with respect to overall Risk among the two populations

			European								Asian							
				Overall Risk [d]		ER+ [d]		ER- [d]		Het [e]		Overall Risk [d]		ER+ [d]		ER- [d]		Het [f]
SNP	Position [a]	Maj/Min [b]	MAF [c]	P-value	OR (95%CI)	P-value	OR (95%CI)	P-value	OR (95%CI)	P-value	MAF [c]	P-value	OR (95%CI)	P-value	OR (95%CI)	P-value	OR (95%CI)	P-value
rs2787497	53176211	A/G	0.27	9.27E-13	0.93 (0.91,0.95)	3.52E-13	0.91 (0.89,0.93)	0.0702	0.96 (0.92,1)	0.007276	0.1	0.611	0.98 (0.9,1.06)	0.177	0.93 (0.84,1.03)	0.194	0.91 (0.8,1.05)	0.933
rs8082622	53177567	G/A	0.27	7.06E-13	0.93 (0.91,0.94)	6.24E-13	0.91 (0.89,0.94)	0.0528	0.96 (0.92,1)	0.011023	0.09	0.886	0.99 (0.91,1.09)	0.329	0.95 (0.85,1.06)	0.325	0.93 (0.81,1.07)	0.971
rs244358	53192231	G/A	0.27	9.68E-13	0.93 (0.91,0.95)	2.97E-13	0.91 (0.89,0.93)	0.0893	0.97 (0.93,1.01)	0.005256	0.09	0.645	0.98 (0.9,1.07)	0.19	0.93 (0.83,1.04)	0.223	0.92 (0.8,1.06)	0.923
rs244353	53194769	G/A	0.29	5.75E-14	0.92 (0.9,0.94)	5.96E-14	0.91 (0.89,0.93)	0.0275	0.96 (0.92,1)	0.015142	0.28	0.0026	0.92 (0.87,0.97)	0.0051	0.91 (0.85,0.97)	0.0443	0.91 (0.83,1)	0.765
rs244348	53196291	G/A	0.27	7.08E-13	0.93 (0.91,0.94)	5.20E-13	0.91 (0.89,0.93)	0.0547	0.96 (0.92,1)	0.010577	0.09	0.818	0.99 (0.91,1.08)	0.329	0.95 (0.85,1.06)	0.25	0.92 (0.8,1.06)	0.949
rs244342	53198407	G/A	0.29	6.73E-14	0.92 (0.9,0.94)	7.48E-14	0.91 (0.89,0.93)	0.03	0.96 (0.92,1)	0.014533	0.28	0.003	0.92 (0.87,0.97)	0.0064	0.91 (0.85,0.97)	0.0393	0.91 (0.83,1)	0.849
rs244338	53200418	G/A	0.29	6.21E-14	0.92 (0.9,0.94)	6.70E-14	0.91 (0.89,0.93)	0.0276	0.96 (0.92,1)	0.015585	0.28	0.0028	0.92 (0.87,0.97)	0.0058	0.91 (0.85,0.97)	0.041	0.91 (0.83,1)	0.798
rs244336	53201502	G/A	0.27	8.80E-13	0.93 (0.91,0.95)	7.60E-13	0.91 (0.89,0.94)	0.047	0.96 (0.92,1)	0.012981	0.09	0.728	0.98 (0.9,1.08)	0.241	0.94 (0.84,1.05)	0.226	0.92 (0.8,1.06)	0.987
rs2628321	53205917	A/G	0.29	5.88E-14	0.92 (0.9,0.94)	7.01E-14	0.91 (0.89,0.93)	0.0277	0.96 (0.92,1)	0.015346	0.28	0.0028	0.92 (0.87,0.97)	0.0054	0.91 (0.85,0.97)	0.0447	0.91 (0.83,1)	0.775
rs2787481	53211110	A/G	0.27	6.44E-13	0.92 (0.91,0.94)	2.93E-13	0.91 (0.89,0.93)	0.0637	0.96 (0.92,1)	0.008094	0.1	0.559	0.98 (0.9,1.06)	0.178	0.93 (0.84,1.03)	0.151	0.91 (0.79,1.04)	0.857
rs244315	53214654	G/A	0.27	4.72E-13	0.92 (0.91,0.94)	4.27E-13	0.91 (0.89,0.93)	0.0493	0.96 (0.92,1)	0.010962	0.1	0.599	0.98 (0.9,1.06)	0.182	0.93 (0.84,1.03)	0.173	0.91 (0.79,1.04)	0.893
rs244317	53216985	T/A	0.27	5.79E-13	0.92 (0.91,0.94)	2.74E-13	0.91 (0.89,0.93)	0.0649	0.96 (0.92,1)	0.007933	0.1	0.578	0.98 (0.9,1.06)	0.185	0.93 (0.84,1.03)	0.156	0.91 (0.79,1.04)	0.857
rs2628316	53219837	A/G	0.29	6.00E-14	0.92 (0.9,0.94)	1.17E-13	0.91 (0.89,0.93)	0.0276	0.96 (0.92,1)	0.015608	0.28	0.0151	0.93 (0.88,0.99)	0.0115	0.91 (0.85,0.98)	0.19	0.94 (0.86,1.03)	0.501
rs244319	53222874	G/A	0.29	2.19E-13	0.93 (0.91,0.94)	4.18E-13	0.91 (0.89,0.94)	0.028	0.96 (0.92,1)	0.021933	0.3	0.0263	0.94 (0.89,0.99)	0.022	0.92 (0.86,0.99)	0.309	0.96 (0.88,1.04)	0.452
rs244320	53222920	G/A	0.29	2.19E-13	0.93 (0.91,0.94)	4.18E-13	0.91 (0.89,0.94)	0.028	0.96 (0.92,1)	0.021933	0.3	0.0233	0.94 (0.89,0.99)	0.0194	0.92 (0.86,0.99)	0.312	0.96 (0.88,1.04)	0.424
rs10432032	53224088	A/G	0.29	2.47E-13	0.93 (0.91,0.94)	5.33E-13	0.91 (0.89,0.94)	0.0281	0.96 (0.92,1)	0.023436	0.3	0.0203	0.94 (0.88,0.99)	0.0197	0.92 (0.86,0.99)	0.202	0.94 (0.86,1.03)	0.565
rs2628315	53226622	G/A	0.29	9.02E-14	0.92 (0.9,0.94)	1.78E-13	0.91 (0.89,0.93)	0.0259	0.96 (0.92,0.99)	0.018409	0.29	0.0059	0.92 (0.87,0.98)	0.0066	0.91 (0.85,0.97)	0.0778	0.92 (0.84,1.01)	0.715

a: Build 37 coordinates on chromosome 17.
b: Major and Minor allele.
c: Minor Allele Frequency.
d: P-value, Odds ratio (OR) and 95% Confidence Interval (CI) for association with Overall Breast Cancer Risk, Estrogen Positive (ER+) and Estrogen Negative (ER-) disease.
e: Case-only analysis, treating subtype status as a dependent variable, models adjusted for principal components and per-study fixed-effects.

Supplementary Table 5: Candidate causal SNPs with overlap of regulatory element

**Variant** [a]	**rsID** [b]	**TF** [c]	**DB_score** [d]	GTEx all [e]	GTEx breast [f]	RefGene [g]	RefGeneTSS [h]	target_gene [i]	Functional [j]
rs2787497	rs2787497		5	COX11, COX11|TOM1L1, CTC-462L7.1, STXBP4	STXBP4	STXBP4	STXBP4|130085	HLF:IMR90:Hi-C	INTRON
rs8082622	rs8082622	AIRE_2|-, Foxj1_1|-, Klf7|+, NF-Y_known3|-, RXRA_disc5|+	5	COX11, COX11|TOM1L1, CTC-462L7.1, STXBP4	STXBP4	STXBP4	STXBP4|131441	HLF:IMR90:Hi-C	INTRON
rs2529510	rs2529510	BATF_disc2|-, CEBPB_known4|-, Ik-1_1|-, Ik-1_2|-, Irf_disc6|-, Sox_4|-	6	COX11, CTC-462L7.1, STXBP4		STXBP4	STXBP4|135920		INTRON
rs244373	rs244373	BRCA1_known2|+, CACD_1|-, ERalpha-a_disc2|+,RAR|-, RXRA_disc1|-, SF1|+	6	CTC-462L7.1, STXBP4	STXBP4	STXBP4	STXBP4|138823		INTRON
rs244358	rs244358	SF1|+, TAL1_disc1|-	7	COX11, CTC-462L7.1, STXBP4	STXBP4	STXBP4	STXBP4|146105		INTRON
rs187242	rs187242	HNF1_7|-, Mef2_known1|+, Mef2_known6|+, Sox_15|+,Sox_2|+	7	CTC-462L7.1, STXBP4	STXBP4	STXBP4	STXBP4|146820		INTRON
rs244353	rs244353	HNF4_known1|+, PPAR_3|+, RFX5_known2|+, RXRA_known5|-	1f	CTC-462L7.1, STXBP4	STXBP4	STXBP4	HLF|147551	STXBP4:BI_CD4_ Memory_Primary_7pool:Hnisz, HLF:HepG2:PreSTIGE, STXBP4:HUVEC:Hnisz	INTRON

rs244348	rs244348	BCL_disc7|-,<br>Cdc5|+,Cdx|+,<br>Isl2|+,<br>Mef2_known3|+,<br>NRSF_disc7|-	6	COX11,<br>COX11|TOM1L1,<br>CTC-462L7.1,<br>STXBP4	STXBP4	STXBP4	HLF|146029		INTRON
rs244342	rs244342	HDAC2_disc6|+	7	COX11|TOM1L1,<br>CTC-462L7.1,<br>STXBP4	STXBP4	STXBP4	HLF|143913		INTRON
rs244338	rs244338	CDP_7|+,<br>CEBPG|-,<br>SRF_known5|-	6	CTC-462L7.1,<br>STXBP4	STXBP4	STXBP4	HLF|141902		INTRON
rs244337	rs244337	Foxj2_2|+,<br>HES1|+,<br>RFX5_known1|+,<br>ZBRK1|+	7	CTC-462L7.1,<br>STXBP4	STXBP4	STXBP4	HLF|141196	HLF:ColonCrypt:PreSTIGE	INTRON
rs244336	rs244336	Mrg1::Hoxa9_2|-,<br>RREB-1_1|+,<br>SIX5_known1|-,<br>p300_disc7|-	7	COX11,<br>CTC-462L7.1,<br>STXBP4	STXBP4	STXBP4	HLF|140818	HLF:ColonCrypt:PreSTIGE	INTRON
chr17:53205761:D	rs11298913			COX11,<br>CTC-462L7.1,<br>STXBP4,<br>TOM1L1	STXBP4	STXBP4	HLF|136559		INTRON
rs2628321	rs2628321		7	COX11,<br>COX11|TOM1L1,<br>CTC-462L7.1,<br>STXBP4	STXBP4	STXBP4	HLF|136403		INTRON
rs2787486	rs2787486	GR_disc5|+,<br>Maf_disc2|-,<br>RXRA_known3|-,<br>VDR_2|+	7	CTC-462L7.1,<br>STXBP4	STXBP4	STXBP4	HLF|132546	HLF:IMR90:Hi-C	INTRON
rs2787481	rs2787481	Pax-6_1|-	1f	COX11,<br>COX11|TOM1L1,<br>CTC-462L7.1,<br>STXBP4	STXBP4	STXBP4	HLF|131210	HLF:IMR90:Hi-C	INTRON

rs244315	rs244315	CAC-binding-protein|-, CTCFL_disc1|-, EWSR1-FLI1|+, MAZ|-, PU.1_disc3|+, RXRA_known6|+, SP1_disc3|-, STAT_disc7|-, TATA_disc7|+, TCF12_disc5|+, TFII-I|-, ZNF263_disc1|+, Zfp281|-	6	COX11, CTC-462L7.1, STXBP4, TOM1L1	STXBP4	STXBP4	HLF|127666		INTRON
rs244317	rs244317	Foxf2|+, Sin3Ak-20_disc6|+	1f	COX11, COX11|TOM1L1, CTC-462L7.1, STXBP4, TOM1L1	STXBP4	STXBP4	HLF|125335		INTRON
rs2628316	rs2628316	HNF1_2|-, PU.1_disc3|+	6	COX11, COX11|TOM1L1, CTC-462L7.1, STXBP4	STXBP4	STXBP4	HLF|122483		INTRON
chr17:53221365:I	rs200876987	CTCF_disc10|-, Foxp1|-,Gm397|+, Myf_2|+, RREB-1_2|-		CTC-462L7.1, STXBP4	STXBP4	STXBP4	HLF|120955		INTRON
rs244318	rs244318	CTCF_disc10|-, Foxp1|-, Gm397|+, Myf_2|+, RREB-1_1|-, RREB-1_2|-	6	COX11, CTC-462L7.1, STXBP4	STXBP4	STXBP4	HLF|120952		INTRON
rs244319	rs244319	Mrg_2|+, Sin3Ak-20_disc3|+, Tgif1_1|-	4	COX11, COX11|TOM1L1, CTC-462L7.1, STXBP4	STXBP4	STXBP4	HLF|119446		INTRON
rs244320	rs244320	HNF6|+	4	COX11, CTC-462L7.1, STXBP4	STXBP4	STXBP4	HLF|119400		INTRON

rs244321	rs244321		7	COX11, CTC-462L7.1, STXBP4	STXBP4	STXBP4	HLF|118322	INTRON
rs10432032	rs10432032	Elf3|+, Evi-1_4|-, Foxp1|-, GATA_disc3|+, GATA_known8|+, HDAC2_disc6|-, Ik-2_3|+, Irf_disc3|+, Irf_known9|-, NF-AT|+, TATA_disc7|+, Zfp105|-, p300_disc5|+	6			STXBP4	HLF|118232	INTRON
rs244322	rs244322	STAT_known10|-	5	COX11, COX11|TOM1L1, CTC-462L7.1, STXBP4	STXBP4	STXBP4	HLF|117561	INTRON
rs2628315	rs2628315	Pax-4_4|+	6	COX11, COX11|TOM1L1, CTC-462L7.1, STXBP4	STXBP4	STXBP4	HLF|115698	INTRON
rs244294	rs244294	Cdx2_1|-, Cdx2_2|-, Hoxa10|-, Hoxa9|-, Hoxb9|-, Hoxc10|+, Hoxc9|-, Hoxd10|-, Nkx6-1_1|-, PLZF|+	6	COX11, COX11|TOM1L1, CTC-462L7.1, STXBP4	STXBP4	STXBP4	HLF|113777	INTRON

	**Short**	**Long**	**Description**
a	Variant	variant	Variant ID
b	rsID	rsID	rsID for top snp at locus
c	TF	TF_motifs|delta	ENCODE_PWM alteration. The delta shown as "+" (PWM strengthened) or "-" (weakened)

d	DB_score	RegulomeDB_score	RegulomeDB score. Score (http://regulomedb.org/help#score). Scale: 1 is most ilkely to be functional, to 7 = least likely.
e	GTEx all	eQTL_target_all	SNP reported to be associated with expression in studies including GTEx.v6, GEUVADIS, Westra Nat Genetics. All tissue types included.
f	GTEx breast	eQTL_GTEx.breast	SNP is associated with expression in GTEx breast samples (N =186)
g	RefGene	Overlapping_RefGene	SNP falls within an annotated RefGene (within transcribed sequence)
h	RefGeneTSS	RefGeneTSS|distance	Distance to nearest refseq transcription start site
i	target_gene	target_gene_prediction	SNP lies in a putative regulatory element predicted to regulated the listed gene, in ANY cell type, reported by methods IM-PET, Hnisz, PreSTIGE, Chia-PET, Hi-C, 3C, 4C, 5C, FANTOM5. Format = target:cell:method,,,target:cell:method,,,target:cell:method1,method2,,,......
j	Functional	functional_annotation	refgene functional annotation

Supplementary Table 6. Summary of association between 17q22 breast cancer risk iCHAV and allelic imbalance of genes within 1Mb up- and downstream flanking region. Levene's Test was used to evaluate equality of variances between heterozygote (CT) and homozygote (CC and TT) samples. The marker SNPs which passed the criteria did not have enough coverage *for KIF2B, TMEM100,* and *ANKFN1* to be included in the analysis.

iCHAV marker (position)	Target Gene	Number of Samples (CC, CT, TT)	P-value
rs2787481 (chr17: 53211110)	TOM1L1	84, 74, 5	0.081
rs2787481 (chr17: 53211110)	COX11	84, 78, 5	0.032*
rs2787481 (chr17: 53211110)	STXBP4	53, 50, 0	0.671
rs2787481 (chr17: 53211110)	HLF	49, 28, 10	0.484
rs2787481 (chr17: 53211110)	MMD	114, 84, 17	0.120
rs2787481 (chr17: 53211110)	PCTP	67, 46, 9	0.191

* marginal statistical significant P-value (<0.05)

Supplementary Table 7: eQTL analysis from the METABRIC study (FDR-adjusted P values)

Top breast cancer hit with each gene

SNP	ilmn	beta	tstat	pval	fdr	pos	info	gene
rs2787486	ILMN_1666280	-0.113065	-3.836345	0.0001921	0.0506373	53209774	0.971	COX11
rs2787486	ILMN_1802642	-0.1114234	-2.195578	0.0298564	0.9843687	53209774	0.971	TOM1L1
rs2787486	ILMN_1802257	-0.0697964	-1.944933	0.0538932	1	53209774	0.971	PCTP
rs2787486	ILMN_1811239	-0.0399775	-1.877066	0.0626997	1	53209774	0.971	ANKFN1
rs2787486	ILMN_1707391	0.0343524	1.232992	0.2197537	1	53209774	0.971	STXBP4
rs2787486	ILMN_1652181	0.0255915	1.180756	0.2398069	1	53209774	0.971	KIF2B
rs2787486	ILMN_1733937	0.1963861	1.046407	0.2972709	1	53209774	0.971	MMD
rs2787486	ILMN_1722829	-0.0551331	-0.9441325	0.3468139	1	53209774	0.971	HLF
rs2787486	ILMN_2135537	0.0099096	0.6348671	0.5266057	1	53209774	0.971	KIF2B
rs2787486	ILMN_1698259	0.0345281	0.311061	0.7562413	1	53209774	0.971	TMEM100

Top eQTL with each gene

SNP	ilmn	beta	tstat	pval	fdr	pos	info	gene
rs138326143	ILMN_1666280	1.190773	5.556613	1.44E-07	0.0029157	53408414	0.993	COX11
rs186663197	ILMN_1802642	32.71718	4.50583	0.0000143	0.0084882	53156559	0.037	TOM1L1
rs187880135	ILMN_2135537	7.352602	4.410875	0.0000211	0.0103592	52762294	0.044	KIF2B
rs74920690	ILMN_1652181	0.1790671	4.058103	0.0000839	0.0295929	52855066	0.874	KIF2B
rs244383	ILMN_1811239	-37.61968	-4.0566	0.0000844	0.0295929	53300592	0.303	ANKFN1
rs184628656	ILMN_1802257	57.6242	3.716124	0.0002969	0.0667873	52798123	0.051	PCTP
rs183917963	ILMN_1707391	2.213403	3.605547	0.0004393	0.0844782	52572748	0.139	STXBP4
chr1752688378I	ILMN_1698259	1.659709	3.551286	0.0005308	0.0976632	52688378	0.64	TMEM100
rs189007317	ILMN_1722829	0.4152539	3.515306	0.0006011	0.1044669	52506510	0.665	HLF
rs8065301	ILMN_1733937	-0.921983	-3.088207	0.0024518	0.2716971	53428677	1	MMD

Supplementary Table 8: GTEx findings; tissues in which there is a significant eQTL for rs244353

Gene Symbol	SNP Id	P-Value	Effect Size	Tissue
STXBP4	rs244353	7.10E-16	-0.39	Adipose - Subcutaneous
STXBP4	rs244353	5.70E-09	-0.45	Adipose - Visceral (Omentum)
STXBP4	rs244353	7.00E-07	-0.39	Artery - Aorta
STXBP4	rs244353	9.20E-20	-0.43	Artery - Tibial
STXBP4	rs244353	1.3E-06	-0.39	Breast - Mammary Tissue
STXBP4	rs244353	2.00E-10	-0.41	Cells - Transformed fibroblasts
STXBP4	rs244353	7.70E-11	-0.37	Lung
STXBP4	rs244353	4.90E-12	-0.36	Nerve - Tibial
STXBP4	rs244353	6.00E-07	-0.34	Skin - Sun Exposed (Lower leg)
STXBP4	rs244353	9.30E-11	-0.35	Thyroid

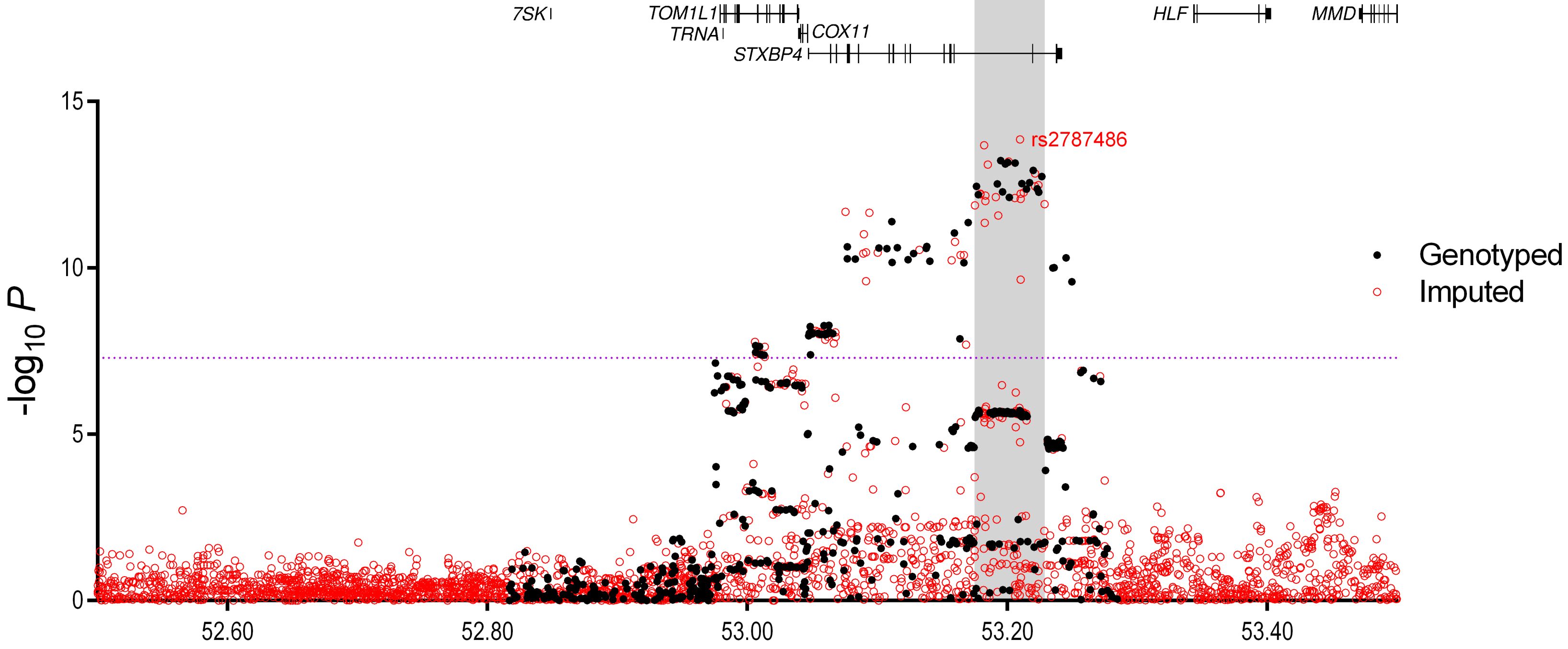

7SK
TOM1L1
TRNA
COX11
STXBP4
HLF
MMD
rs2787486
-log10 P
15
10
5
0
52.60
52.80
53.00
53.20
53.40
Genotyped
Imputed

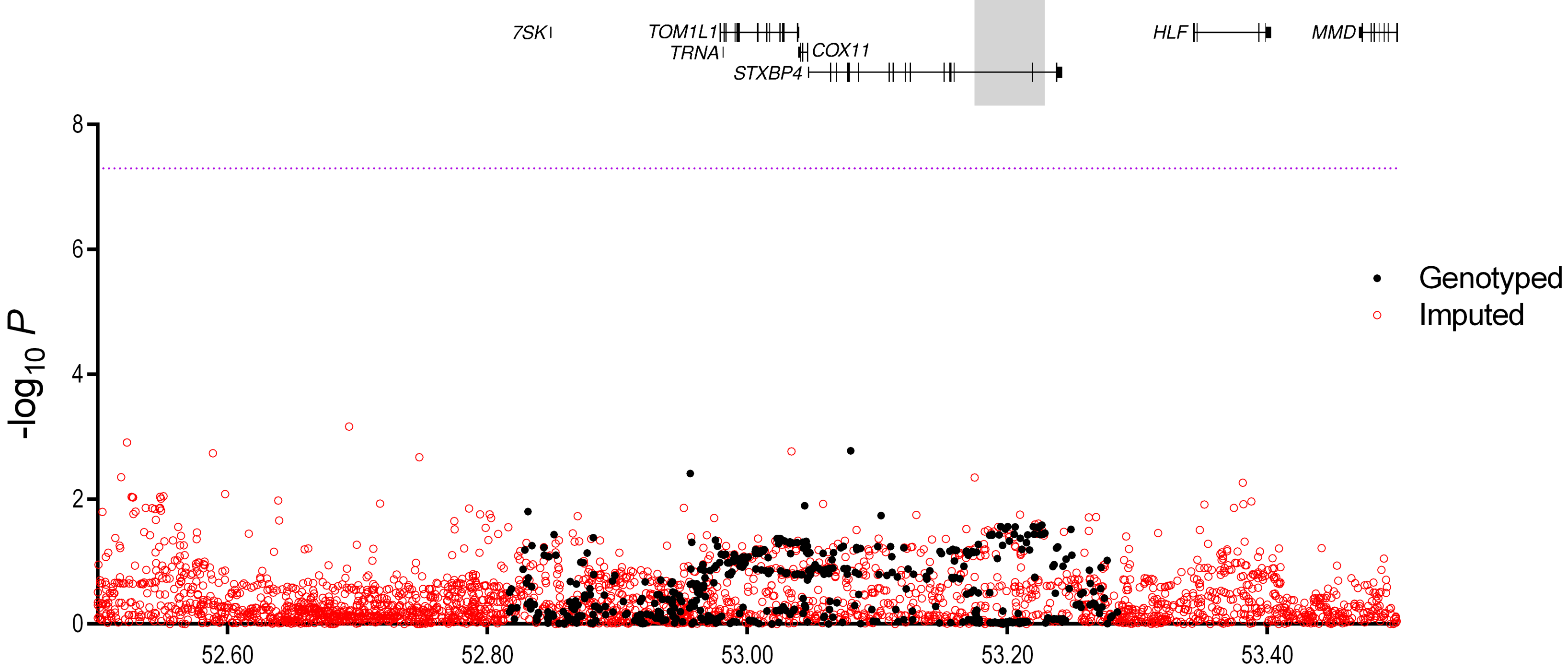
7SK
TOM1L1
TRNA
COX11
STXBP4
HLF
MMD
8
6
4
2
0
-log10 P
52.60
52.80
53.00
53.20
53.40
Genotyped
Imputed

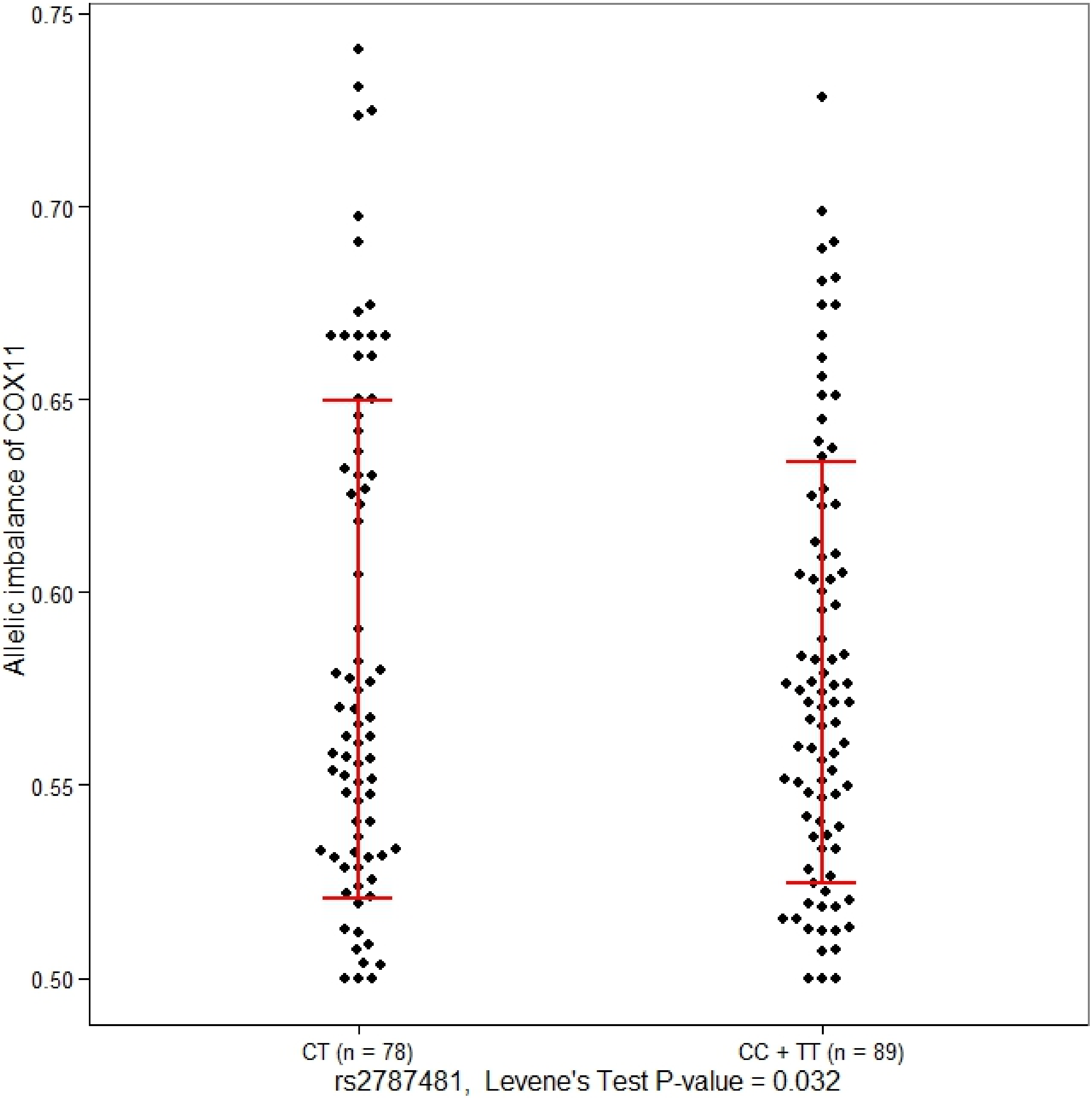
0.75
0.70
0.65
0.60
0.55
0.50
Allelic imbalance of COX11
CT (n = 78)
CC + TT (n = 89)
rs2787481, Levene's Test P-value = 0.032

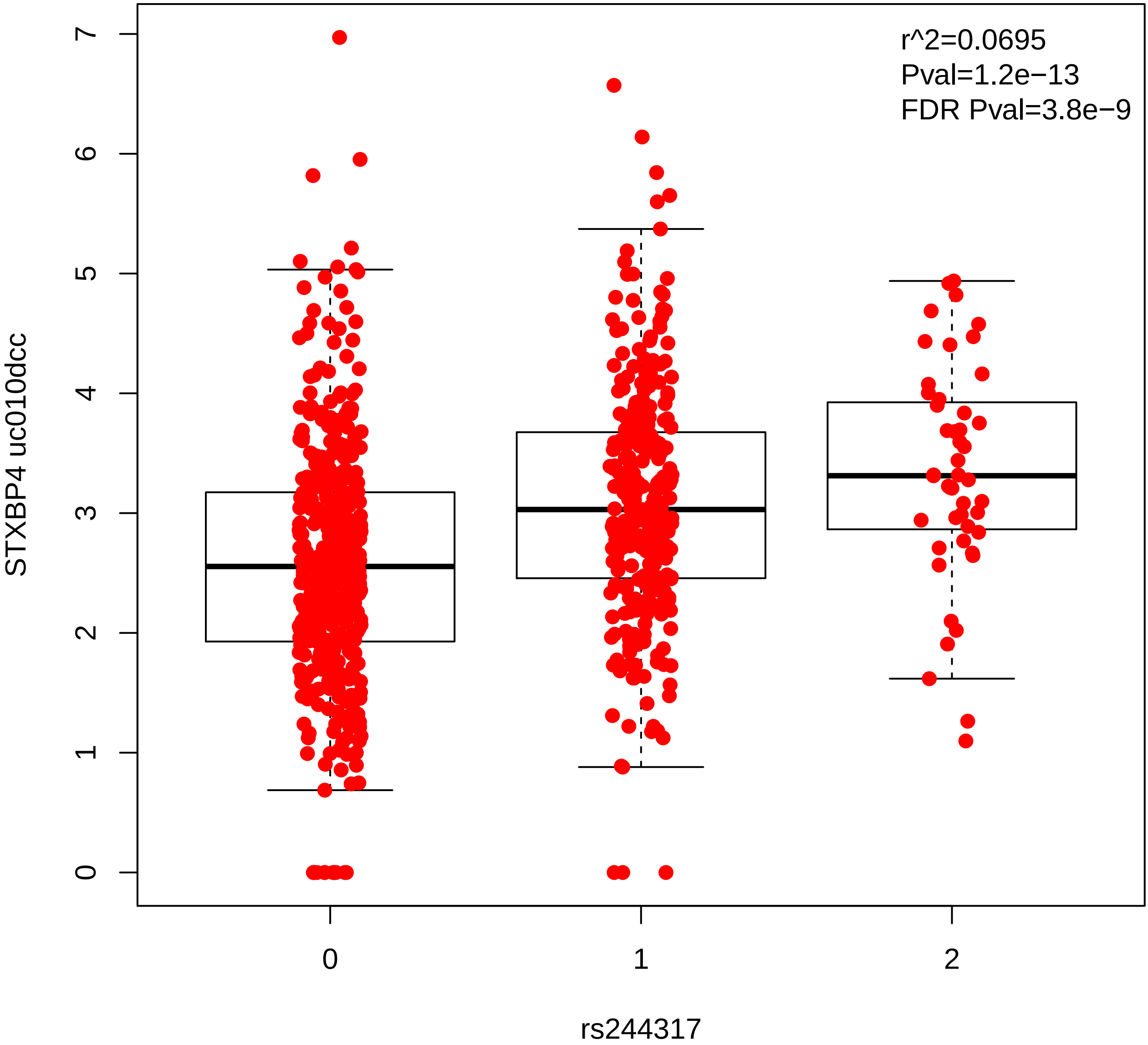

r^2=0.0695
Pval=1.2e−13
FDR Pval=3.8e−9
STXBP4 uc010dcc
rs244317
0
1
2
3
4
5
6
7
0
1
2

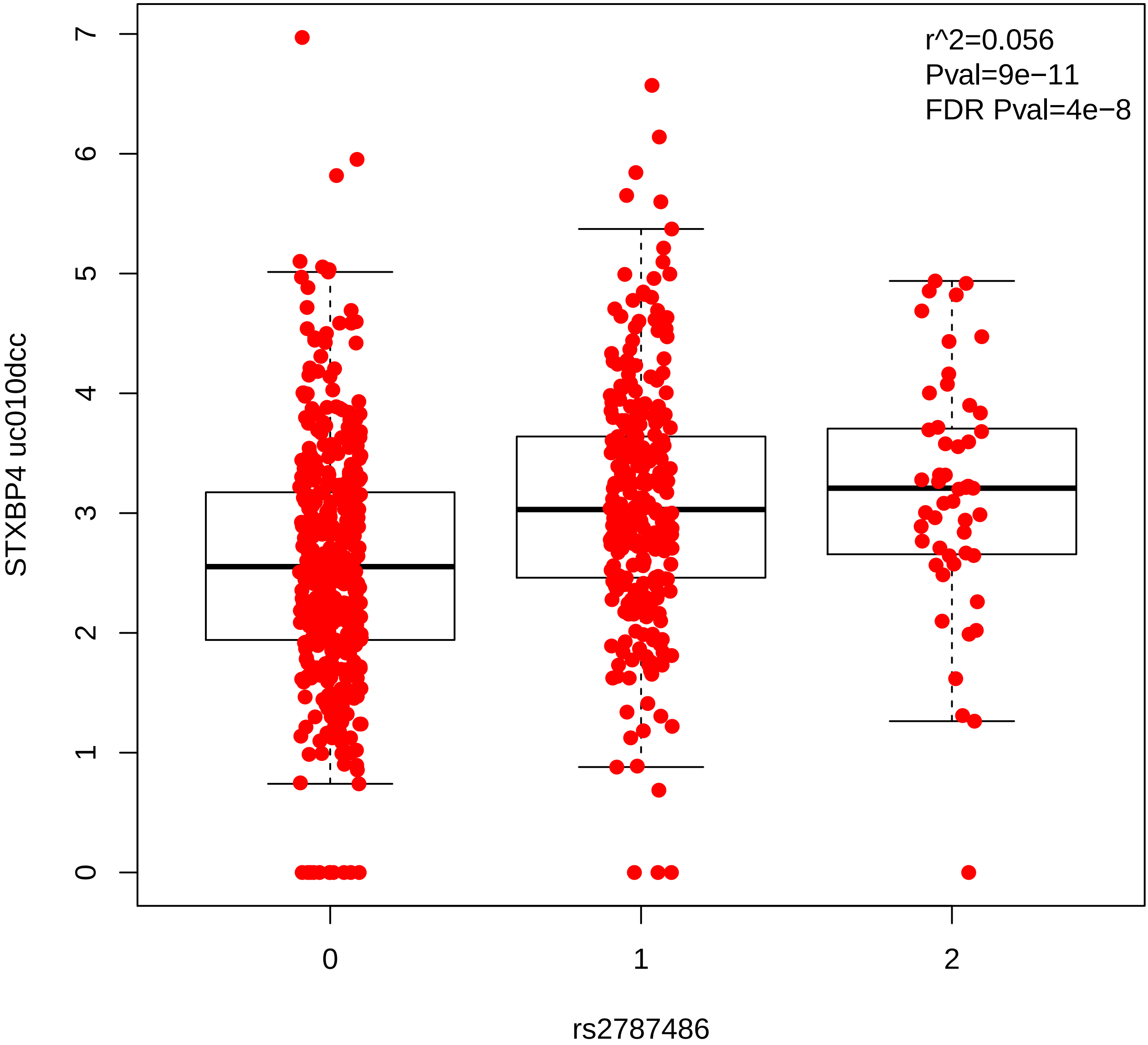
r^2=0.056
Pval=9e−11
FDR Pval=4e−8
STXBP4 uc010dcc
rs2787486
0
1
2
3
4
5
6
7

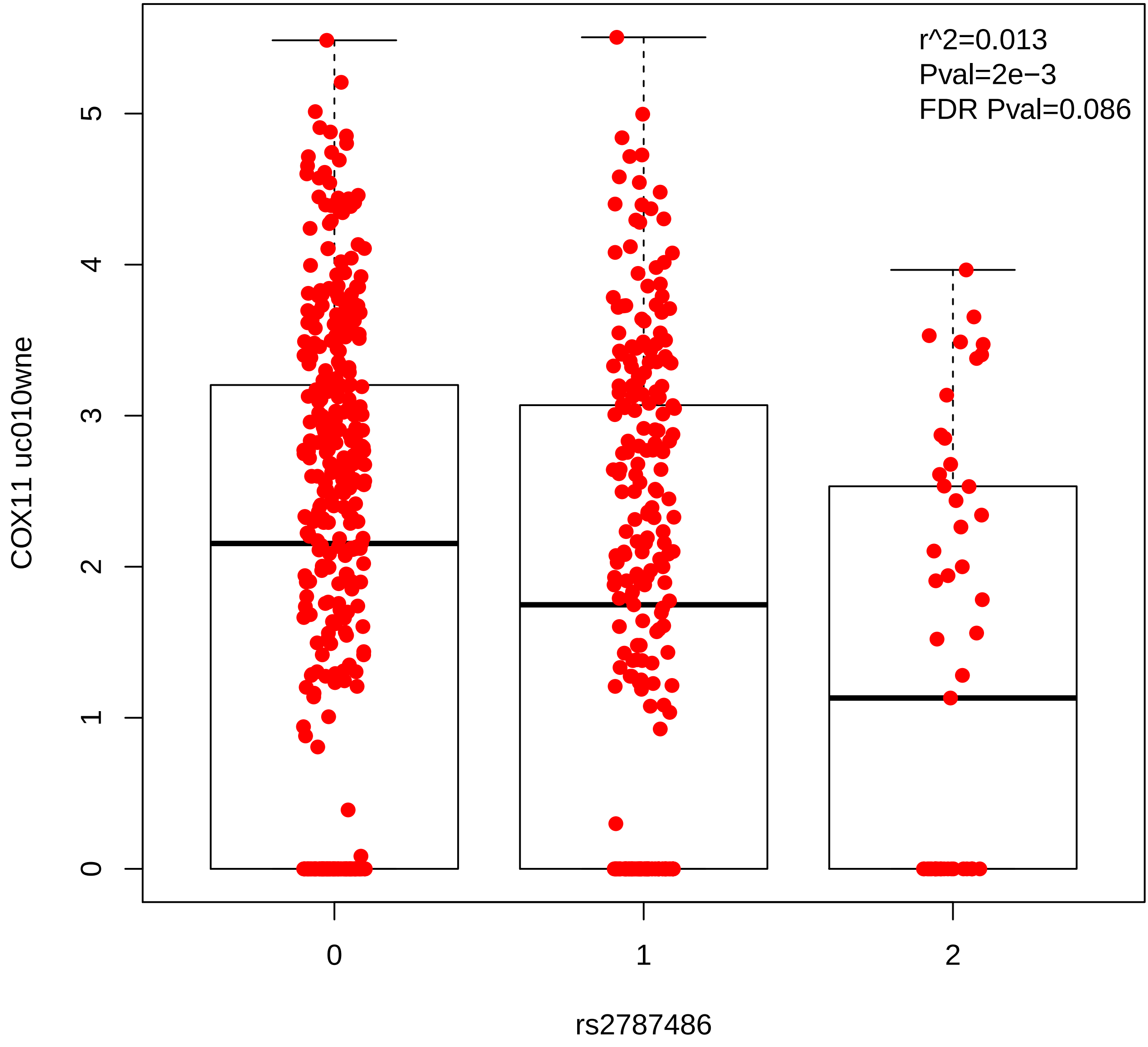
r^2=0.013
Pval=2e−3
FDR Pval=0.086
COX11 uc010wne
rs2787486
0
1
2
3
4
5
0
1
2

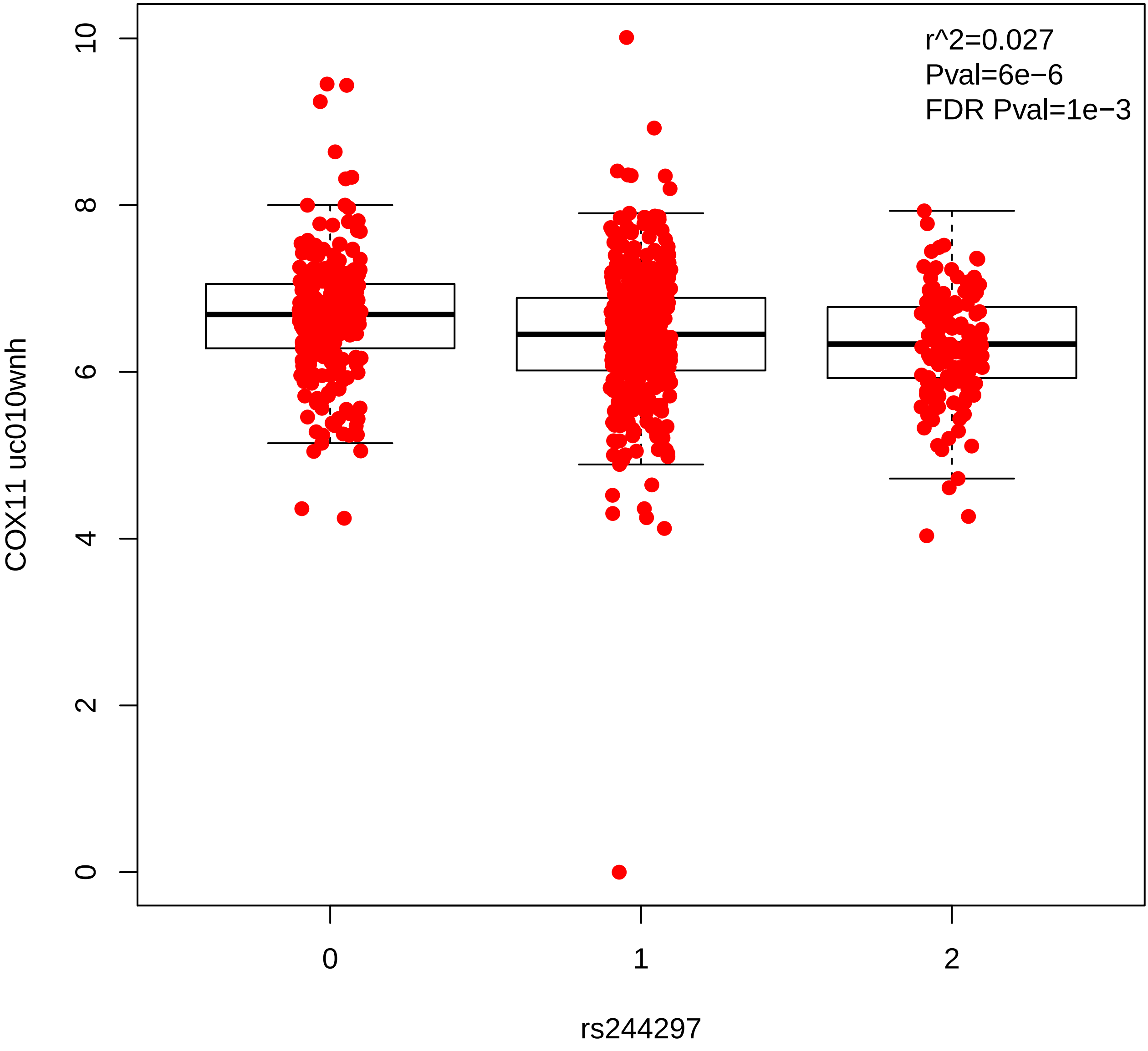
r^2=0.027
Pval=6e−6
FDR Pval=1e−3
COX11 uc010wnh
rs244297
0
2
4
6
8
10
0
1
2

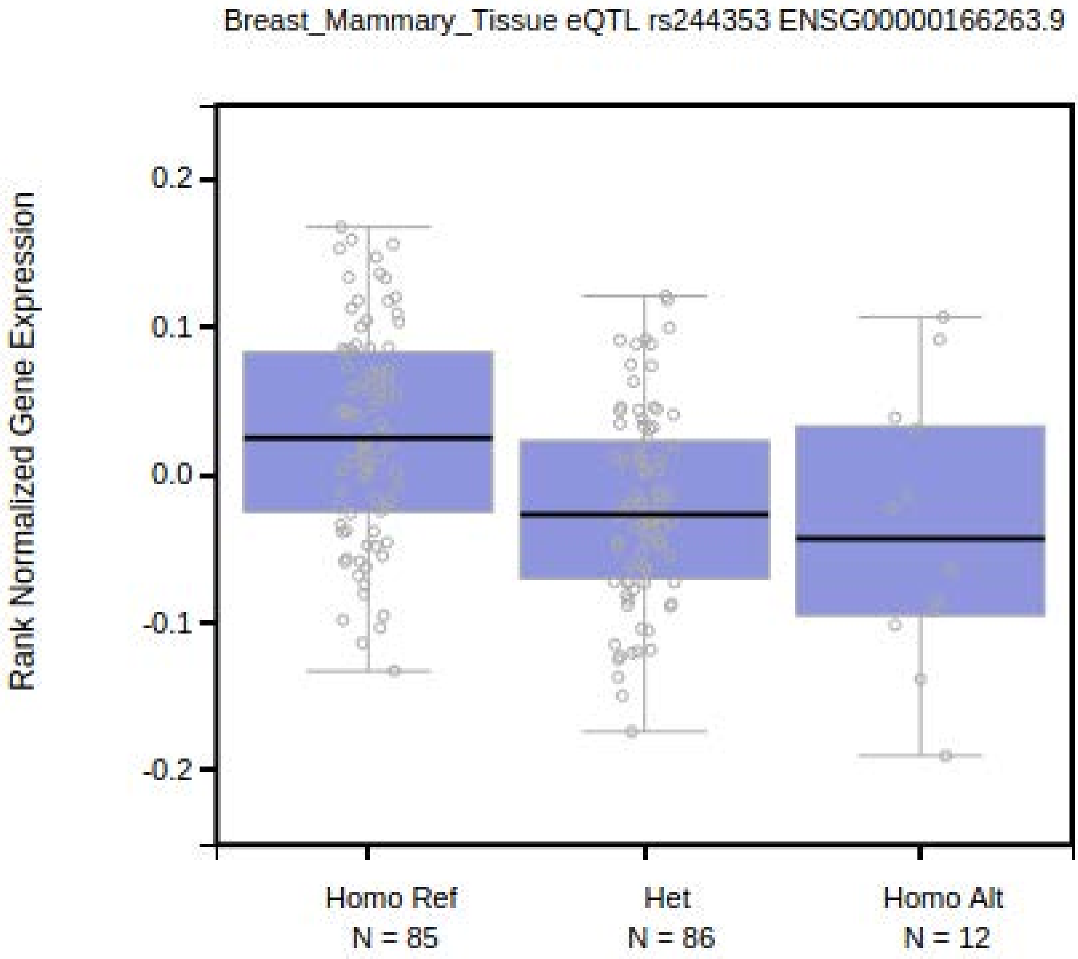
Breast_Mammary_Tissue eQTL rs244353 ENSG00000166263.9
Rank Normalized Gene Expression
0.2
0.1
0.0
-0.1
-0.2
Homo Ref
N = 85
Het
N = 86
Homo Alt
N = 12
